# Ammonia–Borane Dehydrogenation Catalyzed by Dual-Mode Proton-Responsive Ir-CNN^H^ Complexes

**DOI:** 10.1021/acs.inorgchem.1c03056

**Published:** 2021-11-16

**Authors:** Isabel Ortega-Lepe, Andrea Rossin, Práxedes Sánchez, Laura L. Santos, Nuria Rendón, Eleuterio Álvarez, Joaquín López-Serrano, Andrés Suárez

**Affiliations:** † Instituto de Investigaciones Químicas (IIQ), Departamento de Química Inorgánica and Centro de Innovación en Química Avanzada (ORFEO−CINQA). CSIC and Universidad de Sevilla. Avda. Américo Vespucio 49, 41092 Sevilla, Spain; ‡ Istituto di Chimica dei Composti Organometallici - Consiglio Nazionale delle Ricerche (ICCOM - CNR). Via Madonna del Piano 10, 50019, Sesto Fiorentino Italy

## Abstract

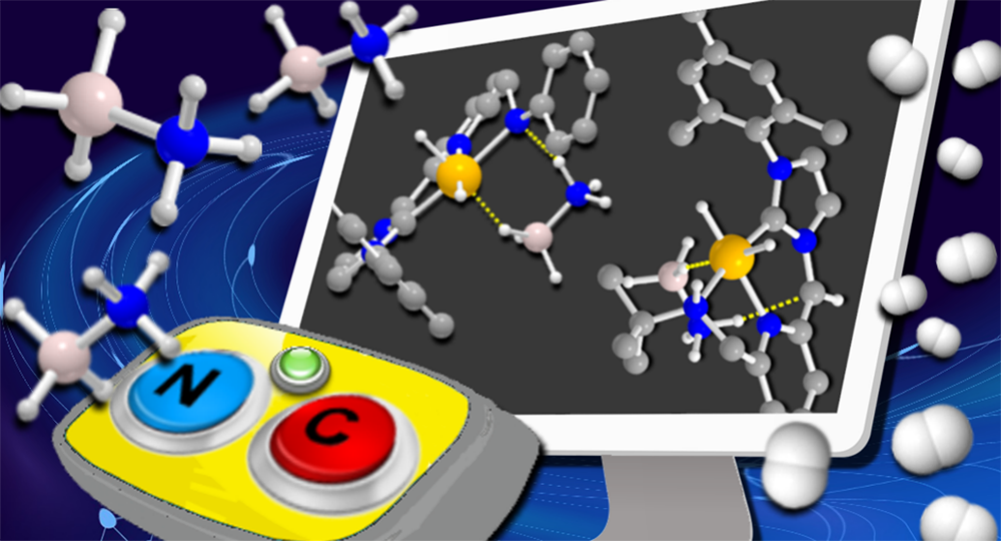

Metal complexes incorporating proton-responsive ligands have been proved to be superior catalysts in reactions involving the H_2_ molecule. In this contribution, a series of Ir^III^ complexes based on lutidine-derived CNN^H^ pincers containing N-heterocyclic carbene and secondary amino NHR [R = Ph (**4a**), *t*Bu (**4b**), benzyl (**4c**)] donors as flanking groups have been synthesized and tested in the dehydrogenation of ammonia–borane (NH_3_BH_3_, AB) in the presence of substoichiometric amounts (2.5 equiv) of *t*BuOK. These preactivated derivatives are efficient catalysts in AB dehydrogenation in THF at room temperature, albeit significantly different reaction rates were observed. Thus, by using 0.4 mol % of **4a**, 1.0 equiv of H_2_ per mole of AB was released in 8.5 min (turnover frequency (TOF_50%_) = 1875 h^–1^), while complexes **4b** and **4c** (0.8 mol %) exhibited lower catalytic activities (TOF_50%_ = 55–60 h^–1^). **4a** is currently the best performing Ir^III^ homogeneous catalyst for AB dehydrogenation. Kinetic rate measurements show a zero-order dependence with respect to AB, and first order with the catalyst in the dehydrogenation with **4a** (−d[AB]/d*t* = *k*[**4a**]). Conversely, the reaction with **4b** is second order in AB and first order in the catalyst (−d[AB]/d*t* = *k*[**4b**][AB]^2^). Moreover, the reactions of the derivatives **4a** and **4b** with an excess of *t*BuOK (2.5 equiv) have been analyzed through NMR spectroscopy. For the former precursor, formation of the iridate **5** was observed as a result of a double deprotonation at the amine and the NHC pincer arm. In marked contrast, in the case of **4b**, a monodeprotonated (at the pincer NHC-arm) species **6** is observed upon reaction with *t*BuOK. Complex **6** is capable of activating H_2_ reversibly to yield the trihydride derivative **7**. Finally, DFT calculations of the first AB dehydrogenation step catalyzed by **5** has been performed at the DFT//MN15 level of theory in order to get information on the predominant metal–ligand cooperation mode.

## Abstract 2

Iridium complexes based on CNN^H^ ligands containing two potential proton-responsive sites—a lutidine scaffold and a secondary amino group—have been tested in the dehydrogenation of ammonia-borane. Upon reaction with base, depending on the amino group acidity, mono- or doubly deprotonated species exhibiting significantly different catalytic activities were observed.

## Introduction

The controlled release of H_2_ from high content hydrogen compounds used as H_2_-storage systems is paramount to the use of this gas as an energy vector in the so-called Hydrogen Economy.^1,2^ In this context, ammonia–borane (NH_3_BH_3_, AB) has received increasing attention as a hydrogen storage material because of its high available hydrogen content (19.6 wt % H), moisture kinetic stability and easy H_2_ thermal release.^3^ In addition, AB dehydrogenation renders B–N oligomers and polymers as byproducts that are suitable starting materials for the synthesis of boron nitride (BN) ceramics.^4^ Although AB dehydrogenation can be performed under simple thermal conditions,^5^ insufficient kinetic control of the process leads to the formation of ill-defined B–N containing solids. This is a serious drawback for the H_2_-depleted material recycling. As a consequence, catalytic approaches to the H_2_ release from AB have been envisaged. The use of a catalyst allows for a fine control of the rate and extent of H_2_ production, as well as the nature of the H_2_-depleted byproducts. In addition, the H_2_ release temperature is lowered, with respect to pure AB, and the process can be performed with a lower energetic impact.

**Figure 1:**
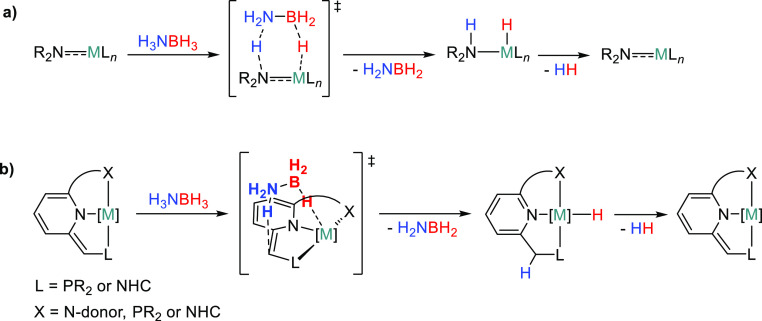
Proposed mechanisms for the first step of ammonia–borane dehydrogenation with transition-metal complexes based on ligands containing Brønsted basic sites.

Catalysts based on transition metals have provided fast kinetics and a large extent of H_2_ release under relatively mild reaction conditions.^6^ Particularly, metal complexes stabilized by pincer ligands have been widely investigated because of the enhanced catalyst stability provided by the κ^3^–tridentate ligand coordination.^7−16^ Moreover, since ammonia–borane is a polar molecule with hydridic (BH) and acidic (NH) hydrogen atoms, transition-metal complexes based on ligands containing Brønsted basic sites have been shown to be particularly adequate to promote H_2_ release from AB through the transfer of the hydridic BH to the acidic metal center and the NH proton to the basic ligand functionality (bifunctional catalysts).^13−19^ Prevalent examples of such systems are metal complexes based on ligands bearing secondary amine donors,^20^ which are capable of getting involved in reversible metal-amine/metal-amido interconversion (Figure 1a).^13−15,17,18^

Similarly, metal complexes incorporating picoline- or lutidine-derived pincer ligands are able to participate in ligand-assisted H–X (X = H, C, O, N, B) bond activation upon deprotonation of the (acidic) CH_2_ pincer side-arms and concomitant dearomatization of the N-containing ring (Figure 1b).^21^ Although these derivatives have been found to be efficient catalysts in a plethora of hydrogenation reactions of polar organic substrates and alcohol dehydrogenation reactions, their application in AB dehydrogenation has been limitedly explored.^16^ Moreover, development of homogeneous catalytic systems based on lutidine-derived ligands has been mainly focused on phosphine-containing PNX (X = phosphine or N-donor) pincers, albeit substitution of the flanking PR_2_ groups by another strong σ-donor such as N-heterocyclic carbenes (NHCs) has also been briefly addressed. Upon deprotonation of the methylene CH_2_–NHC arms, these organometallics are catalytically active in ligand-assisted processes.^22−25^

A step forward in catalysts design that has provided superior catalytic systems in hydrogenation and dehydrogenation reactions relies on the use of ligands containing two Brønsted acidic/basic sites, such as a lutidine fragment and a secondary amino group.^25,26^ Since two Brønsted functionalities are present in these ligands, the related complexes might participate in ligand-assisted processes through pyridine aromatization/dearomatization or amine-metal/amido-metal interconversion. Herein, we report on the synthesis of a series of Ir complexes based on lutidine-derived CNN^H^ ligands bearing secondary amino and NHC side-arms and their catalytic performance in AB dehydrogenation. Detailed kinetic and NMR spectroscopy studies show that different species are formed under catalytic conditions, depending on the substituent of the secondary amino group. Moreover, DFT calculations have determined the preferred metal–ligand cooperation mode when a doubly deprotonated catalytic species is formed.

## Results and Discussion

### Synthesis and Structure of Ir-CNN^H^ Complexes

**Scheme 1:**
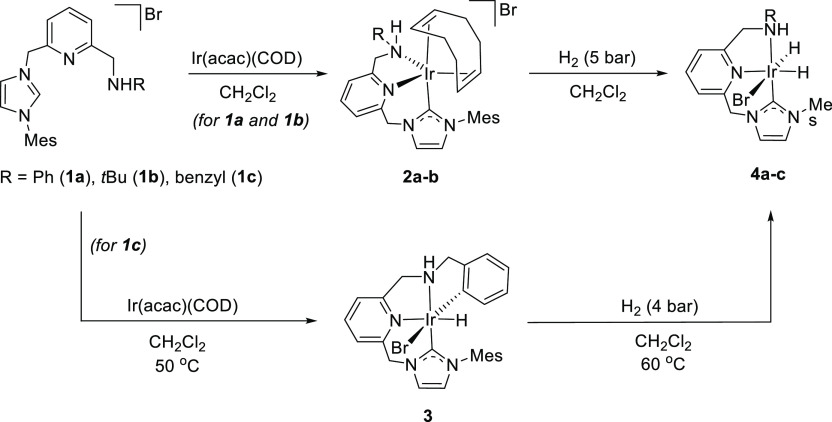
Synthesis of the Ir-CNN^H^ Complexes **2a**, **2b**, **3**, and **4a**–**4c**

Following previously reported procedures for the synthesis of Ir complexes based on related NHC-containing lutidine-derived ligands,^23,24^ the diolefin derivatives **2a** and **2b** were prepared by the reactions of Ir(acac)(COD) and the N-heterocyclic carbene ligand precursors **1a** and **1b**,^25^ respectively (Scheme 1). These complexes were isolated as yellow solids in good yields (99% and 77%, respectively), and spectroscopical and analytically characterized. In the ^1^H NMR spectrum of **2a**, the olefinic hydrogens of one of the C=C moieties of the COD ligand appear as multiplets at δ 4.51 and 4.41 ppm, whereas the resonances corresponding to the other olefin fragment are shown at δ 2.95 and 2.82 ppm. Moreover, the ^1^H–^1^H EXSY experiment of **2a** recorded at 298 K exhibits intense cross-peaks corresponding to the pairwise exchange of the olefinic signals corresponding to different C=C moieties. The observed dynamic behavior can be assigned to alkene site exchange involving the decoordination of one of the olefin fragments to produce a distorted tetrahedral intermediate, and subsequent recoordination of the uncoordinated C=C group to the opposite side (see the Supporting Information).^23^ Meanwhile, the C-2 of the NHC ligand fragment produces a singlet at δ_C_ 181.0 ppm in the ^**13**^C{^1^H} NMR spectrum. Similar NMR data and fluxional behavior were shown by complex **2b**.

**Figure 2:**
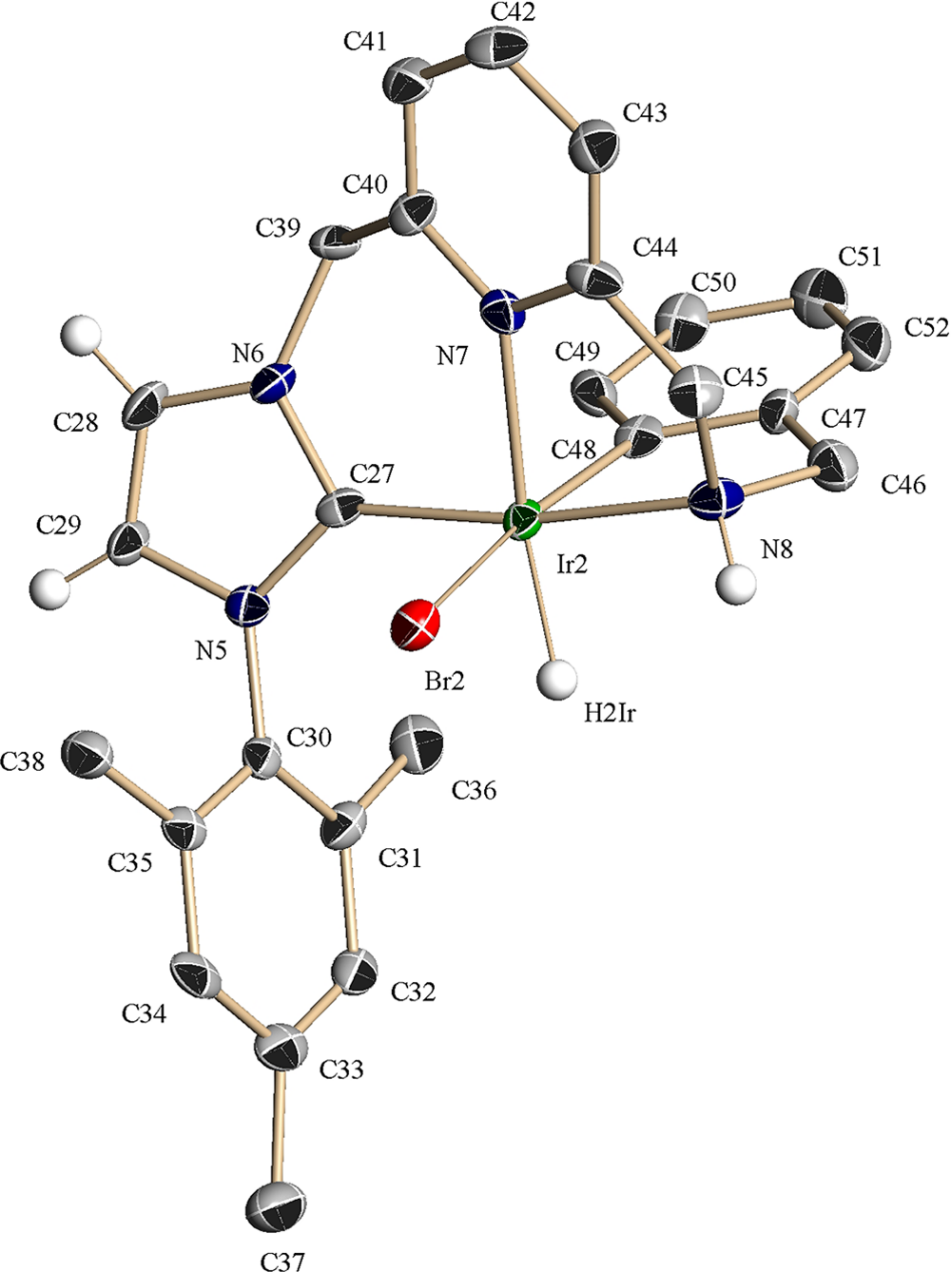
ORTEP drawing at 30% ellipsoid probability of complex **3**. Hydrogen atoms (except for the hydride ligand and the NHC and NH hydrogens) have been omitted for clarity. Selected bond lengths: Ir(2)–C(27), 1.959(10) Å; Ir(2)–N(7), 2.124(7) Å; Ir(2)–N(8), 2.132(8) Å; Ir(2)–Br(2), 2.6266(11) Å; and Ir(2)–C(48), 2.010(10) Å. Selected bond angles: C(27)–Ir(2)–N(8), 171.0(3)°; C(27)–Ir(2)–C(48), 97.4(4)°; C(27)–Ir(2)–N(7), 91.4(3)°; N(8)–Ir(2)–N(7), 79.7(3)°; and C(48)–Ir(2)–Br(2), 169.1(3)°.

Conversely, heating a CH_2_Cl_2_ solution of Ir(acac)(COD) and the imidazolium salt **1c** to 50 °C produced the expected coordination of the lutidine-derived CNN^H^ pincer ligand along with the activation of one *ortho* C–H bond of the benzyl substituent, yielding the κ^4^-(C^NHC^,N^Py^,N^amine^,C^aryl^) complex **3** (Scheme 1). The solid-state structure of this derivative was determined by single-crystal X-ray diffraction (Figure 2). The complex is comprised of a stereogenic Ir center in an octahedral coordination geometry, in which the carbene carbon and the nitrogen donors of the CNN^H^ ligand are coordinated in the meridional positions, as defined by the C_NHC_–Ir-N_amine_ angle of 171.0°, and the metalated aryl fragment is located *trans* to the Br atom (C_aryl_–Ir-Br = 169.1°).

Complexes **2a** and **2b** reacted with H_2_ (5 bar) in CH_2_Cl_2_, resulting in the formation of the bromodihydride complexes **4a** and **4b**, respectively (Scheme 1). Similarly, derivative **4c** was obtained by exposing a CH_2_Cl_2_ solution of **3** at 60 °C to 4 bar of H_2_. Complexes **4a**–**4c**, which were isolated as air-stable yellow solids, were characterized analytically and spectroscopically. For example, in the ^1^H NMR spectrum of complex **4a**, two mutually coupled doublets are detected in the hydride region, appearing at −19.05 and −23.25 ppm (^2^*J*_HH_ = 6.9 Hz), which are assigned to the IrH hydrogens located *trans* and *cis* to the pyridine fragment, respectively, as determined by ^1^H–^1^H NOESY spectroscopy. In the same experiment, the NH hydrogen produces a broad doublet resonance at 6.15 ppm (^3^*J*_HH_ = 11.1 Hz). In the ^13^C{^1^H} NMR spectrum, diagnostic signals for complex **4a** are shown at 153.5 ppm, caused by the carbene carbon, and at 62.2 and 55.3 ppm, corresponding to the methylene NH- and NHC-arms of the pincer, respectively. Analogous NMR data were observed in the case of complexes **4b** and **4c**, with the notable exception of the resonances due to the NH groups in the ^1^H NMR spectra, which are shifted upfield, with respect to that of **4a**, appearing as a broad doublet of doublets at 4.03 ppm (^3^*J*_HH_ = 12.5 Hz, ^3^*J*_HH_ = 3.2 Hz) for **4b**, and 4.39 ppm (^3^*J*_HH_ = 9.2 Hz, ^3^*J*_HH_ = 9.2 Hz) in the case of **4c**.

**Figure 3:**
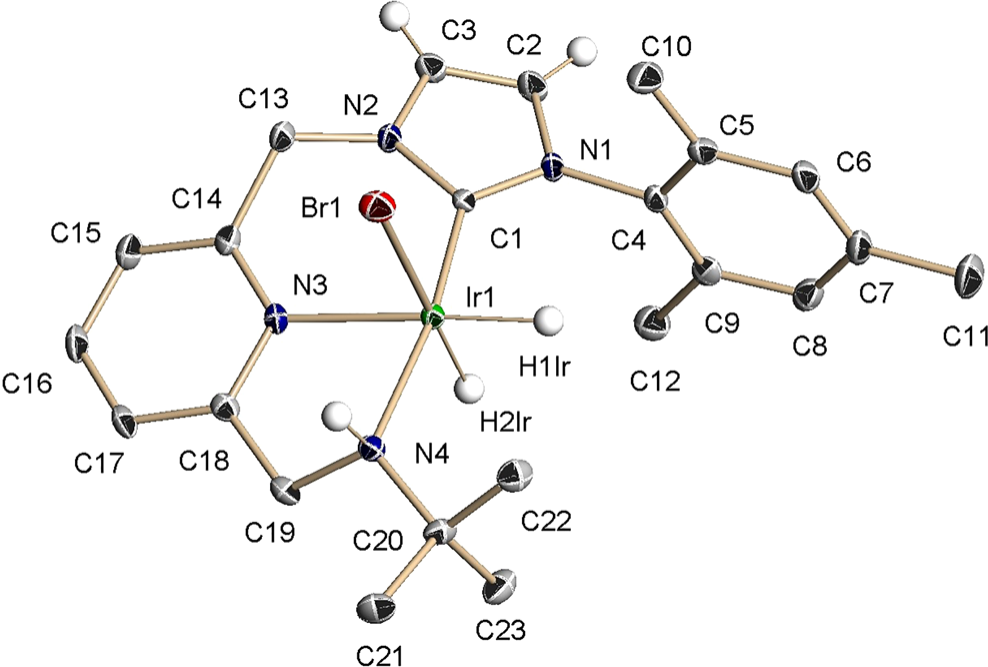
ORTEP drawing at 30% ellipsoid probability of complex **4b**. Hydrogen atoms (except for the hydride ligands and the NHC and NH hydrogens) have been omitted for clarity. Selected bond lengths: Ir(1)–C(1), 1.939(3) Å; Ir(1)–N(3), 2.116(3) Å; Ir(1)–N(4), 2.184(3) Å; Ir(1)–Br(1), 2.6385(4) Å; Ir(1)-H(1Ir), 1.569(18) Å; and Ir(1)–H(2Ir), 1.591(18) Å. Selected bond angles: C(1)–Ir(1)–N(4), 169.62(12)°; C(1)–Ir(1)–N(3), 91.83(12)°; N(3)–Ir(1)–Br(1), 88.86(8)°; and N(4)–Ir(1)–N(3), 78.04(10).

The solid-state structure of **4b**, as determined by single-crystal X-ray diffraction, exhibits an octahedral geometry with the CNN^H^ pincer adopting a meridional coordination and the two hydride ligands in *cis* to each other (Figure 3). Moreover, the existence of a hydrogen bond between the bromine ligand and the amino hydrogen is evident from the H**···**Br distance of 2.76 Å, which is shorter than the sum of the van der Waals radii of H and Br (2.97 Å).^27^

### AB Dehydrogenation

**Figure 4:**
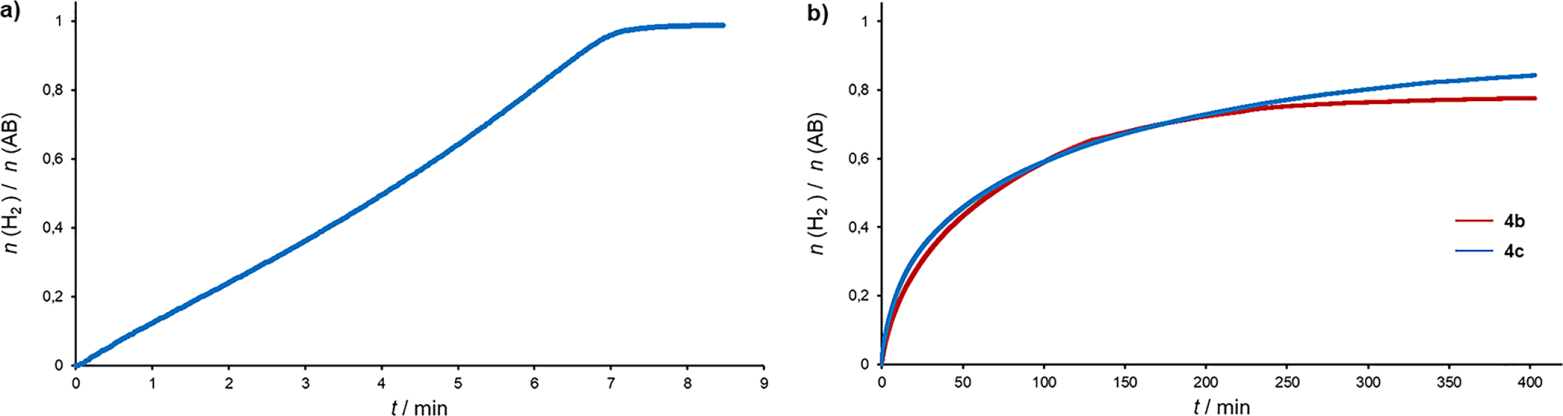
H_2_ evolution in the catalytic dehydrogenation of AB with (a) **4a** (0.4 mol %) and (b) **4b** and **4c** (0.8 mol %). Conditions: THF, room temperature, *t*BuOK:**4** = 2.5, [AB] = 1.6 M.

Complexes **4a**–**4c** were tested as catalysts in the dehydrogenation of ammonia borane after a preliminary activation with *t*BuOK (see the Supporting Information for the experimental setup). The initial treatment with a base is necessary to trigger the catalysis; bare complex **4a** (without a base) was tested in AB dehydrogenation, and it was found to be a poor catalyst (initial turnover frequency (TOF) of <5 h^–1^). Upon addition of a THF solution of **4a** (0.4 mol %) and *t*BuOK (base:**4a** = 2.5) to a stirred AB solution in the same solvent, instantaneous vigorous gas evolution was observed, followed by the immediate formation of an off-white precipitate. Follow-up of the reaction by measuring the pressure increase in the system showed that 1.0 equiv of H_2_ per AB molecule was released in ca. 8.5 min at room temperature (Figure 4a). To the best of our knowledge, the high room-temperature catalytic activity of **4a** (TOF = 1764 h^–1^) is only surpassed by the cationic Pd complexes [Pd(allyl)][BF_4_], [Pd(allyl)(2,4-hexadiene)][BF_4_] and [Pd(MeCN)_4_][BF_4_]_2_ reported by the group of Michalak in 2010,^28^ and by the Ru-PN^H^P and RuCl_2_(PN)_2_ derivatives reported by the Schneider group in 2009^13^ and the Fagnou group in 2008,^17^ respectively. Note that the catalytic activity of **4a** is slightly superior to that of the most active Ir-based catalyst reported to date: IrH_2_(POCOP), published by Goldberg and colleagues in 2006.^7^ Thus, **4a** is currently the best-performing iridium-containing homogeneous catalyst for AB dehydrogenation. On the other hand, reactions performed with complexes **4b** and **4c**, using 0.8 mol % of catalyst loading under the same reaction conditions, yielded ca. 0.75–0.8 equiv of H_2_ in 5 h (for **4b**, TOF_50%_ = 55 h^–1^; for **4c**, TOF_50%_ = 60 h^–1^); thus, both catalysts exhibit significantly lower catalytic activity than **4a** (Figure 4b).

The registered ATR-IR spectrum of the insoluble H_2_-depleted material produced in the AB dehydrogenation reactions with **4a** (amounting to >95% of the total BN byproducts, as estimated by mass balance) contains peaks corresponding to the N–H (3299 and 3248 cm^–1^) and B–H (2384 and 2313 cm^–1^) stretching modes (see Figure S7 in the Supporting Information). These absorptions, as well as the spectrum fingerprint, closely resemble those described for the mainly linear poly(aminoboranes), [NH_2_BH_2_]_*n*_, isolated from the dehydrogenation of AB with Brookhart́s Ir-POCOP complex,^29^ and Ru- and Fe-PN^H^P complexes.^13,14^ The ^11^B NMR spectrum of the soluble part of the reaction with **4a** exhibits broad signals between δ_B_ 17–31 ppm, in agreement with the formation of species resulting from the release of more than 1 equiv of H_2_ (see Figure S8 in the Supporting Information). However, the small fraction of these products is not enough to account for an overall H_2_ yield exceeding 1.0 equiv.

The observed differences in the catalytic activity provided by **4a** and complexes **4b** and **4c** led us to determine the reaction kinetic laws. For **4a**, plotting of [AB]_o_–[AB] versus time gave a straight line (*k*_obs_ = 3.4 × 10^–3^ M s^–1^), indicative of a pseudo-zero order relationship in AB (see Figures S10 and S11 in the Supporting Information). Initial rate experiments performed at different catalyst concentrations showed that hydrogen release has a first-order rate dependence in **4a** (Figure S12 in the Supporting Information). The observed lack of dependence on AB concentration is in agreement with its rapid reaction with the catalyst and, therefore, with a rate-determining step that does not involve AB activation.

Next, kinetic isotope effects (KIE) values were determined using the deuterated AB isotopologues (NH_3_BD_3_, ND_3_BH_3_, and ND_3_BD_3_). A pronounced decrease in the reaction rate was observed with the N-deuterated substrate [ND_3_BH_3_, *k*_H_/*k*_D_ = 8.4], while deuteration at boron produces a somewhat lower rate [NH_3_BD_3_, *k*_H_/*k*_D_ = 1.4]. In the case of full AB deuteration, within the experimental error, the KIE is the product of the individual isotope effects measured with ND_3_BH_3_ and NH_3_BD_3_ [ND_3_BD_3_, *k*_H_/*k*_D_ = 13.4]. This suggests a concerted, asynchronous transition state in the cleavage of the N–H and B–H bonds. However, this conclusion is tentative, since the observed KIEs should reflect the contributions of all the multiple steps of the catalytic cycle. We must bear in mind that AB activation does *not* seem to be the rate-determining step.^30^

The kinetic law was also inferred for the reaction with the catalyst precursor **4b**. From the initial rate experiments performed at different AB and catalyst concentrations, a kinetic lawd[AB]/d*t* = *k*_obs_[AB]^2^ = *k*[**4b**][AB]^2^ (*k*_obs_ = 1.3 × 10^–4^ M^–1^ s^–1^) was revealed, evidencing different rate laws for the reactions with complexes **4a** and **4b** (see Figures S14 and S15 in the Supporting Information). For the latter catalyst, the KIE values using deuterated NH_3_BD_3_, ND_3_BH_3_, and ND_3_BD_3_ were found to be 2.5, 2.8, and 7.7, respectively. These data suggest a mechanism where two AB molecules are involved in the rate-limiting step,^12^ with the N–H and B–H bonds splitting occurring in a concerted manner.

### Mechanistic Insights

**Scheme 2:**
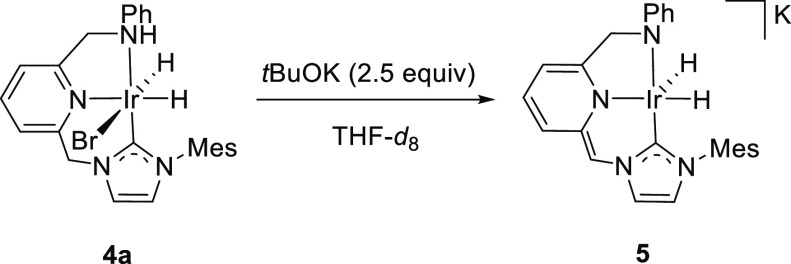
Formation of Iridate Complex **5**

Taking into account previous studies with catalysts based on proton-responsive ligands,^13,14,16,17^ a plausible mechanism for AB dehydrogenation catalyzed by complexes **4** in the presence of an auxiliary base should involve initial hydrogen transfer from AB to the deprotonated form of the Ir-CNN^H^ catalyst precursors with the concomitant formation of the “inorganic ethylene analogue” BH_2_=NH_2_, followed by ligand-assisted H_2_ elimination. Therefore, to gain insight into the species formed upon treatment of complexes **4** with a base, derivative **4a** was initially reacted with *t*BuOK (2.5 equiv) in THF-*d*_8_ (Scheme 2). Reaction of the Ir-CNN^H^ derivatives with a strong base could induce deprotonation of the pincer ligand at its methylene carbon in the side arm^22−25^ and/or at the secondary amino group.^20,25,31^ Analysis of the deuterated solution through NMR spectroscopy showed the formation of a major species (ca. 90%), which was characterized as the highly air-sensitive amido iridate(III) **5**,^32^ confirming the deprotonation of both the NH and the NHC–methylene arm of **4a**. The ^1^H NMR spectrum of **5** features two mutually coupled doublets at δ −16.35 ppm and δ −18.61 ppm (^2^*J*_HH_ = 5.5 Hz), corresponding to the hydride ligands. In the same experiment, the methylene CH_2_NPh hydrogens appear as doublets at δ 4.24 and 3.80 ppm (^2^*J*_HH_ = 16.5 Hz), while the methine CH-NHC hydrogen is a singlet at δ 6.25 ppm. In the ^13^C{^1^H} NMR spectrum, the carbene carbon produces a singlet at δ_C_ 156.8 ppm, whereas the CH_2_N and CH-NHC bridges of the pincer give rise to resonances at δ_C_ 64.2 and 49.0 ppm, respectively.

**Figure 5:**
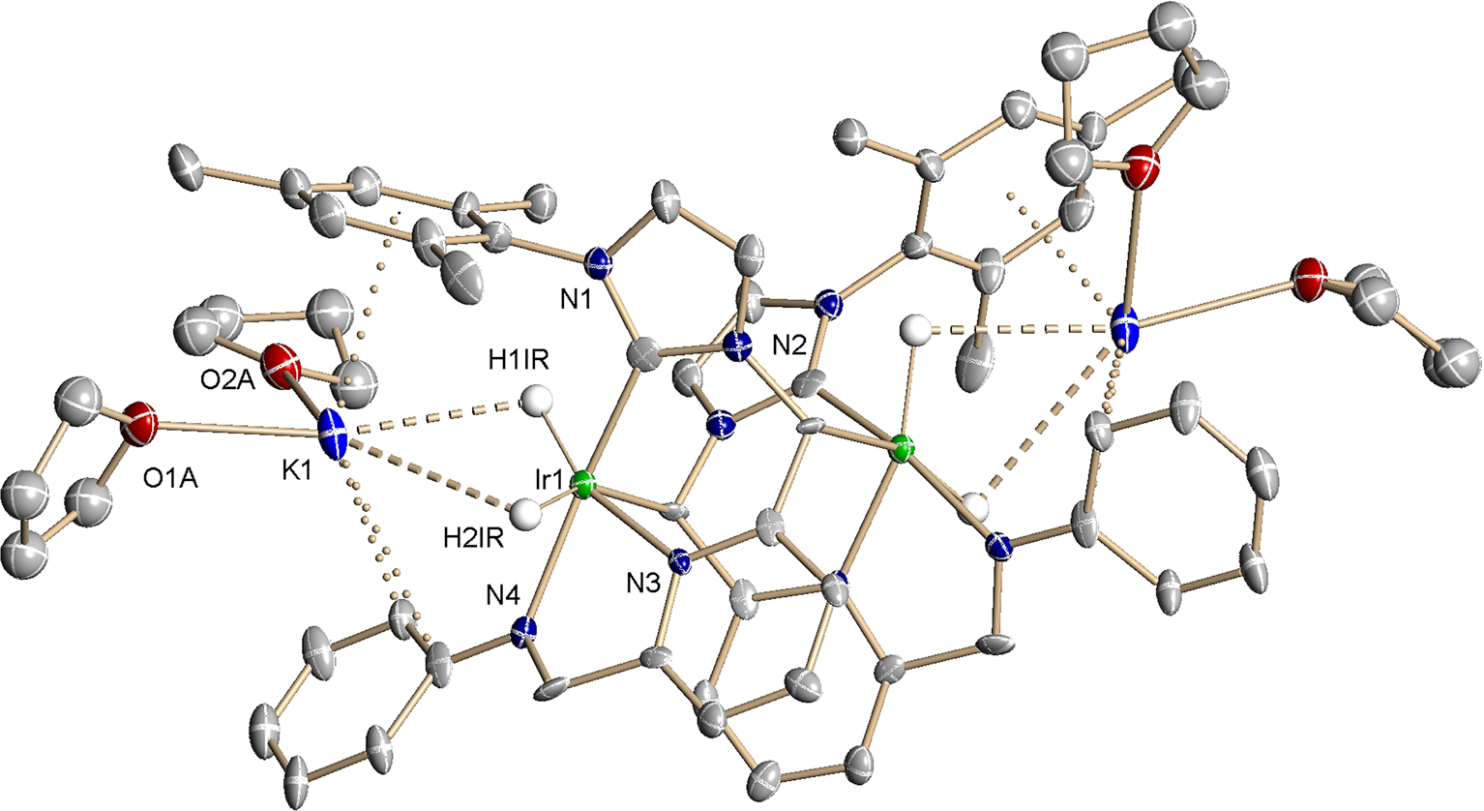
ORTEP drawing at 30% ellipsoid probability of the dimeric form of complex **5**. Hydrogen atoms (except for the hydride ligands) have been omitted for clarity. Selected bond lengths: Ir(1)–C(1), 1.999(19) Å; Ir(1)–N(3), 2.033(13) Å; Ir(1)–N(4), 2.115(15) Å; and Ir(1)–C(13), 2.215(17) Å. Selected bond angles: C(1)–Ir(1)–N(4), 165.9(7)°; C(1)–Ir(1)–N(3), 90.4(7)°; N(3)–Ir(1)–N(4), 78.5(6)°; C(1)–Ir(1)–C(13), 98.9(6)°; and N(4)–Ir(1)–C(13), 90.6(6)°.

Crystals suitable for X-ray diffraction (XRD) analysis of **5** were obtained by cooling the above THF-*d*_8_ solution to −30 °C (Figure 5). The X-ray data confirmed the initially proposed structure. The potassium iridate **5** is dimeric in the solid state, and the two Ir centers are bound to the opposite ligands through the deprotonated CH-NHC arms. The Ir atoms are in an octahedral coordination geometry, with the pincer and one hydride ligand occupying the meridional plane [Σ(Ir) = 361.2°] and the other IrH hydrogen and the Ir(CNN*)H_2_ unit coordinated at the apical positions. The K^+^ counterions are surrounded by two molecules of THF (*d*_(K–O)_ = 2.61 Å, avg.), an η^4^-coordinated mesityl ring (K–C distances of 3.30–3.39 Å), one carbon of the *N*-phenyl ring (*d*_(K–C)_ = 3.14 Å), and the meridional hydride ligand (*d*_(K–H)_ = 2.85 Å). The observed dinuclear solid-state structure of **5** should be favored by the large planarity of the deprotonated Ir-CNN* fragments.

Although in the solid state, complex **5** can be regarded as a dimer, the structure of **5** in solution is that of a monomer, as evidenced by diffusion NMR studies.^33^ The ^1^H DOSY experiment of **5** afforded a diffusion coefficient *D* = 7.2 × 10^–10^ m^2^/s (log *D* = −9.14) from which a value of its hydrodynamic radius (*r*_H_) of 6.4 Å was estimated using the Stokes–Einstein equation. The radius for the dimeric structure calculated from its X-ray volume^34^ was *r*_X-ray_ = 13.9 Å, which is approximately twice the *r*_H_ of **5** in solution. Moreover, the diffusion coefficient *D* of **5** is very similar to that obtained for **4a** using ^1^H DOSY spectroscopy [*D* = 8.2 × 10^–10^ m^2^/s; log *D* = −9.09; *r*_H_ = 5.5 Å]. This further supports the existence of a monomeric form of **5** in solution.

**Scheme 3:**
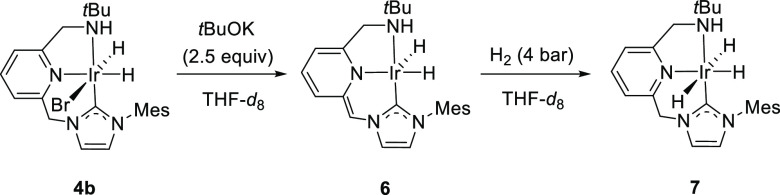
Formation of Deprotonated Complex **6** and Trihydride Derivative **7**

In marked contrast, under the conditions used for the formation of **5**, reaction of **4b** with *t*BuOK (2.5 equiv) in THF-*d*_8_ gave rise to complex **6** (75%–80% NMR yield), which is selectively deprotonated at the NHC-bridge, as determined through NMR spectroscopy (Scheme 3). The ^1^H NMR experiment of **6** shows, in the hydride region, two mutually coupled doublets appearing at −16.49 and −18.41 ppm (^2^*J*_HH_ = 5.6 Hz). Moreover, in the same spectrum, a doublet of doublets at 3.88 ppm (^2^*J*_HH_ = 13.6 Hz, ^3^*J*_HH_ = 2.8 Hz) and a multiplet at 3.39 ppm, corresponding to the CH_2_N hydrogens, and a broad doublet at δ 2.97 ppm (^2^*J*_HH_ = 10.9 Hz) due to the NH, are also observed; whereas, the proton of the deprotonated methine-NHC arm produces a singlet at 5.48 ppm. In the ^13^C{^1^H} NMR spectrum of **6**, the carbene carbon appears as a singlet at 150.0 ppm, while the N- and NHC-linkers of the pincer produce singlet resonances at 56.1 and 54.9 ppm, respectively. Further attempts to perform the deprotonation of the amino group of **4b** were unsuccessful, likely due to the reduced NH acidity, in comparison to **4a**.

Because of the fast kinetics, attempts to detect metallic intermediates through NMR spectroscopy during the dehydrogenation reaction catalyzed by **4a** at room temperature were unsuccessful. However, at the end of the catalytic process, the main metal-containing species present in solution (>90%, from ^1^H NMR analysis) is the amido iridate(III) **5**. Moreover, pressurization of a THF-*d*_8_ solution of **5** with H_2_ (4 bar) did not lead to the formation of new observable species (i.e., a trihydride Ir complex).

For **4b**, the solution composition at the end of the dehydrogenation process, performed at the NMR scale in THF-*d*_8_, is more complex than that found for **4a**: the ^1^H NMR spectrum of the reaction mixture reveals the presence of NHC-deprotonated iridium dihydride **6** as the major component (ca. 30%), together with at least four other unidentified Ir hydrides. In addition, reaction in THF-*d*_8_ of the in situ formed complex **6** with H_2_ (4 bar, 48 h) produced the selective formation of trihydride derivative **7**, which is only stable under a hydrogen atmosphere (Scheme 3). Complex **7** gives rise in the hydride region of the ^1^H NMR spectrum to a multiplet signal integrating for 2H at −8.92 ppm, corresponding to the apical IrH hydrogens, and a doublet of doublets at −18.24 ppm (^2^*J*_HH_ = 5.2, 5.2 Hz) that integrates for 1H, ascribed to the meridional hydride ligand. Moreover, the reprotonation of the pincer ligand side arm was confirmed from the presence of two mutually coupled doublets appearing at 5.02 and 5.10 ppm (^2^*J*_HH_ = 13.5 Hz), corresponding to the methylene–NHC pincer arm.

### DFT Calculations of the First AB Dehydrogenation Step

**Figure 6:**
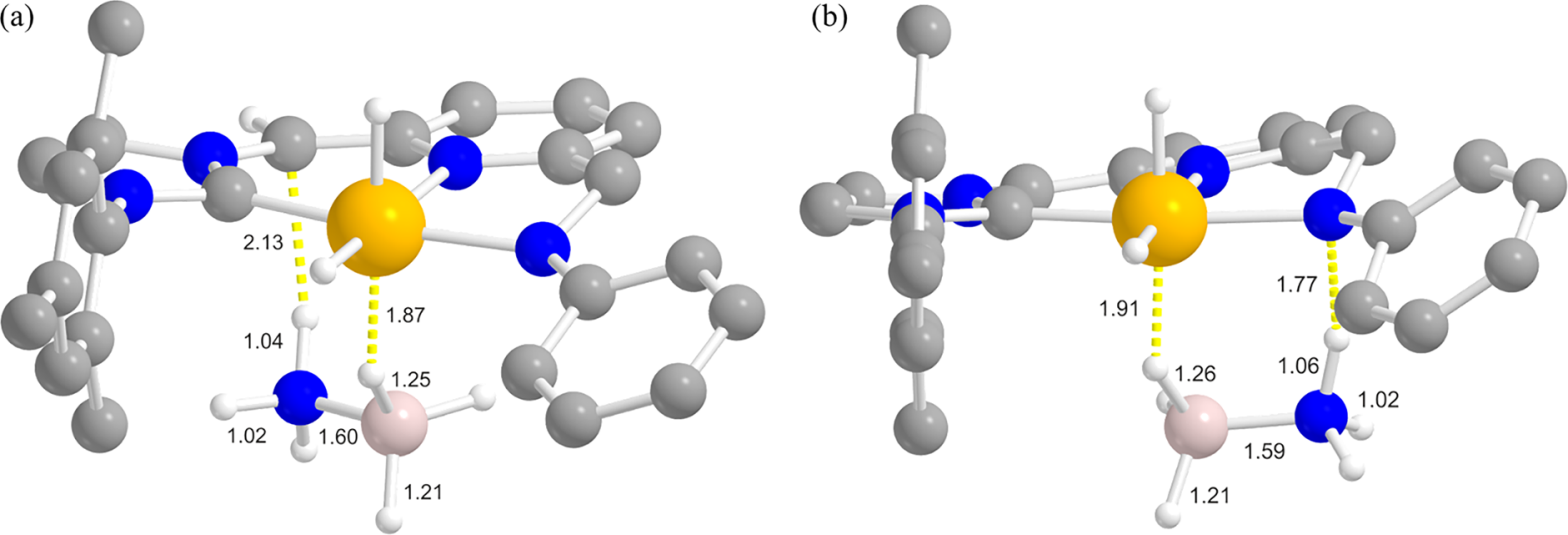
Optimized structure of (CNN*)IrH_2_(η^1^-*BH*-AB) with (a) NH_3_ interaction with the carbanionic C atom (**Ia**) and (b) with NH_3_ interaction with the amido N atom (**Ib**). Selected optimized bond lengths are reported in Ångstroms. H atoms on the pincer ligand not relevant for the discussion are omitted for clarity. Atom color code: white, H; gray, C; blue, N; pink, B; orange, Ir.

To get further insight into the reaction catalyzed by **4a**, DFT calculations on the first step of the AB dehydrogenation mechanism were performed at the DFT//MN15 level of theory on the real structure of the supposed active catalytic species **5**, dimeric in the solid state (as found in the crystal structure determined from X-ray data collection) but monomeric in THF solution (as confirmed by DOSY NMR experiments). In this structure, possibly in the form of a THF adduct with general formula [(CNN*)IrH_2_(thf)]^−^, the solvent O atom occupies the empty coordination site *trans* to one of the hydride ligands, with the Ir^III^ ion in an octahedral coordination geometry. Successive THF/AB exchange in the metal coordination sphere is thermodynamically favored, leading to the complex [(CNN*)IrH_2_(AB)]^−^ (**I**, Figure 6). For this complex, two different isomeric forms are conceivable, depending on the interaction site of AB NH_3_ end. While the BH_3_ end is always directly bound to the metal center in a η^1^-fashion, the NH_3_ end may be interacting either with the carbanionic C atom on the pincer skeleton (“C-path”) or with the negative N atom of the NPh^–^ amido side arm (“N-path”). In principle, both sites are nucleophilic and both interactions are possible. No interaction with the central pyridine N atom or with the carbene arm was found. This is the typical example of “bifunctional catalyst” bearing an acidic (Ir^III^) and a basic (CH^–^/NPh^–^) reactive site at the same time, where the metal center and the pincer ligand act cooperatively to extract hydrogen from AB. This class of compound is widespread in the literature, and it has been frequently exploited in the dehydrogenation of BN lightweight inorganic hydrides.^13,14,16−19^ The analysis of the relative stability of the two isomers **Ia** (HC**···**H–N interaction, Figure 6a) and **Ib** (PhN**···**H–N interaction, Figure 6b) revealed that the latter is slightly more stable [Δ*G*_THF_(**Ia**/**Ib**) = −2.9 kcal/mol]. Given the small Gibbs energy difference between the two isomers, both forms were taken into account for the calculation of the reaction profiles. Consistent with the kinetic data from the KIE experiments, a simultaneous BH/NH bond activation occurs and the B–H bond activation is less difficult than that of the N–H bond, since AB coordination to the metal ion through its BH_3_ end is responsible for a B–H bond “preliminary” weakening. Indeed, in **I**, the B–H bond is already partially broken, as witnessed by the longer B–H distance on the coordinated bond if compared with the other two.

**Figure 7:**
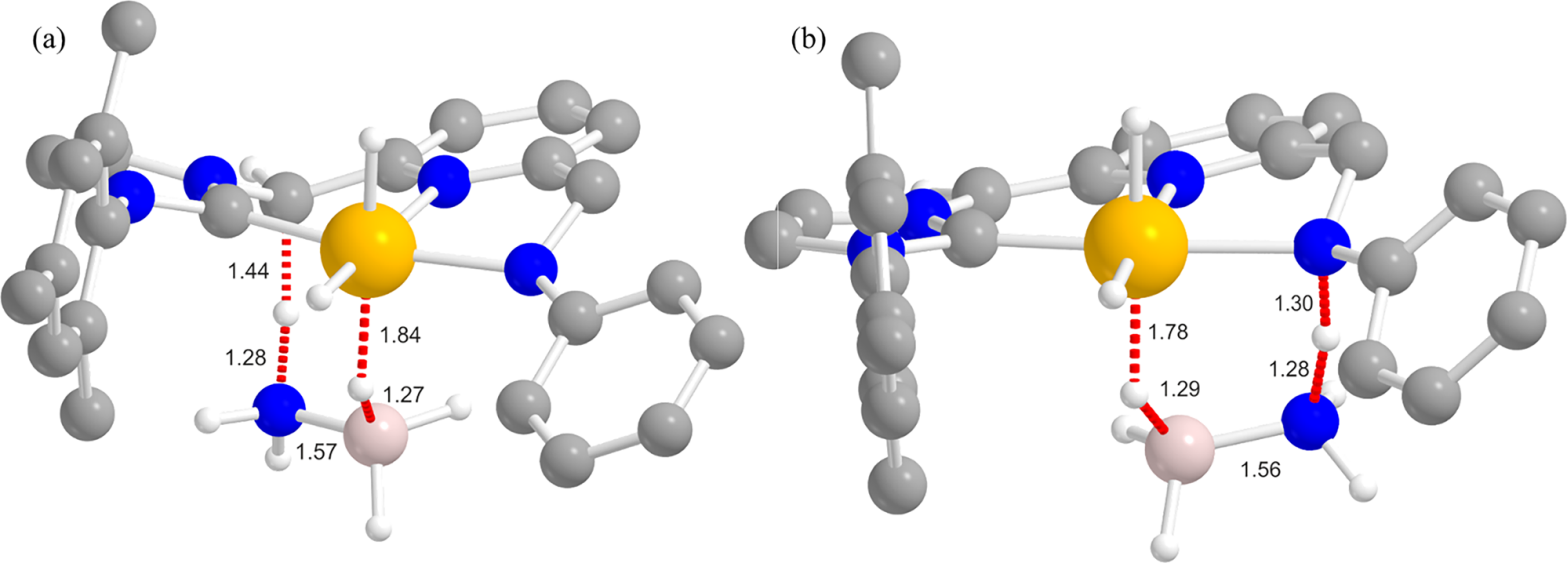
Optimized structures of (a) **TS**_**1C**_ (from **Ia**) and (b) **TS**_**1N**_ (from **Ib**). Selected optimized bond lengths are reported in Ångstroms. H atoms on the pincer ligand not relevant for the discussion are omitted for clarity. Bonds involved in the TS transformation depicted as red dotted lines. See Figure 6 for the atom color code.

From this initial geometry, a transition state **TS**_**1C**_/**TS**_**1N**_ of the CH^–^/NPh^–^ protonation by NH_3_ could be found at Δ*G*_THF_^#^ = 3.9/5.9 kcal/mol along the d(N–H**···**CH)/d(N–H**···**NPh) reaction coordinate (Figure 7), with related “inorganic ethylene analogue” (BH_2_=NH_2_) evolution and formation of the trihydride complex [(CNN)IrH_3_]^−^ (**II**). Thermodynamics for this step is favorable in both cases, with Δ*G*_THF_ = −9.5 and −2.4 kcal/mol for CH^–^ and NPh^–^ protonation, respectively.

**Figure 8:**
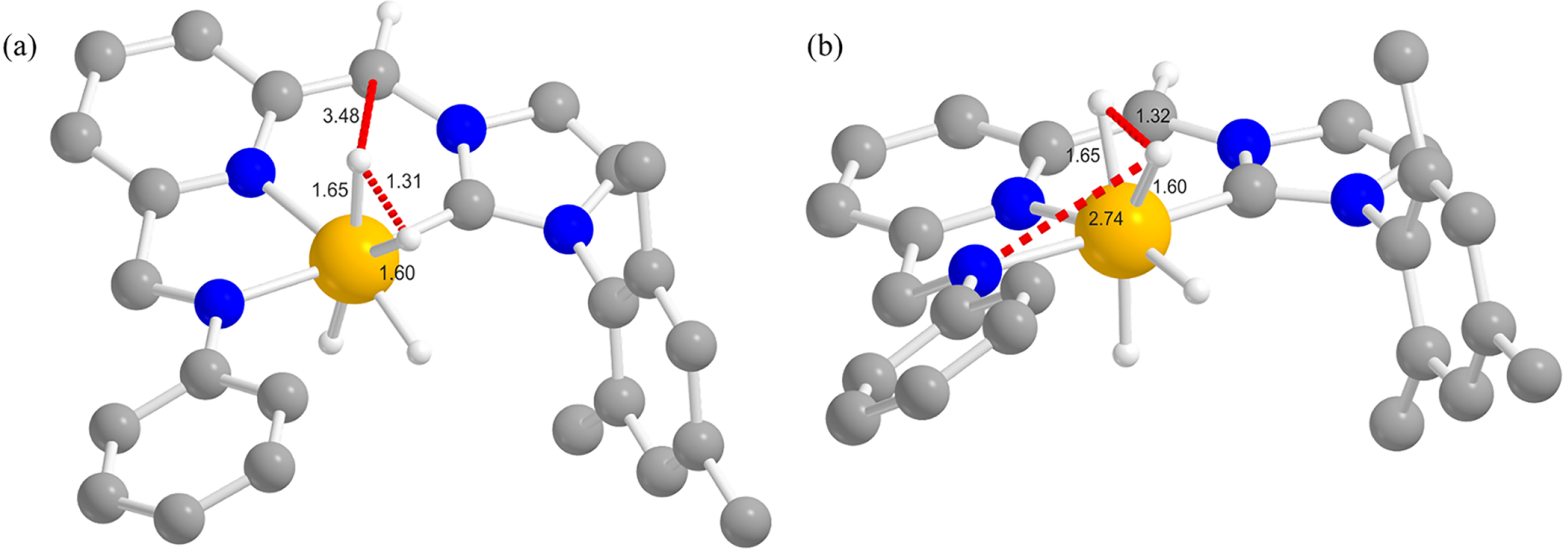
Optimized structures of (a) **TS**_**2C**_ (from **IIa**) and (b) **TS**_**2N**_ (from **IIb**). Selected optimized bond lengths are reported in Ångstroms. H atoms on the pincer ligand not relevant for the discussion are omitted for clarity. Bonds involved in the TS transformation depicted in red dotted lines. See Figure 6 for the atom color code.

**Figure 9:**
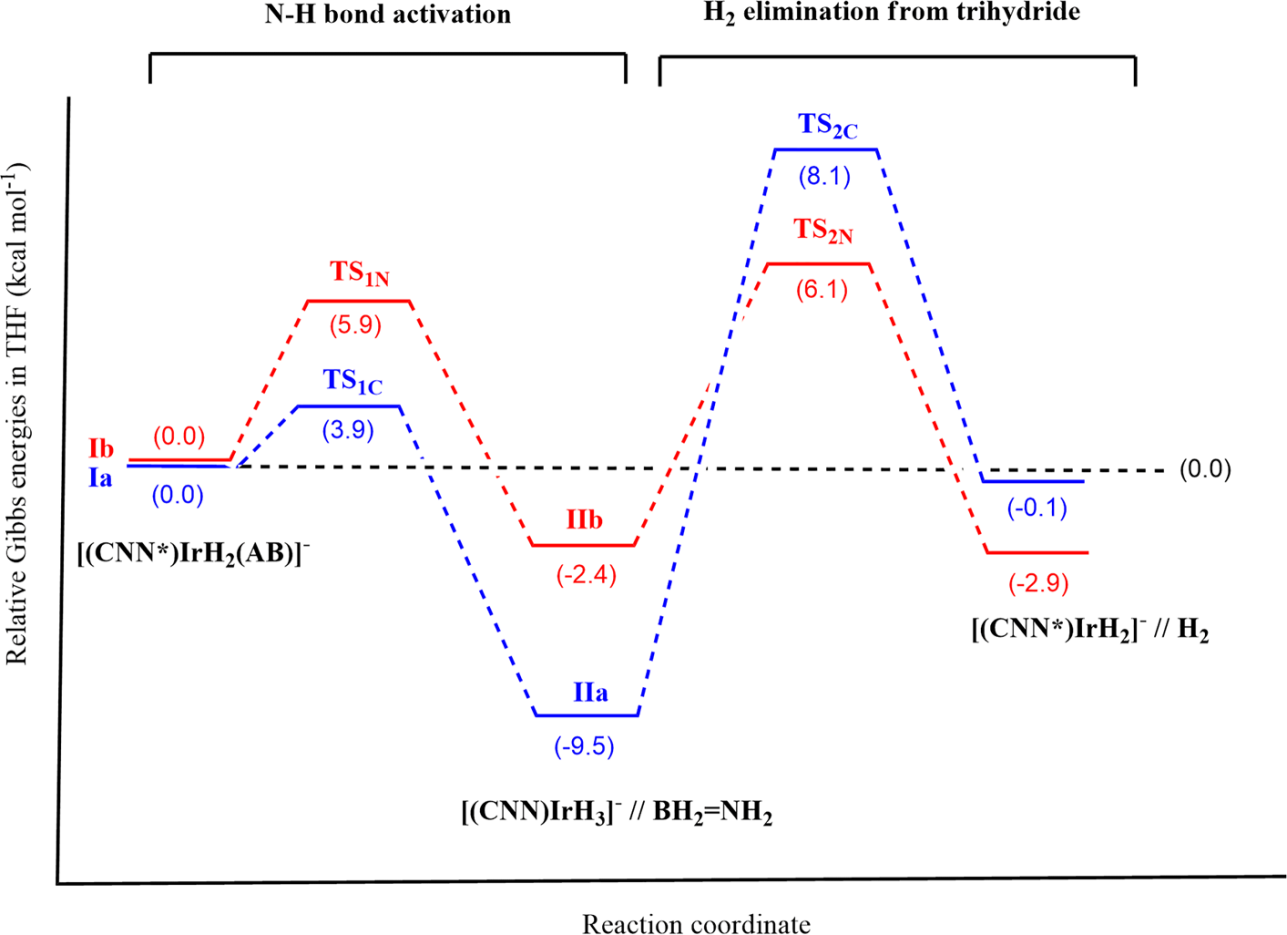
Gibbs energy (THF) vs. reaction coordinate profiles for the first AB dehydrogenation step mediated by **Ia** (blue line) and **Ib** (red line).

From **II** (again, in two isomeric forms **IIa** and **IIb**, depending on the protonated group of choice), different reaction paths were then considered. No H_2_ formation (nonclassical hydride) from the (classical) hydride ligands with concomitant Ir^III^ → Ir^I^ reduction could be observed, at the computational level used in this study. The same can be said for a direct interaction of the hydride ligands of **II** with BH_2_=NH_2_; all the plausible geometries built *in silico* led to repulsive interactions and no maxima could be located on the corresponding reaction energy scan. The only mechanism featured by the presence of a maximum along the examined reaction coordinate is the direct H_2_ elimination from one hydride ligand on iridium and from the methylenic −CH_2_– side arm (**IIa**) or from the NHPh group (**IIb**), to regenerate the starting active species **5** and close the catalytic cycle. The corresponding transition states **TS**_**2C**_ and **TS**_**2N**_ are located at 17.6 and 8.5 kcal/mol above **IIa** and **IIb**, respectively (Figure 8). The overall *G* versus reaction coordinate profiles at comparison are reported in Figure 9. From the inspection of the two profiles, we can conclude that for this complex the “N path” (red trace) is to be preferred to the “C path” (blue trace), from both a kinetic (Δ*G*^#^) and a thermodynamic (Δ*G*) viewpoint. In addition, the rate-determining step in both cases is H_2_ elimination from the trihydride intermediate **II** rather than the easy AB activation by the bifunctional complex **I**. This result is consistent with the zero-order dependence of the reaction rate from AB concentration found experimentally, since AB is not involved in the rate-determining step.

As for **4b**, the adduct of the activated neutral species **6** with two AB molecules is thermodynamically favored: Δ*G*_THF_ for the mono- and bis(AB) adducts equal −1.4 and −0.7 kcal/mol, respectively. The result is in agreement with the experimental evidence of a second-order kinetic dependence from AB concentration. This interaction leads to the geometry **III** (Figure S49), where the first AB molecule sits in the empty coordination site of the five-coordinated **6** interacting through its BH and NH ends with iridium and the deprotonated side arm, respectively [optimized d(Ir**···**H–B) = 1.88 Å; optimized d(H–C**···**H–N) = 2.21 Å]. The second AB molecule weakly interacts with the two hydrides on the metal center to the opposite side of the Ir(CNN) plane [optimized d(Ir–H_axial_**···**H–B) = 2.86 Å; optimized d(Ir–H_equatorial_**···**H–N) = 1.74 Å]. No interaction between AB and the NH*t*Bu group is observed. Therefore, this part of the complex is not involved in the catalytic process and no “N path” can be conceived, at odds with its phenyl-substituted analogue. Unfortunately, no plausible reaction mechanism involving two AB molecules in the rate-determining step could be found, at the computational level used (see the Supporting Information).

## Conclusions

The results reported herein show that highly active Ir^III^ catalysts have been developed by using the same lutidine-derived CNN^H^ ligand scaffold with NH amino groups of different basicity. The phenyl-substituted amino complex **4a** in particular is the most active iridium-based catalyst in AB dehydrogenation to date, to the best of our knowledge. The excellent catalytic activity of **4a** is attributed to the formation of a doubly deprotonated species (the amido iridate **5)** upon reaction with a 2-fold excess of *t*BuOK under catalytic conditions. Meanwhile, double deprotonation of Ir-CNN^H^ complexes based on *N*-alkyl substituted ligands like **4b** cannot be achieved, likely due to the reduced acidity of their amino group. Finally, density functional theory (DFT) calculations of the mechanisms of the first H_2_ release from AB catalyzed by **5** reveal that, despite the presence of two Brønsted basic sites (the amido fragment and the methine-NHC arm), the most favorable metal–ligand cooperation mode is based on reversible metal-amine/metal-amido interconversion. Given the widespread use of metal complexes containing proton-responsive ligands in many catalytic processes, this approach, based on the use of ligands with two proton-responsive sites, could lead to improved catalytic systems. Further applications in catalysis of metal complexes incorporating more than one Brønsted acidic/basic sites are currently being explored in our laboratories.

## Experimental Section

### General Procedures

All reactions and manipulations were performed under nitrogen or argon, either in a Braun Labmaster 100 glovebox or using standard Schlenk-type techniques. All solvents were distilled under nitrogen with the following desiccants: sodium-benzophenone-ketyl for diethyl ether (Et_2_O) and tetrahydrofuran (THF); sodium for pentane and toluene; CaH_2_ for dichloromethane and acetonitrile. Imidazolium salts **1a**–**1c**^25^ and Ir(acac)(COD)^35^ were prepared as previously described. Deuterated ammonia borane adducts (NH_3_BD_3_, ND_3_BH_3_, ND_3_BD_3_) were synthesized by known methods.^36^ All other reagents were purchased from commercial suppliers and used as received. NMR spectra were obtained on Bruker DRX-400 and AVANCEIII/ASCEND 400R spectrometers. ^11^B{^1^H} NMR shifts were referenced to external BF_3_·Et_2_O, while ^13^C{^1^H} and ^1^H shifts were referenced to the residual signals of deuterated solvents. All data are reported in ppm downfield from Me_4_Si. All NMR measurements were performed at 25 °C, unless otherwise stated. NMR signal assignations were confirmed by 2D NMR spectroscopy (^1^H–^1^H COSY, ^1^H–^1^H NOESY, ^1^H–^13^C HSQC and ^1^H–^13^C HMBC) for all the complexes. Elemental analyses were run by the Analytical Service of the Instituto de Investigaciones Químicas in a Leco TrueSpec CHN elemental analyzer. IR spectra were acquired on a Thermo Scientific Nicolet iS5 iD7 ATR instrument.

### Computational Details

Calculations were performed on the real structures of the Ir^III^ anionic complex **5** and the Ir^III^ neutral complex **6** with the Gaussian16^37^ package at the DFT//MN15^38^ level. Effective core potentials (ECP) and associated SDD basis set^39^ supplemented with *f*-polarization functions (SDD(*f*))^40^ were used to describe the inner electronic shells and the *d* valence electrons of the Ir atom. All the other atoms were described with a 6-31++G(d,p) basis set.^41^ The structures of the reactants and complexes were fully optimized with this basis set without any symmetry restrictions in THF (ε = 7.42), which was introduced within the SMD solvation model.^42^ In these optimizations, individual solvation spheres were placed on the H atoms of AB and on the hydride ligands on Ir. The full geometry optimization was followed by the thermochemistry calculations. The nature of all the stationary points on the potential energy surface was confirmed by vibrational analysis. No scaling factors were applied to the calculated frequencies. The transition-state structures showed only one negative eigenvalue in their diagonalized force constant matrices, and their associated eigenvectors were confirmed to correspond to the motion along the reaction coordinate under consideration using the Intrinsic Reaction Coordinate (IRC) method.^43^

### Synthesis of Ir-CNN^H^ Complexes

#### Complex **2a**

A solution of **1a** (0.105 g, 0.23 mmol) and Ir(acac)(COD) (0.090 g, 0.23 mmol) in CH_2_Cl_2_ (10 mL) was stirred for 2 days at room temperature. Volatiles were removed under reduced pressure, and the residue was successively washed with pentane (3 × 8 mL) and Et_2_O (8 mL), and dried under vacuum. Yellow solid (0.179 g, 99%). Anal. Calcd (%) for C_33_H_38_BrIrN_4_: C 51.96, H 5.02, N 7.34; found: C 51.96, H 4.93, N 7.24. ^1^H NMR (400 MHz, CD_2_Cl_2_): δ 7.76 (t, ^3^*J*_HH_ = 7.6 Hz, 1H, H arom Py), 7.40 (d, ^3^*J*_HH_ = 7.6 Hz, 1H, H arom Py), 7.34 (d, ^3^*J*_HH_ = 7.6 Hz, 1H, H arom Py), 7.26 (d, ^3^*J*_HH_ = 1.7 Hz, 1H, H arom NHC), 7.19 (dd, ^3^*J*_HH_ = 7.3 Hz, ^3^*J*_HH_ = 7.3 Hz, 2H, 2 H arom Ph), 7.08 (s, 1H, H arom Mes), 6.98 (s, 1H, H arom Mes), 6.90 (d, ^3^*J*_HH_ = 1.7 Hz, 1H, H arom NHC), 6.71 (m, 3H, 3H arom Ph), 6.45 (d, ^2^*J*_HH_ = 15.6 Hz, 1H, C*H*H-NHC), 5.58 (d, ^2^*J*_HH_ = 15.6 Hz, 1H, CH*H*–NHC), 4.96 (s, 1H, NH), 4.51 (m, 1H, =CH COD), 4.47 (m, 2H, 2 C*H*H–NH), 4.41 (m, 1H, =CH COD), 2.95 (m, 1H, =CH COD), 2.82 (m, 1H, =CH COD), 2.39 (s, 6H, 2 CH_3_), 1.95 (s, 3H, CH_3_), 1.76 (m, 2H, 2 C*H*H COD), 1.56 (m, 1H, C*H*H COD), 1.38 (m, 4H, 4 C*H*H COD), 1.12 (m, 1H, C*H*H COD). ^13^C{^1^H} NMR (101 MHz, CD_2_Cl_2_): δ 181.0 (C2–NHC), 158.7 (C_q_ arom), 156.5 (C_q_ arom), 148.4 (C_q_ arom), 139.1 (C_q_ arom), 137.7 (CH arom), 136.9 (C_q_ arom), 136.3 (C_q_ arom), 135.1 (C_q_ arom), 129.7 (CH arom), 129.5 (2 CH arom), 128.5 (CH arom), 123.6 (CH arom), 122.3 (CH arom), 121.2 (CH arom), 120.9 (CH arom), 117.7 (CH arom), 113.3 (2 CH arom), 83.0 (=CH COD), 82.6 (=CH COD), 56.6 (CH_2_–NHC), 53.4 (=CH COD), 52.5 (=CH COD), 49.3 (CH_2_NH), 34.3 (CH_2_ COD), 32.6 (CH_2_ COD), 30.0 (CH_2_ COD), 29.3 (CH_2_ COD), 21.2 (CH_3_), 20.3 (CH_3_), 18.1 (CH_3_).

#### Complex **2b**

A solution of **1b** (0.176 g, 0.40 mmol) and Ir(acac)(COD) (0.159 g, 0.40 mmol) in CH_2_Cl_2_ (15 mL) was stirred for 24 h at room temperature. Volatiles were removed under reduced pressure, and the residue was washed with pentane (3 × 10 mL) and dried under vacuum. Yellow solid (0.229 g, 77%). Anal. Calcd (%) for C_31_H_42_BrIrN_4_: C 50.12, H 5.70, N 7.54; found: C 49.92, H 5.78, N 7.44. ^1^H NMR (400 MHz, CD_2_Cl_2_): δ 7.72 (dd, ^3^*J*_HH_ = 7.7 Hz, ^3^*J*_HH_ = 7.7 Hz, 1H, H arom Py), 7.34 (m, 2H, 2 H arom Py), 7.23 (d, ^3^*J*_HH_ = 1.5 Hz, 1H, H arom NHC), 7.07 (s, 1H, H arom Mes), 6.98 (s, 1H, H arom Mes), 6.86 (d, ^3^*J*_HH_ = 1.5 Hz, 1H, H arom NHC), 6.40 (d, ^2^*J*_HH_ = 15.7 Hz, 1H, C*H*H–NHC), 5.51 (d, ^2^*J*_HH_ = 15.7 Hz, 1H, C*H*H–NHC), 4.49 (m, 1H, =CH COD), 4.41 (m, 1H, =CH COD), 3.89 (m, 2H, 2 C*H*HNH), 3.01 (m, 1H, =CH COD), 2.81 (m, 1H, =CH COD), 2.39 (s, 6H, 2 CH_3_), 1.96 (br m, 4H, CH_3_ + NH), 1.79 (m, 3H, 3 C*H*H COD), 1.56 (m, 1H, C*H*H COD), 1.43 (m, 1H, C*H*H COD), 1.32 (m, 2H, 2 C*H*H COD), 1.18 (s, 9H, C(CH_3_)_3_), 1.12 (m, 1H, C*H*H COD). ^13^C{^1^H} NMR (101 MHz, CD_2_Cl_2_): δ 180.9 (C2–NHC), 161.5 (C_q_ arom), 156.1 (C_q_ arom), 139.1 (C_q_ arom), 137.5 (CH arom), 136.9 (C_q_ arom), 136.3 (C_q_ arom), 135.1 (C_q_ arom), 129.7 (CH arom), 128.5 (CH arom), 123.6 (CH arom), 122.2 (CH arom), 121.5 (CH arom), 120.7 (CH arom), 82.9 (=CH COD), 82.5 (=CH COD), 56.7 (CH_2_–NHC), 52.4 (2 =CH COD), 50.7 (*C*(CH_3_)_3_), 48.8 (CH_2_NH), 34.3 (CH_2_ COD), 32.6 (CH_2_ COD), 30.1 (CH_2_ COD), 29.3 (C(*C*H_3_)_3_ + CH_2_ COD), 21.2 (CH_3_), 20.3 (CH_3_), 18.1 (CH_3_).

#### Complex **3**

A solution of Ir(acac)(COD) (0.059 g, 0.15 mmol) in CH_2_Cl_2_ (10 mL) was added to a solution of **1c** (0.070 g, 0.15 mmol) in CH_2_Cl_2_ (10 mL). The mixture was stirred at room temperature for 24 h, and at 50 °C for 36 h. Solvent was removed under reduced pressure, and the residue was washed with MeCN (2 × 7 mL) and dried under vacuum. Yellow solid (0.024 g, 24%). Anal. Calcd (%) for C_26_H_28_BrIrN_4_: C 46.70, H 4.22, N 8.38; found: C 46.80, H 4.53, N 8.81. ^1^H NMR (400 MHz, CD_2_Cl_2_): δ 7.68 (dd, ^3^*J*_HH_ = 7.9 Hz, ^3^*J*_HH_ = 7.9 Hz, 1H, H arom Py), 7.30 (d, ^3^*J*_HH_ = 7.9 Hz, 1H, H arom Py), 7.28 (d, ^3^*J*_HH_ = 2.2 Hz, 1H, H arom NHC), 7.15 (d, ^3^*J*_HH_ = 7.9 Hz, 1H, H arom Py), 6.99 (s, 1H, H arom Mes), 6.98 (d, ^3^*J*_HH_ = 2.2 Hz, 1H, H arom NHC), 6.90 (s, 1H, H arom Mes), 6.79 (d, ^3^*J*_HH_ = 7.7 Hz, 1H, H arom Ph), 6.72 (dd, ^3^*J*_HH_ = 7.7 Hz, ^4^*J*_HH_ = 1.2 Hz, 1H, H arom Ph), 6.61 (ddd, ^3^*J*_HH_ = 7.3 Hz, ^3^*J*_HH_ = 7.3 Hz, ^4^*J*_HH_ = 1.2 Hz, 1H, H arom Ph), 6.53 (dd, ^3^*J*_HH_ = 7.3 Hz, ^3^*J*_HH_ = 7.3 Hz, 1H, H arom Ph), 5.78 (d, ^2^*J*_HH_ = 16.0 Hz, 1H, C*H*H–NHC), 5.41 (d, ^2^*J*_HH_ = 16.1 Hz, 1H, C*H*H–NHC), 5.26 (m, 2H, C*H*HNH + NH), 4.50 (dd, ^2^*J*_HH_ = 15.3 Hz, ^3^*J*_HH_ = 7.1 Hz, 1H, C*H*HN), 4.15 (d, ^2^*J*_HH_ = 16.1 Hz, 1H, C*H*HNH), 3.92 (d, ^2^*J*_HH_ = 15.3 Hz, 1H, C*H*HNH), 2.35 (s, 3H, CH_3_), 2.30 (s, 3H, CH_3_), 2.10 (s, 3H, CH_3_), −17.73 (s, 1H, IrH). ^13^C{^1^H} NMR (101 MHz, CD_2_Cl_2_): δ 162.8 (C_q_ arom), 161.3 (C2–NHC), 150.8 (C_q_arom), 148.2 (C_q_arom), 140.0 (Ir–C_q_arom), 138.2 (C_q_arom), 138.1 (C_q_arom), 137.7 (CHarom), 137.1 (C_q_arom), 136.5 (CHarom), 136.4 (C_q_arom), 129.4 (CH arom), 128.6 (CH arom), 124.9 (CH arom), 121.8 (CH arom), 121.7 (CH arom), 120.9 (CH arom), 120.9 (CH arom), 120.7 (CH arom), 120.2 (CH arom), 66.5 (CH_2_NH), 64.2 (CH_2_NH), 55.3 (CH_2_–NHC), 21.3 (CH_3_), 20.1 (CH_3_), 19.7 (CH_3_).

#### Complex **4a**

In a Fisher–Porter vessel, a solution of **2a** (0.172 g, 0.22 mmol) in CH_2_Cl_2_ (8 mL) was pressurized with 5 bar of H_2_ and stirred for 24 h at room temperature. The system was depressurized, solvent was evaporated, and the residue was washed with pentane (2 × 8 mL) cooled to −30 °C and dried. Yellow solid (0.043 g, 29%). Anal. Calcd (%) for C_25_H_28_BrIrN_4_: C, 45.73; H, 4.30; N, 8.53; found: C, 45.41; H, 4.47; N, 8.21. ^1^H NMR (400 MHz, CD_2_Cl_2_): δ 7.86 (dd, ^3^*J*_HH_ = 7.7 Hz, ^3^*J*_HH_ = 7.7 Hz, 1H, H arom Py), 7.46 (dd, ^3^*J*_HH_ = 7.2 Hz, ^3^*J*_HH_ = 7.2 Hz, 2H, 2 H arom Ph), 7.33 (m, 4H, 4 H arom), 7.14 (m, 2H, 2 H arom), 6.96 (m, 2H, 2 H arom), 6.72 (d, ^3^*J*_HH_ = 1.8 Hz, 1H, H arom NHC), 6.41 (d, ^2^*J*_HH_ = 15.0 Hz, 1H, C*H*H–NHC), 6.15 (br d, ^3^*J*_HH_ = 11.1 Hz, 1H, NH), 5.05 (d, ^2^*J*_HH_ = 15.0 Hz, 1H, CH*H*–NHC), 4.76 (dd, ^2^*J*_HH_ = 14.5 Hz, ^3^*J*_HH_ = 2.8 Hz, 1H, C*H*H–NH), 4.62 (d, ^2^*J*_HH_ = 14.5 Hz, ^3^*J*_HH_ = 11.1 Hz, 1H, CH*H*–NH), 2.34 (s, 3H, CH_3_), 2.14 (s, 3H, CH_3_), 1.92 (s, 3H, CH_3_), −19.05 (d, ^2^*J*_HH_ = 6.9 Hz, 1H, IrH *trans* to Py), −23.25 (d, ^2^*J*_HH_ = 6.9 Hz, 1H, IrH *cis* to Py). ^13^C{^1^H} NMR (101 MHz, CD_2_Cl_2_): δ 159.8 (C_q_ arom), 153.5 (C2–NHC), 149.5 (C_q_ arom), 138.0 (C_q_ arom), 137.5 (C_q_ arom), 136.5 (C_q_ arom), 135.6 (CH arom), 135.0 (C_q_ arom), 128.9 (2 CH arom + C_q_ arom), 128.7 (CH arom), 128.3 (CH arom), 125.0 (CH arom), 122.8 (CH arom), 120.9 (CH arom), 120.5 (CH arom), 120.1 (2 CH arom), 119.3 (CH arom), 62.2 (CH_2_NH), 55.3 (CH_2_–NHC), 20.9 (CH_3_), 18.8 (CH_3_), 17.9 (CH_3_).

#### Complex **4b**

This complex was prepared as described above for **4a**. Yellow solid (0.084 g, 40%). Anal. Calcd (%) for C_23_H_32_BrIrN_4_: C 43.39, H 5.07, N 8.80; found: C 43.50, H 5.14, N 8.42. ^1^H NMR (400 MHz, CD_2_Cl_2_): δ 7.77 (dd, ^3^*J*_HH_ = 7.7 Hz, ^3^*J*_HH_ = 7.7 Hz, 1H, H arom Py), 7.37 (d, ^3^*J*_HH_ = 7.7 Hz, 1H, H arom Py), 7.36 (d, ^3^*J*_HH_ = 7.7 Hz, 1H, H arom Py), 7.12 (d, ^3^*J*_HH_ = 2.1 Hz, 1H, H arom NHC), 7.03 (s, 1H, H arom Mes), 6.96 (s, 1H, H arom Mes), 6.71 (d, ^3^*J*_HH_ = 2.1 Hz, 1H, H arom NHC), 6.43 (d, ^2^*J*_HH_ = 14.9 Hz, 1H, C*H*H–NHC), 4.98 (d, ^2^*J*_HH_ = 14.9 Hz, 1H, C*H*H–NHC), 4.53 (dd, ^2^*J*_HH_ = 13.5 Hz, ^3^*J*_HH_ = 3.2 Hz, 1H, C*H*HNH), 4.03 (br dd, ^3^*J*_HH_ = 12.5 Hz, ^3^*J*_HH_ = 3.2 Hz, 1H, NH), 3.91 (dd, ^2^*J*_HH_ = 13.5 Hz, ^3^*J*_HH_ = 12.5 Hz, 1H, C*H*HNH), 2.39 (s, 3H, CH_3_), 2.18 (s, 3H, CH_3_), 1.94 (s, 3H, CH_3_), 1.33 (s, 9H, C(CH_3_)_3_), −19.02 (d, 1H, ^2^*J*_HH_ = 7.1 Hz, IrH *trans* to Py), −23.92 (d, 1H, ^2^*J*_HH_ = 7.1 Hz, IrH *cis* to Py). ^13^C{^1^H} NMR (101 MHz, CD_2_Cl_2_): δ 162.4 (C_q_ arom), 156.9 (C2–NHC), 153.7 (C_q_ arom), 138.3 (C_q_ arom), 138.0 (C_q_ arom), 137.0 (C_q_ arom), 135.5 (C_q_ arom), 135.4 (CH arom), 128.9 (CH arom), 128.5 (CH arom), 122.7 (CH arom), 120.9 (CH arom), 120.6 (CH arom), 119.5 (CH arom), 56.3 (CH_2_NH + *C*(CH_3_)_3_), 55.7 (CH_2_–NHC), 28.8 (C(*C*H_3_)_3_), 21.3 (CH_3_), 19.2 (CH_3_), 18.4 (CH_3_).

#### Complex **4c**

In a Fisher–Porter vessel, a suspension of complex **3** (0.040 g, 0.06 mmol) in CH_2_Cl_2_ (5 mL) was pressurized with 4 bar of H_2_, and stirred at 60 °C for 24 h. The system was depressurized, solvent was evaporated and the residue was washed with and pentane (2 × 8 mL), and dried. Yellow solid (0.039 g, 97%). Anal. Calcd (%) for C_26_H_30_BrIrN_4_: C, 46.56; H, 4.51; N, 8.35; found: C, 46.46; H, 4.84; N, 8.17. ^1^H NMR (400 MHz, CD_2_Cl_2_): δ 7.72 (dd, ^3^*J*_HH_ = 7.4 Hz, ^3^*J*_HH_ = 7.4 Hz, 1H, H arom Py), 7.38 (m, 7H, 7 H arom), 7.20 (d, ^3^*J*_HH_ = 7.4 Hz, 1H, H arom Py), 7.16 (s, 1H, H arom), 7.05 (s, 1H, H arom), 7.00 (s, 1H, H arom), 6.35 (d, ^2^*J*_HH_ = 15.0 Hz, 1H, C*H*H–NHC), 5.05 (d, ^2^*J*_HH_ = 15.0 Hz, 1H, C*H*H–NHC), 4.78 (d, ^2^*J*_HH_ = 13.9 Hz, 1H, C*H*HNH), 4.39 (br dd, ^3^*J*_HH_ = 9.2 Hz, ^3^*J*_HH_ = 9.2 Hz, 1H, NH), 4.28 (dd, ^2^*J*_HH_ = 15.0 Hz, ^3^*J*_HH_ = 3.1 Hz, 1H, C*H*HNH), 3.92 (dd, ^2^*J*_HH_ = 13.3 Hz, ^3^*J*_HH_ = 12.9 Hz, 1H, C*H*HNH), 3.73 (dd, ^2^*J*_HH_ = 13.6 Hz, ^3^*J*_HH_ = 12.9 Hz, 1H, C*H*HNH), 2.40 (s, 3H, CH_3_), 2.22 (s, 3H, CH_3_), 1.96 (s, 3H, CH_3_), −18.83 (d, 1H, ^2^*J*_HH_ = 6.9 Hz, IrH *trans* to Py), −23.28 (d, 1H, ^2^*J*_HH_ = 6.9 Hz, IrH *cis* to Py). ^13^C{^1^H} NMR (101 MHz, CD_2_Cl_2_): δ 161.0 (C_q_ arom), 156.8 (C2–NHC), 153.3 (C_q_ arom), 138.1 (C_q_ arom), 137.9 (C_q_ arom), 136.9 (C_q_ arom), 135.2 (CH arom), 135.0 (C_q_ arom), 129.0 (2 CH arom), 128.8 (3 CH arom), 128.2 (CH arom), 128.0 (CH arom), 122.4 (CH arom), 120.7 (CH arom), 120.2 (CH arom), 119.1 (CH arom), 63.9 (CH_2_NH), 60.3 (CH_2_NH), 55.4 (CH_2_–NHC), 20.9 (CH_3_), 18.9 (CH_3_), 18.0 (CH_3_).

#### Complex **5**

In a J. Young-valved NMR tube, a suspension of complex **4a** (0.014 g, 0.02 mmol) in THF-*d*_8_ (0.5 mL) was treated with *t*BuOK (0.005 g, 0.04 mmol). The sample was analyzed by NMR spectroscopy, showing the formation of complex **5** in ca. 90% NMR yield. Crystals of **5** suitable for XRD analysis were obtained by cooling the above solution at −30 °C. ^1^H NMR (400 MHz, THF-*d*_8_): δ 7.15 (d, ^3^*J*_HH_ = 8.6 Hz, 1H, H arom), 6.97 (d, ^3^*J*_HH_ = 5.1 Hz, 2H, 2 H arom), 7.73 (m, 4H, 4 H arom), 6.41 (s, 1H, H arom), 6.25 (s, 1H, CH–NHC), 6.06 (d, ^3^*J*_HH_ = 7.6 Hz, 2H, 2 H arom), 5.75 (m, 2H, 2 H arom), 4.24 (d, ^2^*J*_HH_ = 16.5 Hz, 1H, C*H*H–NPh), 3.80 (d, ^2^*J*_HH_ = 16.5 Hz, 1H, C*H*H–NPh), 2.29 (s, 3H, CH_3_), 2.21 (s, 3H, CH_3_), 1.77 (overlapped with solvent signal, CH_3_), −16.35 (d, ^2^*J*_HH_ = 5.5 Hz, 1H, IrH), −18.61 (d, ^2^*J*_HH_ = 5.5 Hz, 1H, IrH). ^13^C{^1^H} NMR (101 MHz, THF-*d*_8_): δ 164.4 (C_q_ arom), 164.0 (C_q_ arom), 159.0 (C_q_ arom), 156.8 (C2–NHC), 142.3 (C_q_ arom), 138.7 (C_q_ arom), 138.4 (C_q_ arom), 136.3 (C_q_ arom), 129.8 (CH arom), 129.0 (CH arom), 128.8 (CH arom), 128.3 (CH arom), 127.6 (CH arom), 120.0 (CH arom), 119.8 (CH arom), 116.7 (CH arom), 114.5 (CH arom), 108.3 (CH arom), 107.1 (CH arom), 105.4 (CH arom), 64.2 (CH_2_N), 49.0 (CH–NHC), 21.0 (CH_3_), 20.9 (CH_3_), 20.1 (CH_3_).

#### Complex **6**

In a J. Young-valved NMR tube, a suspension of complex **4b** (0.015 g, 0.02 mmol) in THF-*d*_8_ (0.5 mL) was treated with *t*BuOK (0.007 g, 0.06 mmol). The sample was analyzed by NMR spectroscopy, showing the formation of complex **6** in 75%–80% NMR yield. ^1^H NMR (400 MHz, THF-*d*_8_): δ 7.29 (dd, ^3^*J*_HH_ = 7.5 Hz, ^3^*J*_HH_ = 7.5 Hz, 1H, H arom Py), 6.81 (s, 1H, H arom Mes), 6.80 (d, ^3^*J*_HH_ = 1.6 Hz, 1H, H arom NHC), 6.72 (s, 1H, H arom Mes), 6.31 (d, ^3^*J*_HH_ = 1.6 Hz, 1H, H arom NHC), 6.16 (d, ^3^*J*_HH_ = 7.3 Hz, 1H, H arom Py), 5.95 (d, ^3^*J*_HH_ = 7.6 Hz, 1H, H arom Py), 5.48 (s,1H, NHC–CH), 3.88 (dd, ^2^*J*_HH_ = 13.6 Hz, ^3^*J*_HH_ = 2.8 Hz, 1H, C*H*HN), 3.39 (m, 1H, C*H*HN), 2.97 (br d, ^2^*J*_HH_ = 10.9 Hz, 1H, NH), 2.25 (s, 3H, CH_3_), 2.04 (s, 3H, CH_3_), 1.77 (s, 3H, CH_3_), 1.31 (s, 9H, C(CH_3_)_3_), – 16.49 (d, ^2^*J*_HH_ = 5.6 Hz, 1H, IrH), −18.41 (d, ^2^*J*_HH_ = 5.6 Hz, 1H, IrH). ^13^C{^1^H} NMR (101 MHz, THF-*d*_8_): δ 164.5 (C_q_arom), 161.5 (C_q_arom), 150.0 (C2–NHC), 140.6 (C_q_arom), 137.5 (C_q_arom), 136.3 (C_q_arom), 136.1 (C_q_arom), 131.1 (CH arom), 128.4 (CH arom), 127.9 (CH arom), 120.0 (CH arom), 116.4 (CH arom), 112.5 (CH arom), 110.2 (CH arom), 56.8 (*C*(CH_3_)_3_), 56.1 (CH_2_NH), 54.9 (CH–NHC), 29.2 (C(*C*H_3_)_3_), 21.1 (CH_3_), 20.8 (CH_3_), 20.0 (CH_3_).

#### Complex **7**

In a J. Young-valved NMR tube, a suspension of complex **4b** (0.015 g, 0.02 mmol) in THF-*d*_8_ (0.5 mL) was treated with *t*BuOK (0.007 g, 0.06 mmol). The sample was pressurized with 4 bar H_2_, and analyzed by NMR spectroscopy after 48 h. ^1^H NMR (400 MHz, THF-*d*_8_): δ 7.52 (dd, ^3^*J*_HH_ = 7.6 Hz, ^3^*J*_HH_ = 7.6 Hz, 1H, H arom Py), 7.15 (m, 2H, 2 H arom Py), 7.00 (d, ^3^*J*_HH_ = 1.9 Hz, 1H, H arom NHC), 6.82 (s, 1H, H arom Mes), 6.80 (s, 1H, H arom Mes), 6.56 (d, ^3^*J*_HH_ = 1.9 Hz, 1H, H arom NHC), 5.10 (d, ^2^*J*_HH_ = 13.5 Hz,1H, C*H*H–NHC), 5.02 (br, 1H, NH), 5.02 (d, ^2^*J*_HH_ = 13.5 Hz,1H, C*H*H-NHC), 4.25 (dd, ^2^*J*_HH_ = 14.4 Hz, ^3^*J*_HH_ = 3.8 Hz, 1H, C*H*HN), 4.02 (dd, ^2^*J*_HH_ = 14.4 Hz, ^3^*J*_HH_ = 5.8 Hz, 1H, C*H*HN), 2.27 (s, 3H, CH_3_), 2.03 (s, 3H, CH_3_), 1.95 (s, 3H, CH_3_), 1.11 (s, 9H, C(CH_3_)_3_), −8.92 (m, 2H, 2 IrH), −18.24 (dd, ^2^*J*_HH_ = 5.2 Hz, ^2^*J*_HH_ = 5.2 Hz, 1H, IrH). ^13^C{^1^H} NMR (101 MHz, THF-*d*_8_): δ 164.5 (C_q_arom), 164.0 (C_q_arom), 153.8 (C2–NHC), 139.5 (C_q_arom), 137.3 (C_q_arom), 136.7 (C_q_arom), 131.5 (CH arom), 128.6 (CH arom), 128.5 (CH arom), 120.9 (CH arom), 119.1 (CH arom), 118.6 (CH arom), 117.9 (CH arom), 59.0 (CH_2_–NHC), 57.7 (CH_2_NH), 50.7 (*C*(CH_3_)_3_), 27.5 (C(*C*H_3_)_3_), 21.1 (CH_3_), 19.6 (CH_3_), 19.4 (CH_3_); signals of two quaternary aromatic carbons could not be unambiguously assigned.

### Ammonia-Borane Dehydrogenation

#### Experimental Setup for H_2_ Production

H_2_ generation was followed up using a Fisher-Porter vessel (25 mL) that was connected to a vacuum line and coupled to an ESI pressure gauge model GS4200-USB (0–6 bar) that was connected to a computer (see the Supporting Information for details).

#### Representative Procedure for AB Dehydrogenation

A solution of complex **4a** (4.3 mg, 6.5 μmol) and *t*BuOK (1.8 mg, 0.016 mmol) in THF (0.5 mL) was added to a freshly prepared, stirred (750 rpm) solution of AB (50.0 mg, 1.62 mmol) in THF (0.5 mL) at room temperature. H_2_ generation was monitored by registering the increase of pressure in the system. At the end of the reaction, the supernatant was transferred to an NMR tube and analyzed via ^11^B NMR spectroscopy, to identify the soluble reaction byproducts. The insoluble residue was washed subsequently with THF and Et_2_O, and then dried under vacuum before recording the IR spectrum.

## Supporting Information Available

Experimental setup details, kinetic studies, selected NMR spectra, and X-ray diffraction data (PDF)
Cartesian coordinates, absolute Gibbs energies (THF) and imaginary frequencies (TSs) of the DFT-optimized structures (XYZ)

## Supplementary Material

ic1c03056_si_001.pdf

ic1c03056_si_002.xyz

## Accession Codes

CCDC 2113023–2113025 contain the supplementary crystallographic data for this paper. These data can be obtained free of charge via www.ccdc.cam.ac.uk/data_request/cif, or by emailing data_request@ccdc.cam.ac.uk, or by contacting The Cambridge Crystallographic Data Centre, 12 Union Road, 909 Cambridge CB2 1EZ, UK; fax: + 44 1223 336033.
