# Supplementary material for: Ammonia–Borane Dehydrogenation Catalyzed by Dual-Mode Proton-Responsive Ir-CNNH Complexes
Source: Inorg Chem. 2021 Nov 16;60(23):18490–502. doi: 10.1021/acs.inorgchem.1c03056 (PMC8653221; doi:10.1021/acs.inorgchem.1c03056)
Supplement: Supplementary file 1 — ic1c03056_si_001.pdf [file ic1c03056_si_001.pdf]

Ammonia–Borane Dehydrogenation Catalyzed by Dual Mode Proton-Responsive Ir-CNN^{H} Complexes

Isabel Ortega-Lepe,[a] Andrea Rossin,*[b] Práxedes Sánchez,[a] Laura L. Santos,[a] Nuria Rendón,[a] Eleuterio Álvarez,[a] Joaquín López-Serrano,[a] and Andrés Suárez*[a]

[a] *Instituto de Investigaciones Químicas (IIQ), Departamento de Química Inorgánica and Centro de Innovación en Química Avanzada (ORFEO-CINQA), CSIC and Universidad de Sevilla.*

[b] *Istituto di Chimica dei Composti Organometallici - Consiglio Nazionale delle Ricerche (ICCOM - CNR).*

E-mails: a.rossin@iccom.cnr.it; andres.suarez@iiq.csic.es

Table of Contents

1. Dynamic behavior of complexes **2a** and **2b**……….……………………………..…..……….3

2. Experimental set-up for hydrogen evolution measurements …………………..………....6

3. Characterization of the H_2-depleted byproducts…………………...……...……..……….…..7

4. Kinetic measurements………………………………………………………………………9

5. DOSY experiments……………………..………………………………………..……..14

6. Selected NMR spectra for complexes **2-6**………………..……….…………………..…..16

7. Comparison of the catalytic activity of **4a** with previously reported catalysts…………….32

8. DFT calculations of the reaction catalyzed by **6**…….…………..………………………..33

9. Crystal X-ray structure analysis for complexes **3**, **4b** and **5**………………………………..35

1. Dynamic behavior of complexes 2a and 2b

In the ^{1}H-^{1}H EXSY spectra of complexes **2a** and **2b** registered at room temperature, pairwise exchanges of the olefinic signals corresponding to different olefinic moieties are observed (Figures S2-S5). This dynamic behavior can be ascribed to alkene site exchange allowed by the decoordination of the C=C fragment trans to the NHC moiety to produce the distorted tetrahedral intermediate **A**, followed by re-coordination of the free olefin moiety to the opposite side without a net change of the fac coordination mode of the CNN^{H} ligand (Figure S1).[1]

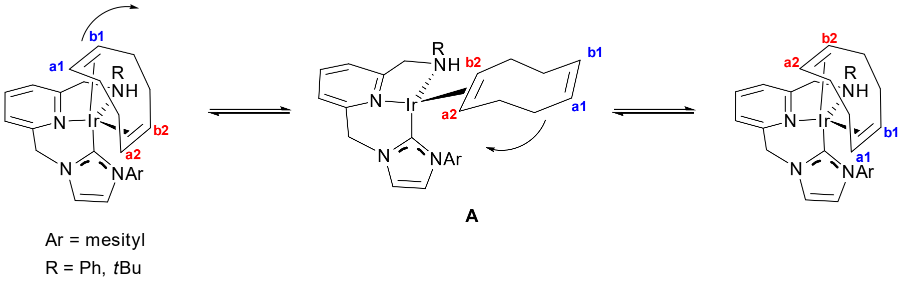

Figure S1. Proposed dynamic process in solution operating in the cationic part of complexes **2a** and **2b** (positive charges have been suppressed for clarity).

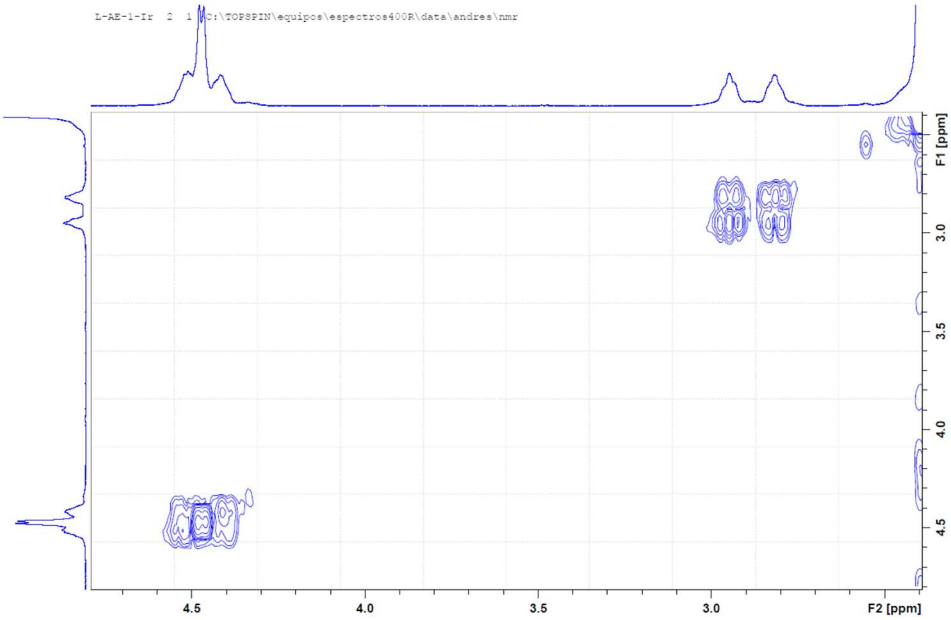

Figure S2. Region of the ^{1}H-^{1}H COSY spectrum of **2a** (400 MHz, CD_2Cl_2).

[1] Sánchez, P.; Hernández-Juárez, M.; Álvarez, E.; Paneque, M.; Rendón, N.; Suárez, A. *Dalton Trans.* **2016**, *45*, 16997.

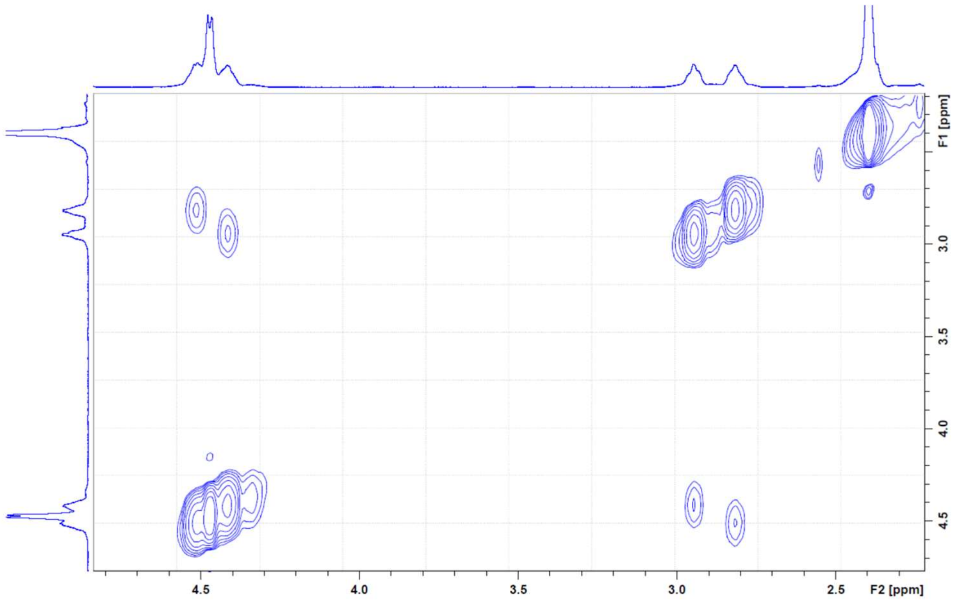

Figure S3. Region of the ^1H-^1H NOESY spectrum of **2a** (400 MHz, CD_2Cl_2, 298 K).

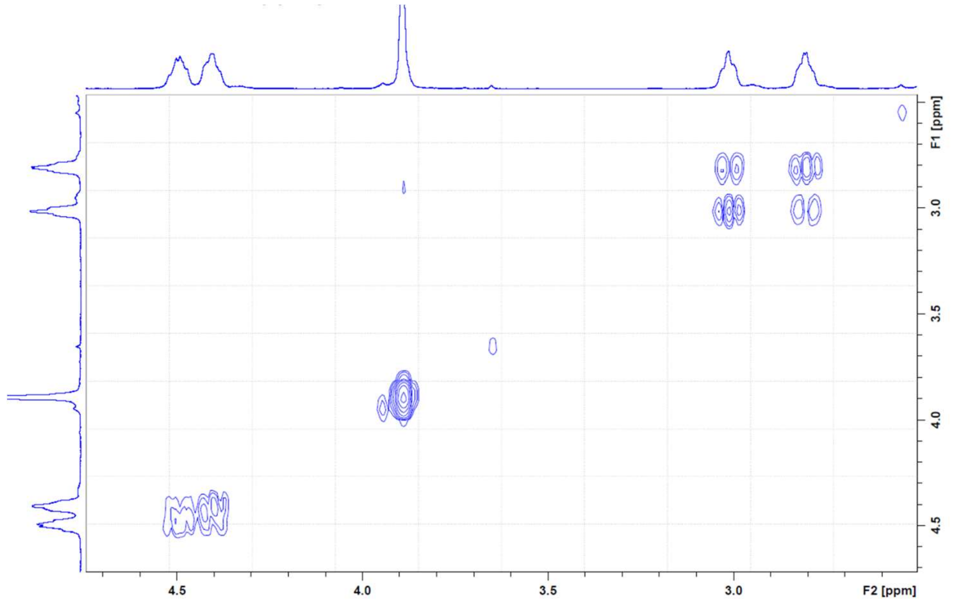

Figure S4. Region of the ^1H-^1H COSY spectrum of **2b** (400 MHz, CD_2Cl_2).

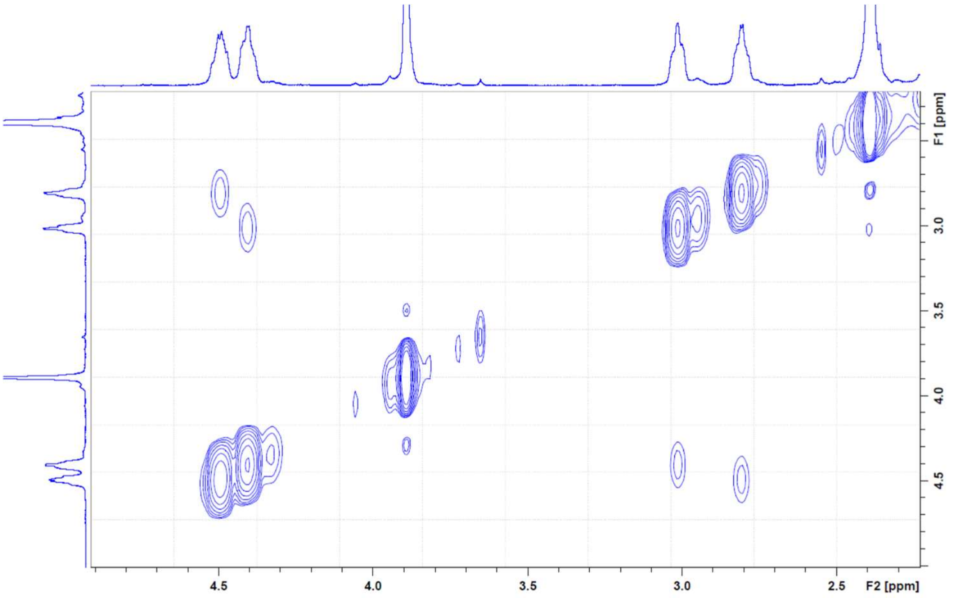

Figure S5. Region of the ^1H-^1H NOESY spectrum of **2b** (400 MHz, CD_2Cl_2, 298 K).

2. Experimental set-up for hydrogen evolution measurements

Gas evolution measurements were registered using an in-house made experimental system consisting of a Fisher-Porter vessel (10 mL) connected to a vacuum line and coupled to a ESI pressure gauge model GS4200-USB (0–6 bar)[2] plugged to a computer (Figure S6).[3] In ammonia-borane dehydrogenation experiments, a solution of the catalyst and *t*BuOK in THF (0.5 mL) was added via syringe to the THF (0.5 mL) solution of AB. The reaction was monitored by measuring the pressure variation *vs.* time at regular intervals of 2-5 s in the closed reaction vessel.

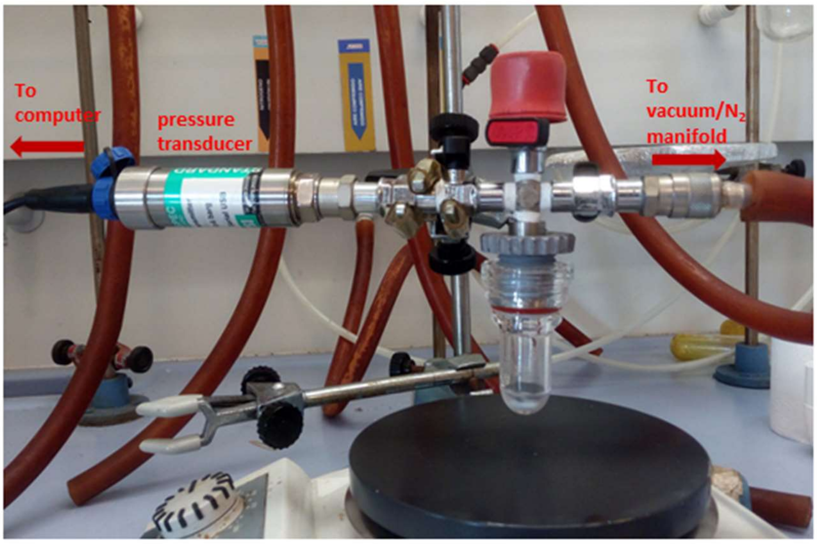

Figure S6. Experimental set-up for gas evolution measurements.

[2] https://www.esi-tec.com/

[3] Lara, P.; Philippot, K.; Suárez, A. *ChemCatChem* **2019**, *11*, 766.

3. Characterization of the H_2-depleted byproducts

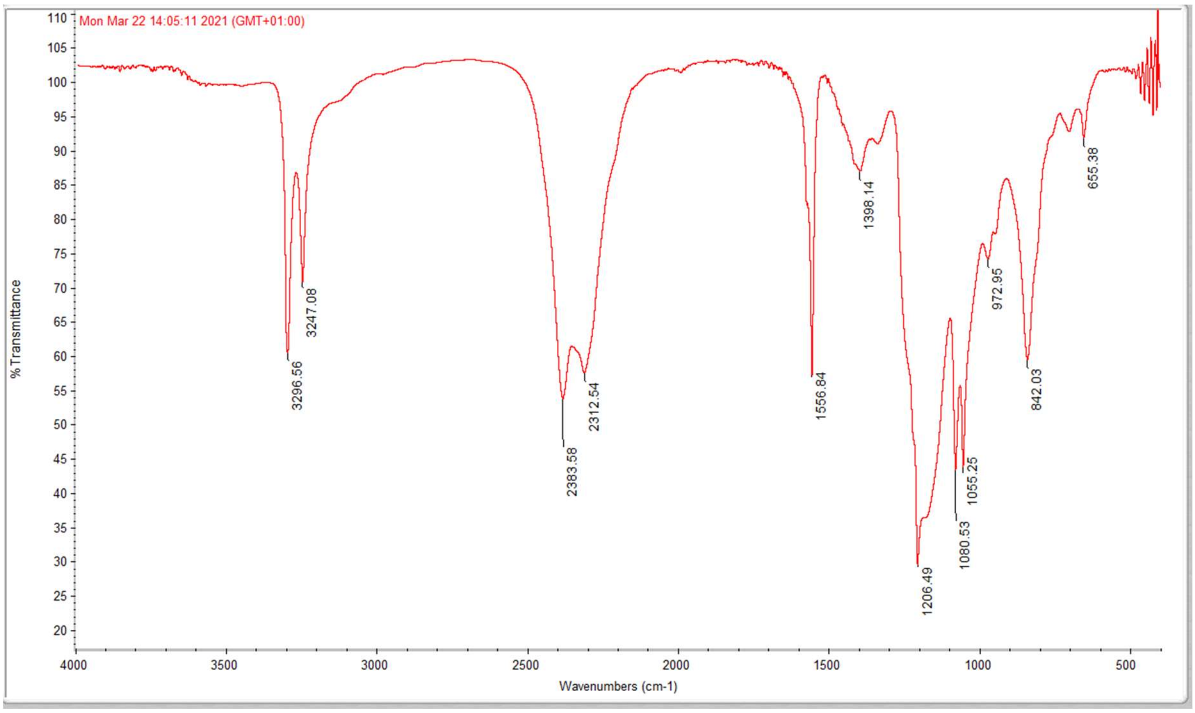

Figure S7. ATR-IR spectrum of the insoluble H_2-depleted material produced in the dehydrogenation of AB with **4a**.

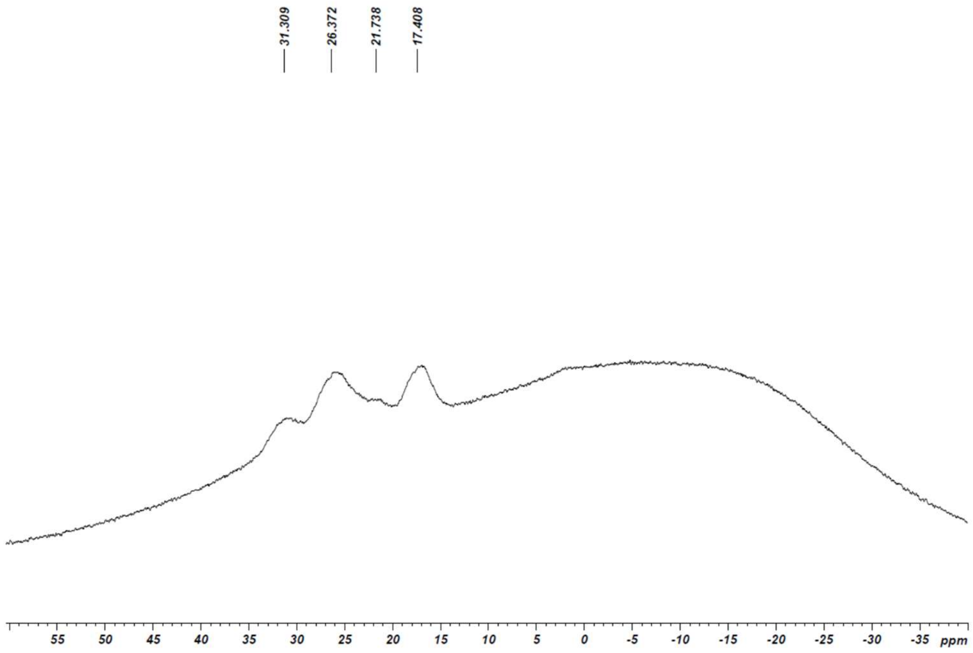

Figure S8. ^{11}B NMR spectrum (128 MHz) of the soluble H_2-depleted material produced in the dehydrogenation of AB with **4a**.

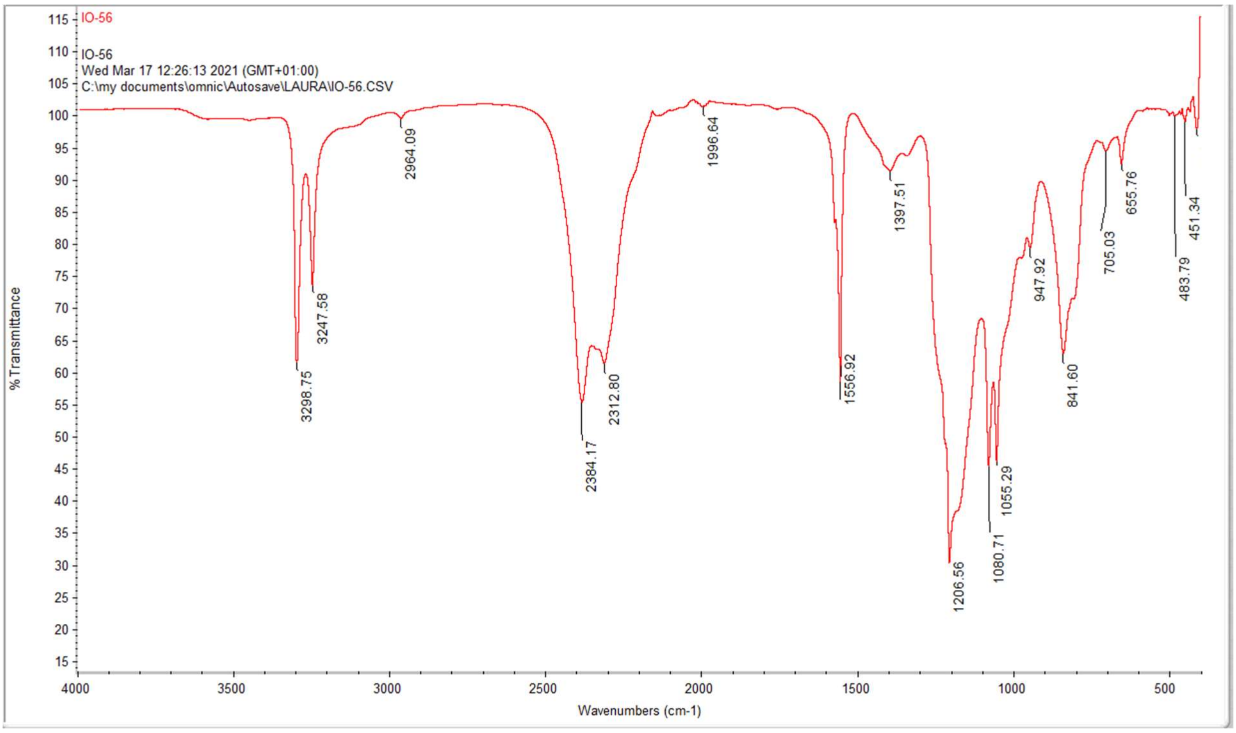

Figure S9. ATR-IR spectrum of the insoluble H_2-depleted material produced in the dehydrogenation of AB with **4b**.

4. Kinetics measurements

4.1. Kinetic measurements for the dehydrogenation of AB catalyzed by 4a

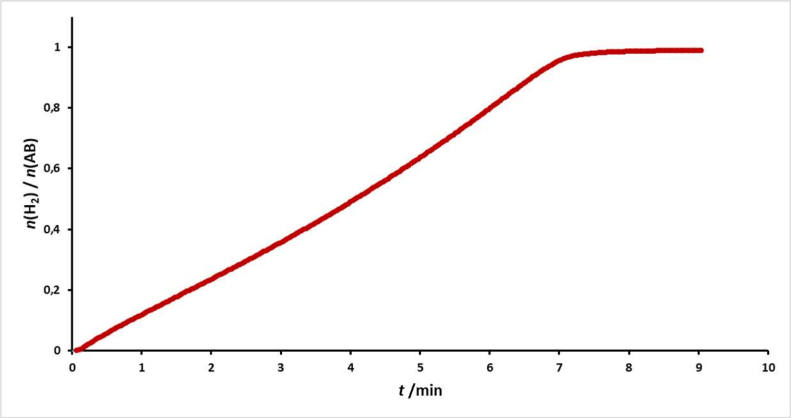

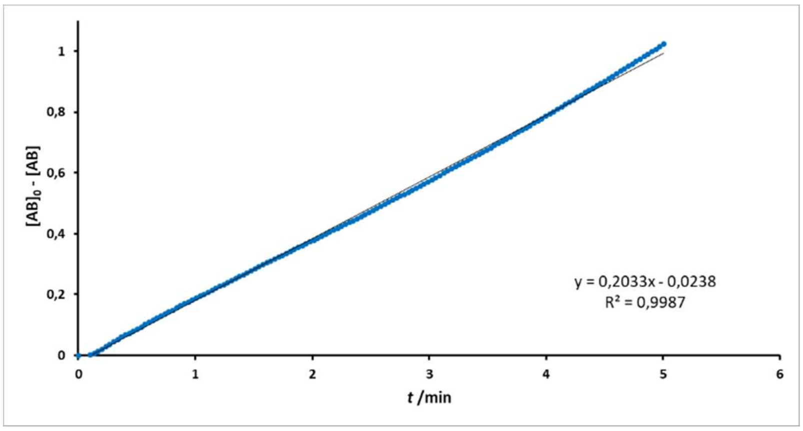

Figure S10. (top) H_2 evolution in the catalytic dehydrogenation of AB with **4a** (0.4 mol%) in THF at room temperature ([AB] = 1.62 M; *t*BuOK/**4a** = 2.5); (bottom) $[AB]_0$-[AB] *vs.* time (min) plot (zero order rate law).

The reaction order with respect to AB in the dehydrogenation reaction with **4a** was determined through an initial rate experiment at 298 K by varying AB concentrations (1.1 M – 2.3 M) and keeping constant the catalyst concentration ([**4a**] = 6.5 × 10^{-3} M). The reaction rates v_o were inferred for each separate reaction, and the slope of the plot of ln(v_o) *vs.* ln([AB]) provided the reaction rate dependence of the substrate.

[AB] (M)	v_o (M min^{-1})
1.13	0.177
1.62	0.207
2.27	0.162

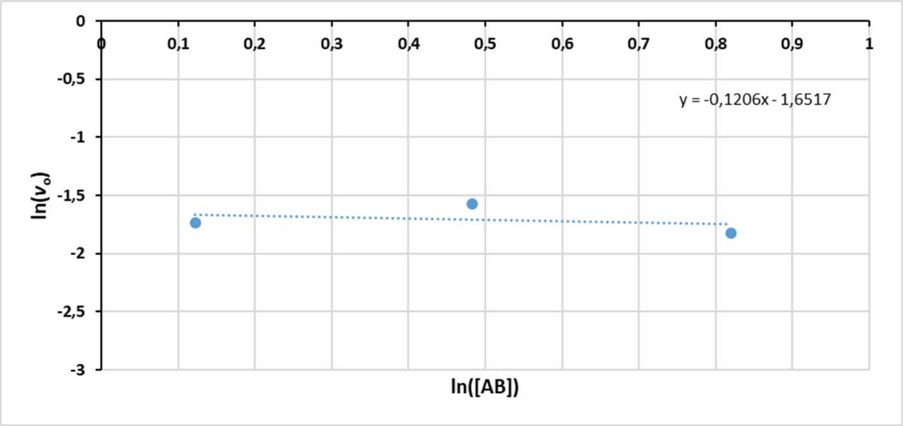

Figure S11. Plot of ln(v_o) *vs.* ln[AB] for the determination of the reaction order of AB.

The reaction order with respect to **4a** was determined through an initial rate experiment at 298 K by varying the catalyst concentration (7.0 × 10^{-3} M – 3.8 × 10^{-3} M) and keeping constant [AB] = 1.62 M. The slope of the plot of ln(v_o) *vs.* ln([**4a**]) provides the reaction rate dependence from [**4a**].

[**4a**] (× 10^{-3} M)	v_o (M min^{-1})
7.0	17.1 × 10^{-2}
6.1	15.0 × 10^{-2}
5.3	13.0 × 10^{-2}
4.6	11.1 × 10^{-2}
3.8	8.8 × 10^{-2}

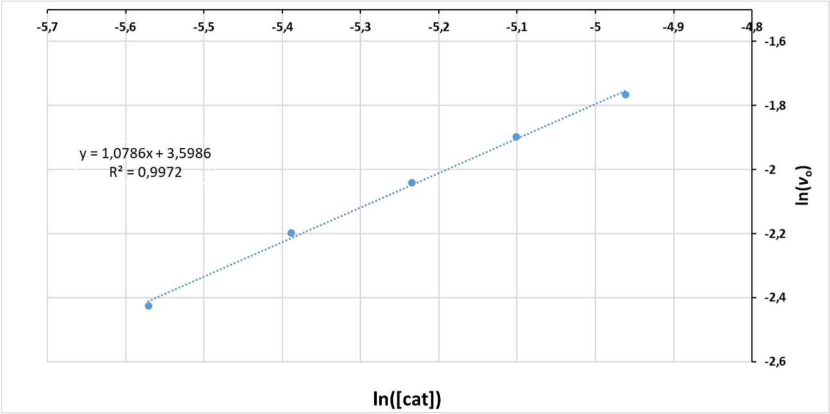

Figure S12. Plot of ln(v_o) *vs.* ln[**4a**] for the determination of the reaction order of **4a**.

4.2. Kinetic measurements for the dehydrogenation of AB catalyzed by 4b-c

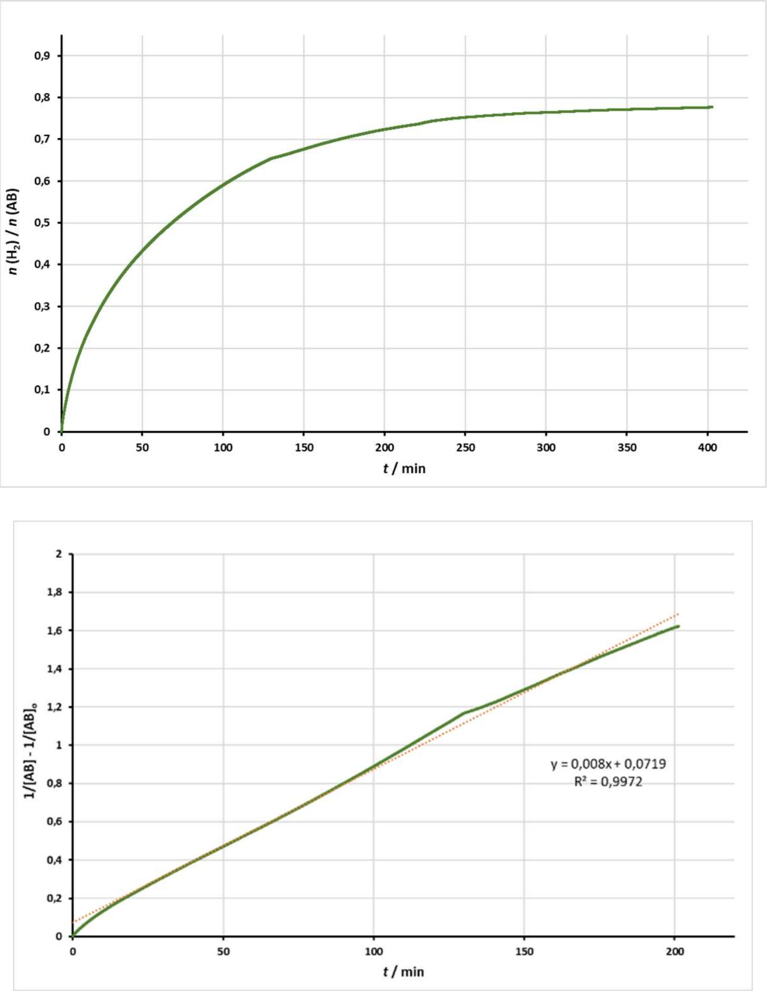

Figure S13. (top) H_2 evolution in the catalytic dehydrogenation of AB with **4b** (0.2 mol%) in THF at room temperature ([AB] = 1.62 M; *t*BuOK/**4b** = 2.5); (bottom) (1/[AB] – $1/[AB]_0$) *vs.* time plot (second order rate law).

The reaction order with respect to AB in the dehydrogenation reaction with **4b** was determined through an initial rate experiment at 298 K by varying AB concentrations (1.1 M – 2.3 M) and keeping constant the catalyst concentration ([**4b**] = 13.0 × 10^{-3} M). The reaction rates v_o were inferred for each separate reaction, and the slope of the plot of ln(v_o) *vs.* ln([AB]) provided the reaction rate dependence of the substrate.

[AB] (M)	v_o (M^{-1} min^{-1})
1.13	9.3×10^{-3}
1.62	15.6×10^{-3}
2.27	34.3×10^{-3}

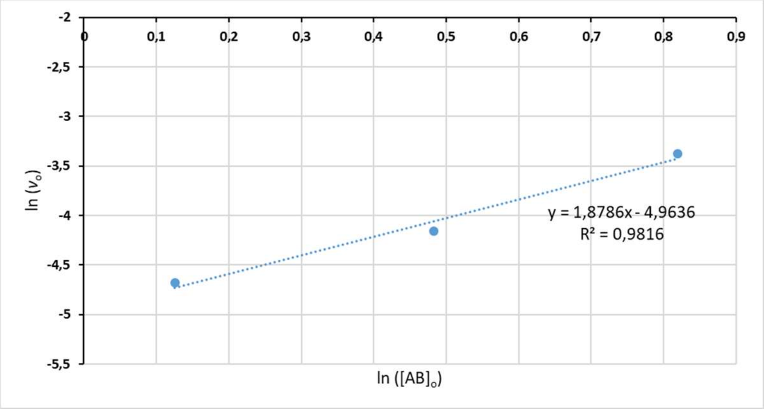

Figure S14. Plot of ln(v_o) *vs.* ln[AB] for the determination of the reaction order of AB.

The reaction order with respect to **4b** was determined through an initial rate experiment at 298 K by varying the catalyst concentration (9.7×10^{-3} M – 18.1×10^{-3} M) and keeping constant [AB] = 1.62 M. The v_o values were determined considering second order kinetics with respect to [AB]. The slope of the plot of ln(v_o) *vs.* ln([**4b**]) provides the reaction rate dependence of [**4b**].

[**4b**] ($\times 10^{-3}$ M)	v_o (M^{-1} min^{-1})
18.1	24.0×10^{-3}
14.5	16.9×10^{-3}
12.9	14.8×10^{-3}
11.3	13.3×10^{-3}
9.7	12.3×10^{-3}

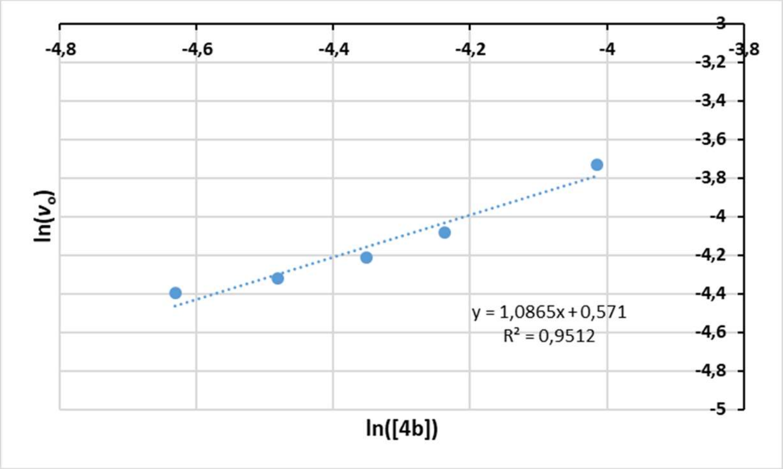

Figure S15. Plot of ln(v_o) *vs.* ln([**4b**]) for the determination of the catalyst reaction order.

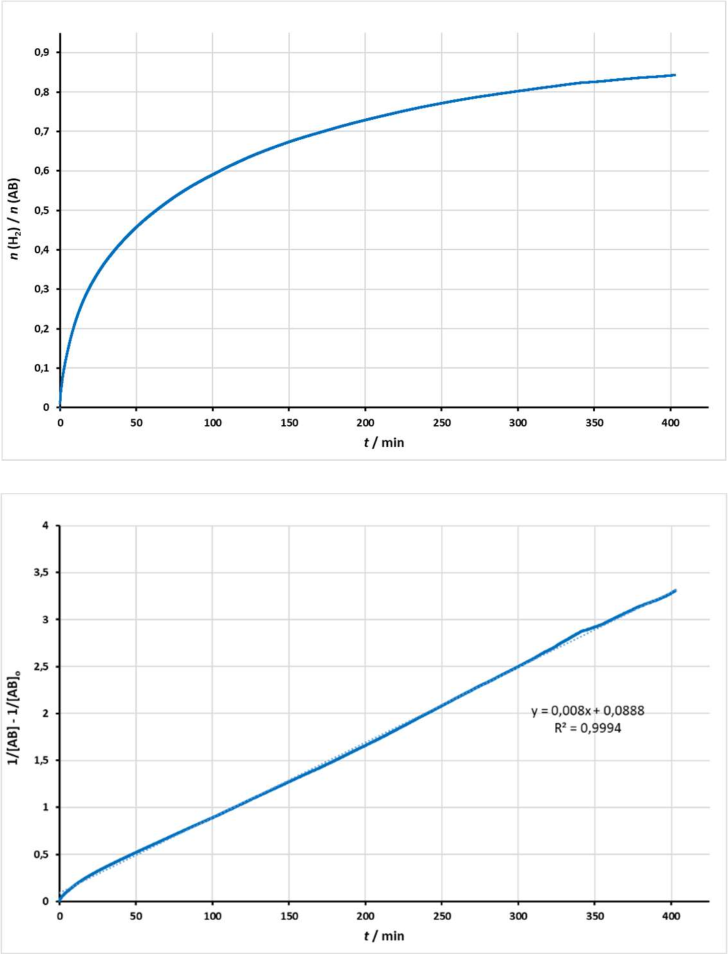

Figure S16. (top) H_2 evolution in the catalytic dehydrogenation of AB with **4c** (0.2 mol%) in THF at room temperature ([AB] = 1.62 M; *t*BuOK/**4c** = 2.5); (bottom) (1/[AB] – 1/[AB]$_0$) *vs.* time plot (second order rate law).

5. DOSY experiments

Diffusion-Ordered SpectrosopY (DOSY) NMR experiments were used to assess the nature of **5** in solution. The results obtained on this species were compared with DOSY experiments performed on **4b**, which was used as a reference monomeric species.

Experiments were performed on a BRUKER ASCEND 400 MHz spectrometer, equipped with a BBOF probe. Samples were prepared in THF-d_8 and measured at rt (25º) using standard procedures. The standard ledbpgp2s1d and ledbpgp2s BRUKER pulse sequences (LED with bipolar gradient pulse pair and spoil gradients) were applied for calibration of the gradient ramp and measurement of the diffusion coefficients (D), respectively. Gradients were calibrated by measuring the diffusion coefficient of residual HDO in a D_2O sample (D = $1.9 \cdot 10^{-9}$ $m^2 \cdot s^{-1}$).

Diffusion coefficients were calculated from the hydride resonances of the complexes, using the dosy2d utility of the TOPSPIN software (for pseudo 2D DOSY experiments) and from plots of $\ln(I/I_o)$ (I/I_o = signal intensity relative to signal intensity at zero gradient) *vs* the square of the gradient amplitude (G^2) and fitting of equation (1):

$$ln\left(\frac{I}{I_0}\right) = -(\gamma\delta)^2\left(\Delta - \frac{\delta}{3}\right)G^2D \qquad (1)$$

where γ = gyromagnetic ratio of 1H, G = gradient strength, δ = length of the gradient pulse (little delta), Δ = diffusion delay (big delta) (as implemented in the pulse sequence).

Both approaches revealed similar D values for **4a** and **5**, which translate to similar hydrodynamic radii (r_H), suggesting that **5** is a monomer in THF solutions**.**

Table S1. Diffusion coefficients (D) and hydrodynamic radii (r_H) from DOSY experiments.

	From 2D-DOSY		From fitting of eq. (1)†	
	D ($m^2 \cdot s^{-1}$)	r_H*(Å)	D ($m^2 \cdot s^{-1}$)	r_H*(Å)
**4a**	$1.09 \cdot 10^{-9}$	4.2	$0.82 \cdot 10^{-9}$	5.5
**5**	$0.93 \cdot 10^{-9}$	4.9	$0.72 \cdot 10^{-9}$	6.4

* The hydrodynamic radii were estimated using the Stokes-Einstein equation (2). † Average of from the two hydride resonances.

$$r_H = \frac{kT}{6\pi\eta D} \qquad (2)$$

Where k = Boltzmann constant, η = viscosity of the solvent (THF = 0.48 cP)

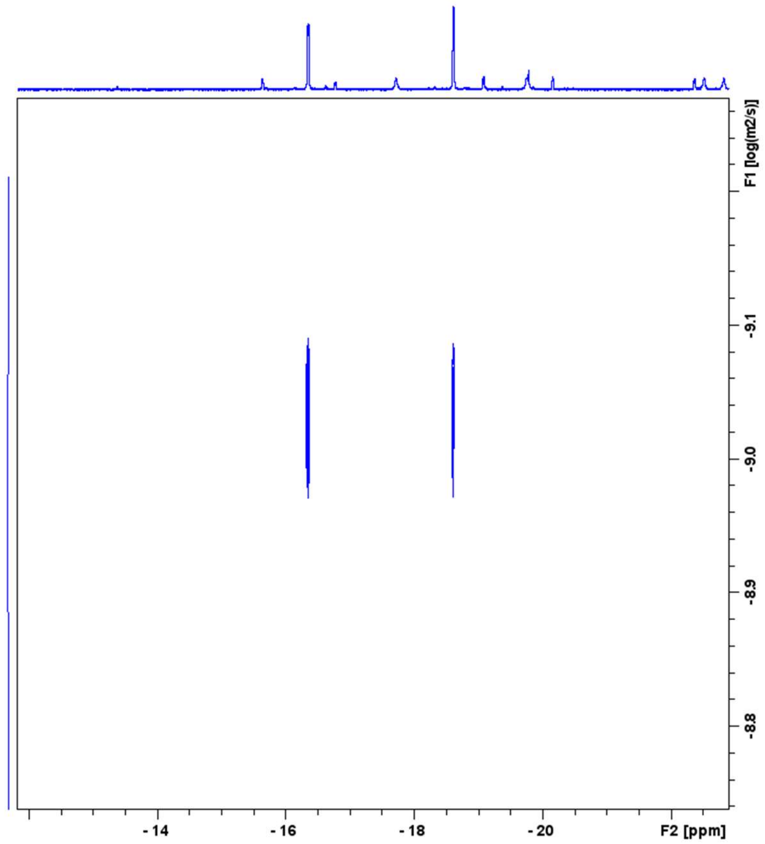

Figure S17. Hydride region of one 2D_DOSY experiment on a sample of **5.** The vertical and the horizontal axes represent log*D* ($m^2 \cdot s^{-1}$) and chemical shift, respectively.

For the sake of comparison, the X-ray volume ($V_{X\text{-ray}}$) of **5** was calculated by dividing the crystallographic unit-cell volume by the number of molecular entities contained in the unit cell, assuming that these have a spherical shape, and subtracting the van der Waals volumes of the THF-solvated K^+ ions.[4]

[4] Zhao, Y. H.; Abraham, H. M.; Zissimos, A. M. *J. Org. Chem.* **2003**, *68*, 7368.

6. Selected NMR spectra for complexes 2-6

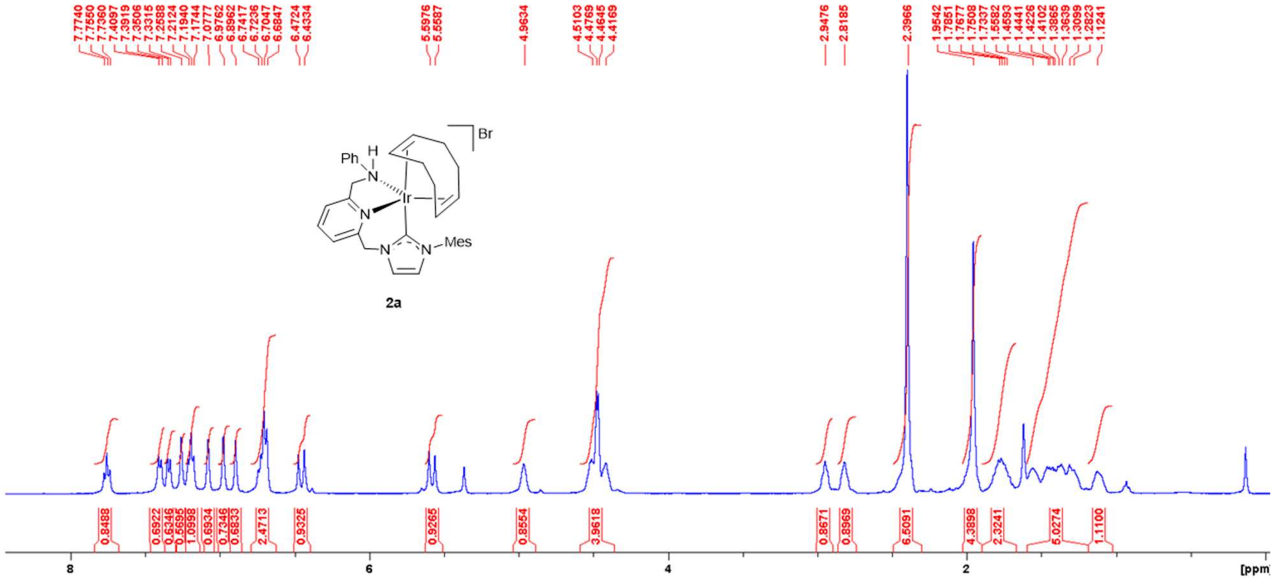

Figure S18. ^{1}H NMR spectrum (400 MHz) of **2a** in CD_2Cl_2.

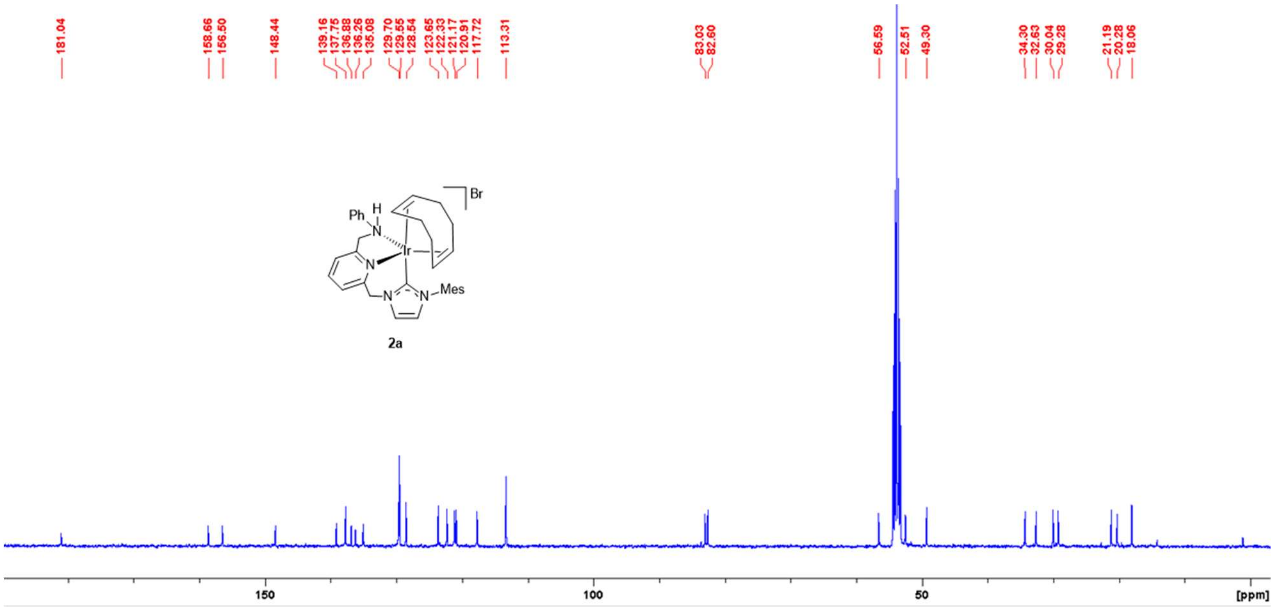

Figure S19. $^{13}C\{^{1}H\}$ NMR spectrum (101 MHz) of **2a** in CD_2Cl_2.

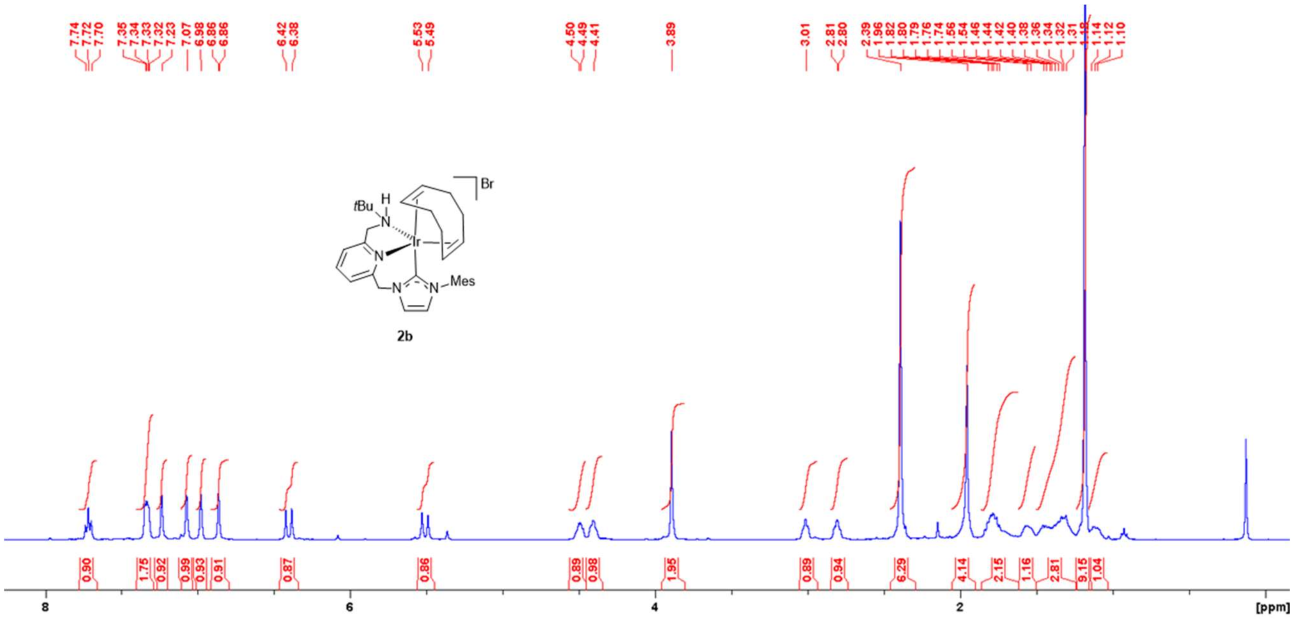

Figure S20. ^{1}H NMR spectrum (400 MHz) of **2b** in CD_2Cl_2.

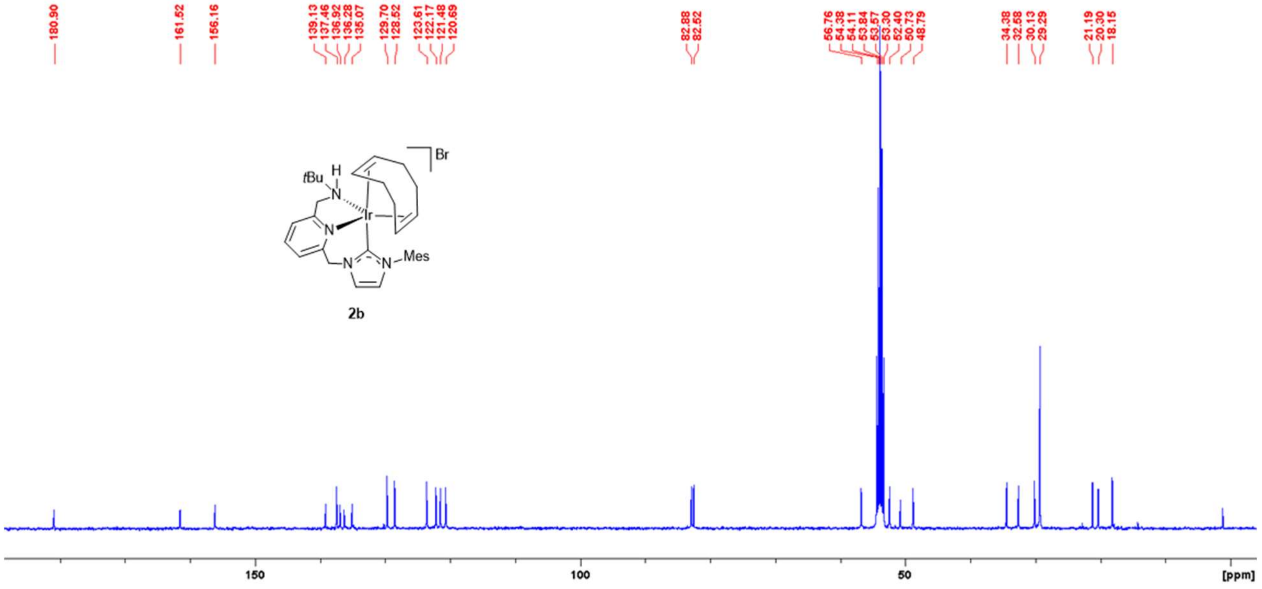

Figure S21. $^{13}C\{^{1}H\}$ NMR spectrum (101 MHz) of **2b** in CD_2Cl_2.

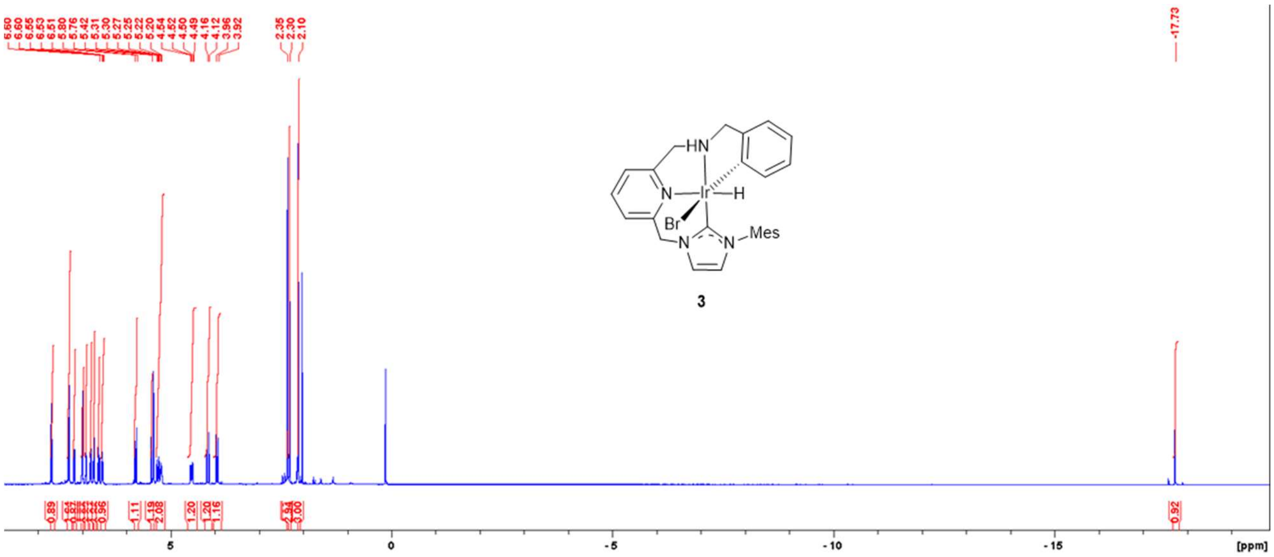

Figure S22. ^{1}H NMR spectrum (400 MHz) of **3** in CD_2Cl_2.

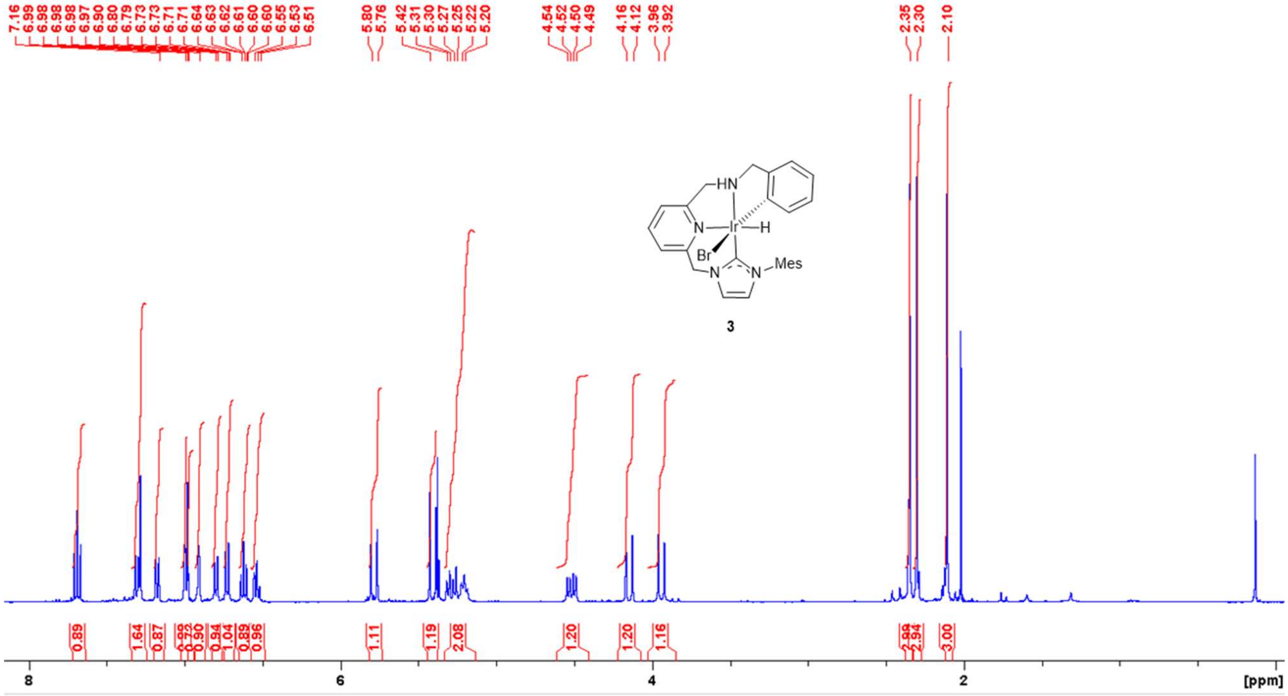

Figure S23. Region (0.0 to 8.0 ppm) of the ^{1}H NMR spectrum (400 MHz) of **3** in CD_2Cl_2.

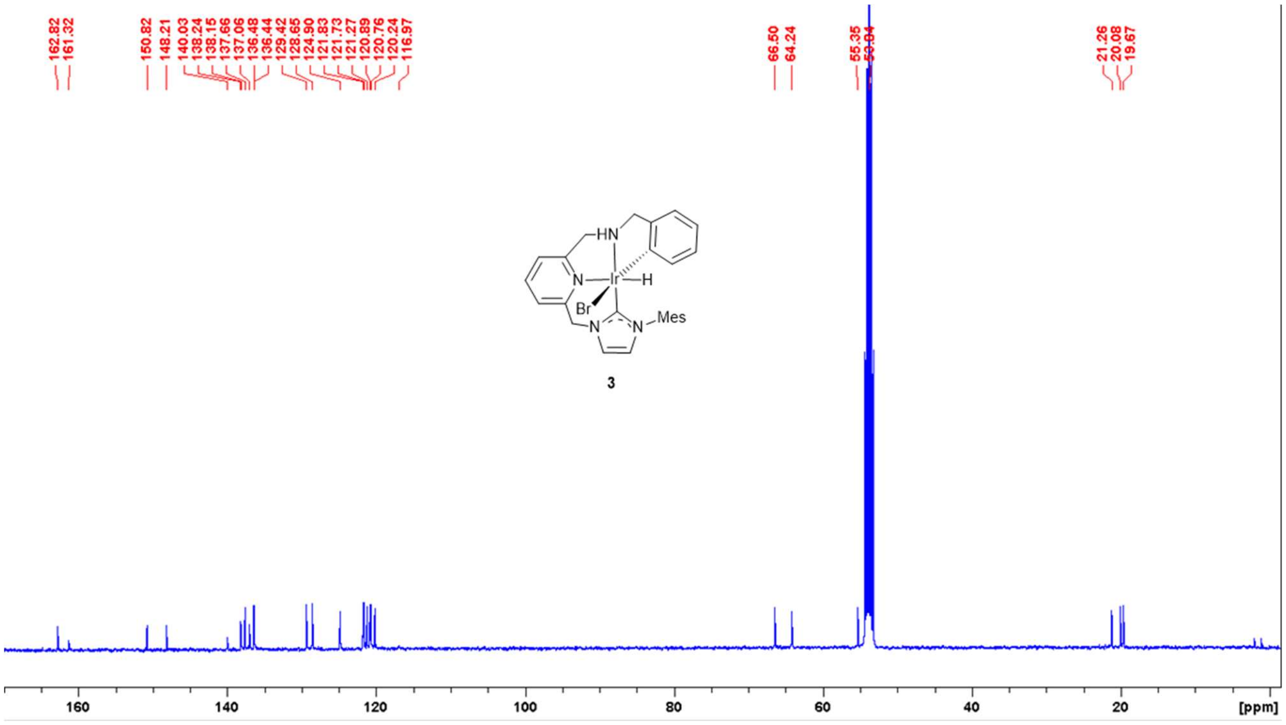

Figure S24. $^{13}C\{^{1}H\}$ NMR spectrum (101 MHz) of **3** in CD_2Cl_2.

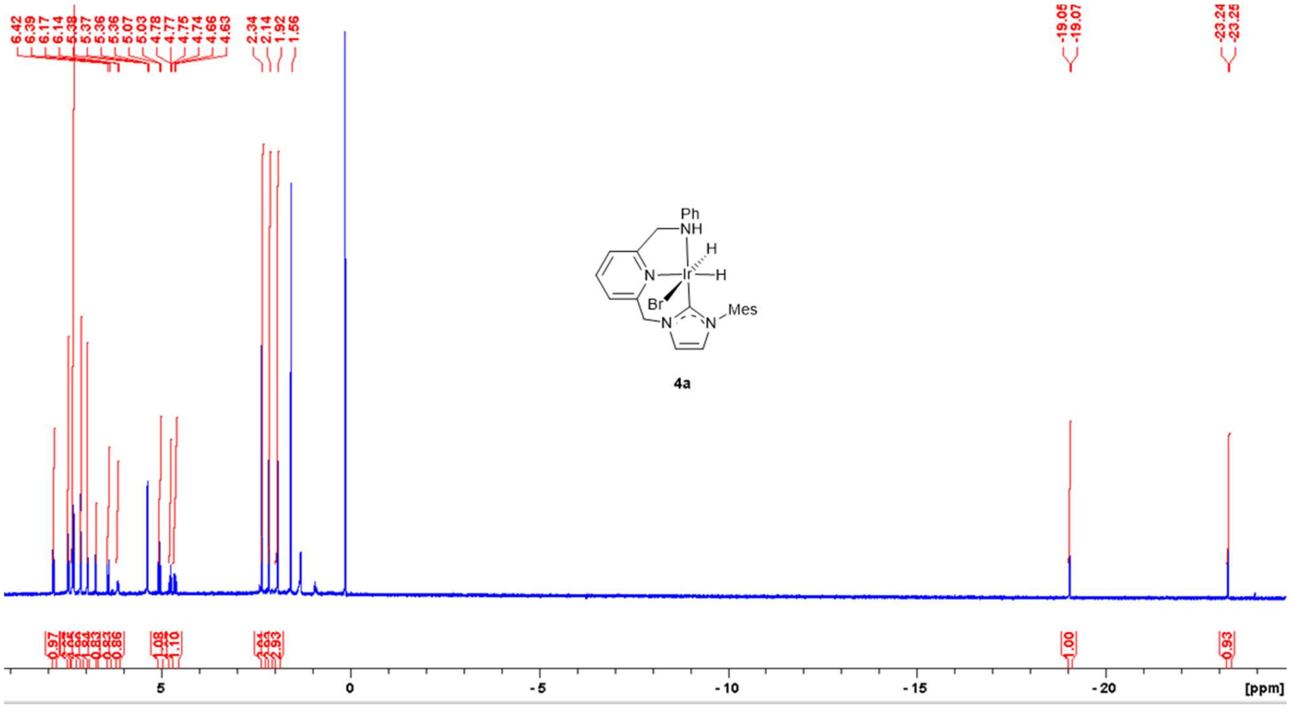

Figure S25. ^{1}H NMR spectrum (400 MHz) of **4a** in CD_2Cl_2.

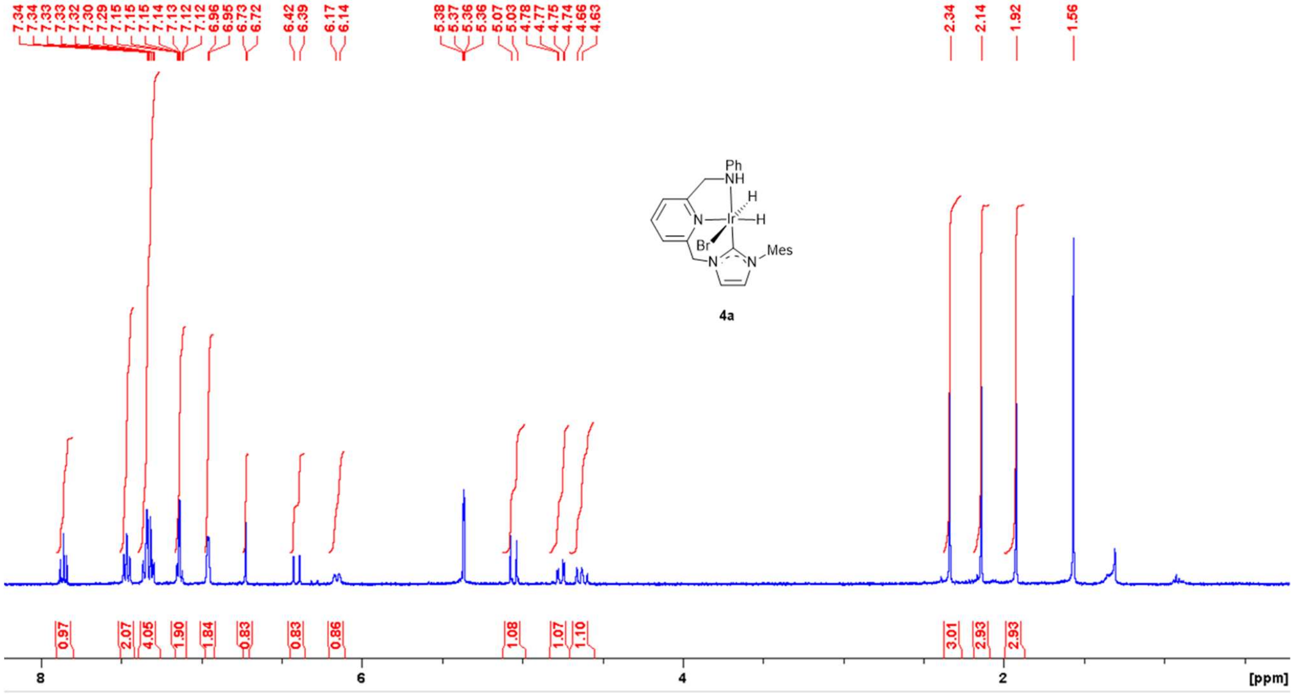

Figure S26. Region (0.0 to 8.5 ppm) of the ^{1}H NMR spectrum (400 MHz) of **4a** in CD_2Cl_2.

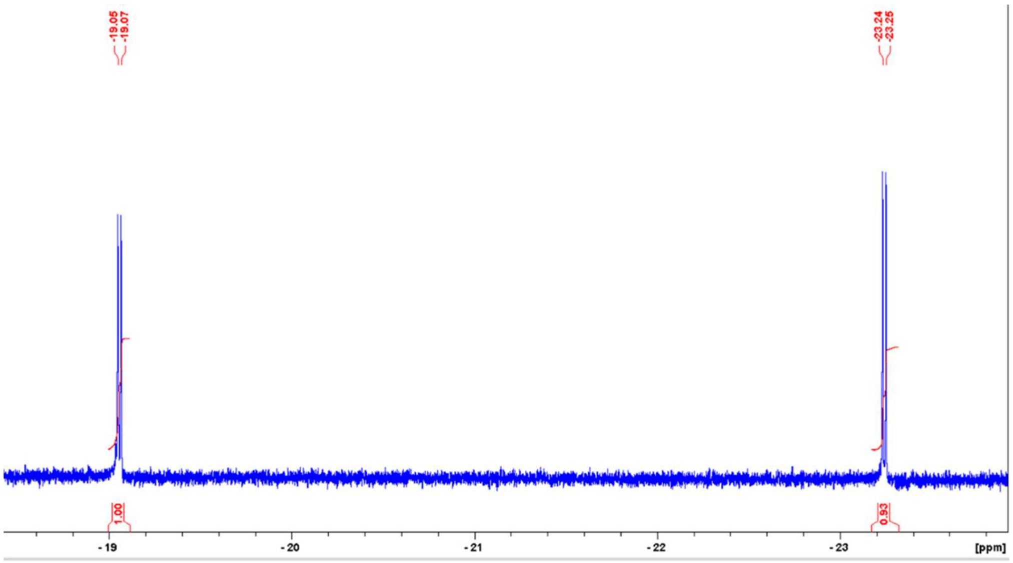

Figure S27. Hydride of the ^{1}H NMR spectrum (400 MHz) of **4a** in CD_2Cl_2.

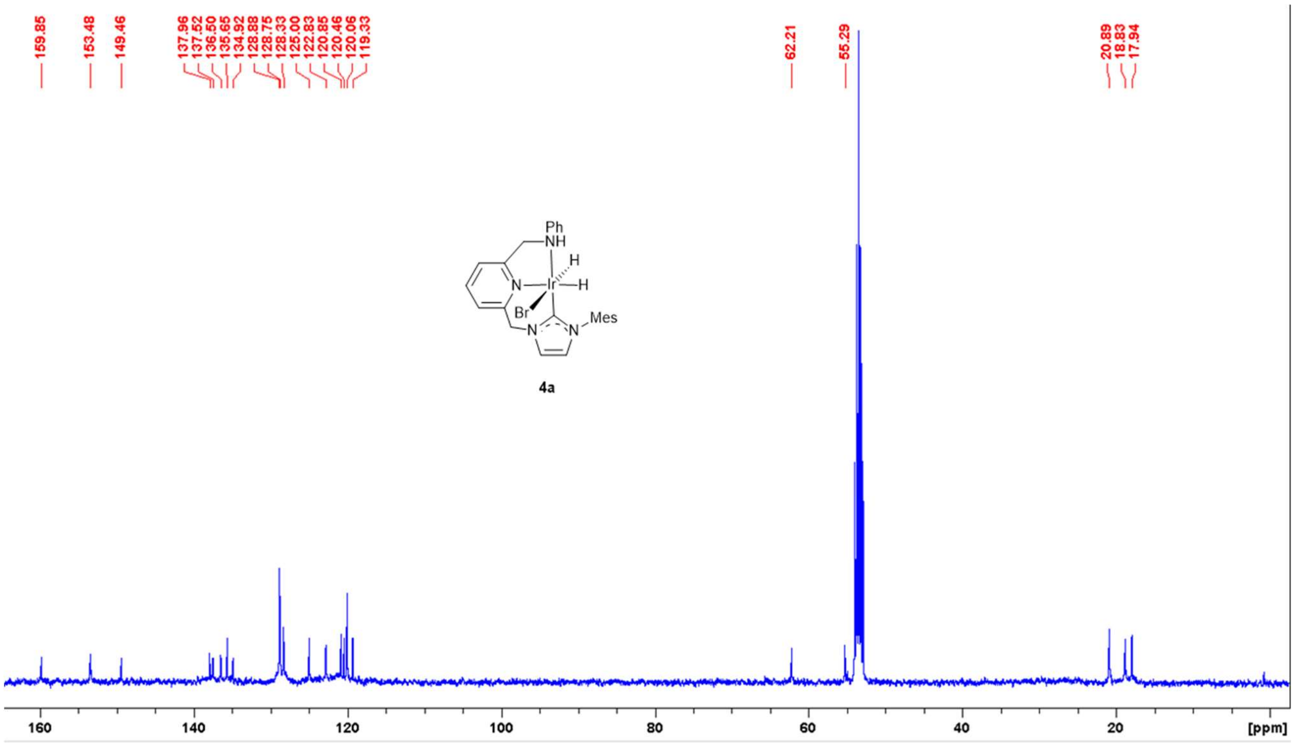

Figure S28. $^{13}C\{^{1}H\}$ NMR spectrum (101 MHz) of **4a** in CD_2Cl_2.

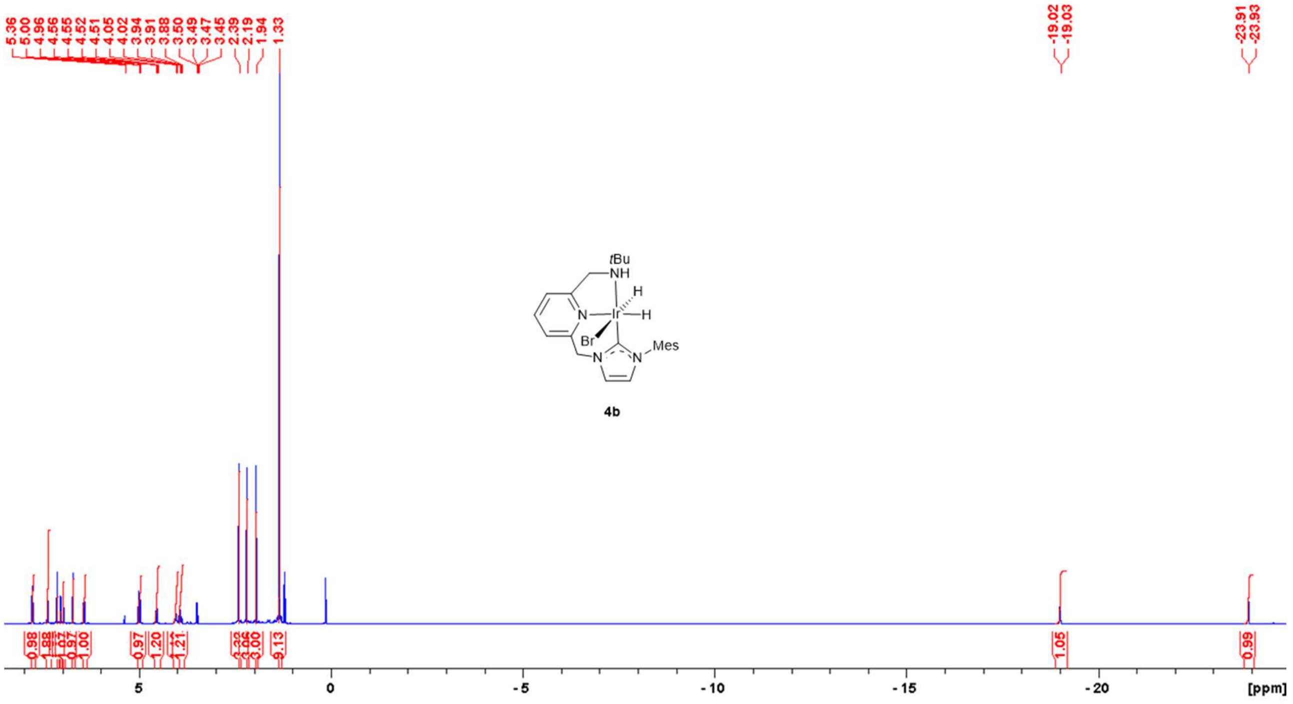

Figure S29. ^{1}H NMR spectrum (400 MHz) of **4b** in CD_2Cl_2.

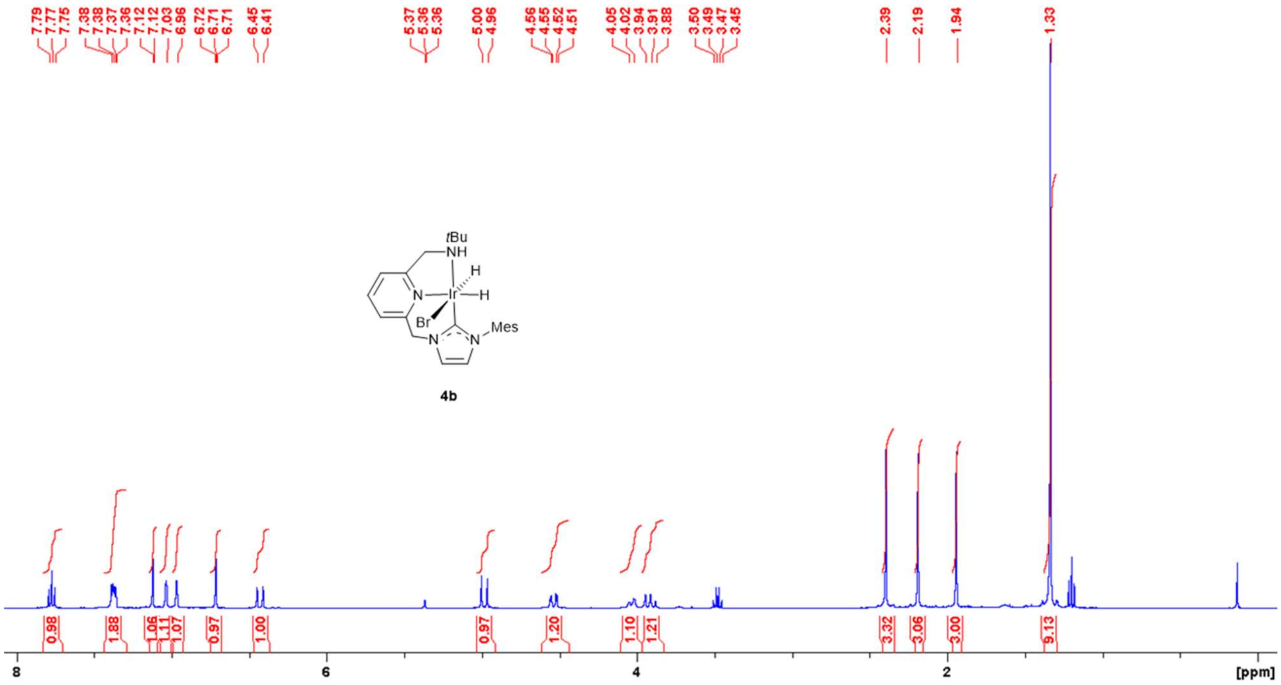

Figure S30. Region (0.0 to 8.0 ppm) of the ^{1}H NMR spectrum (400 MHz) of **4b** in CD_2Cl_2.

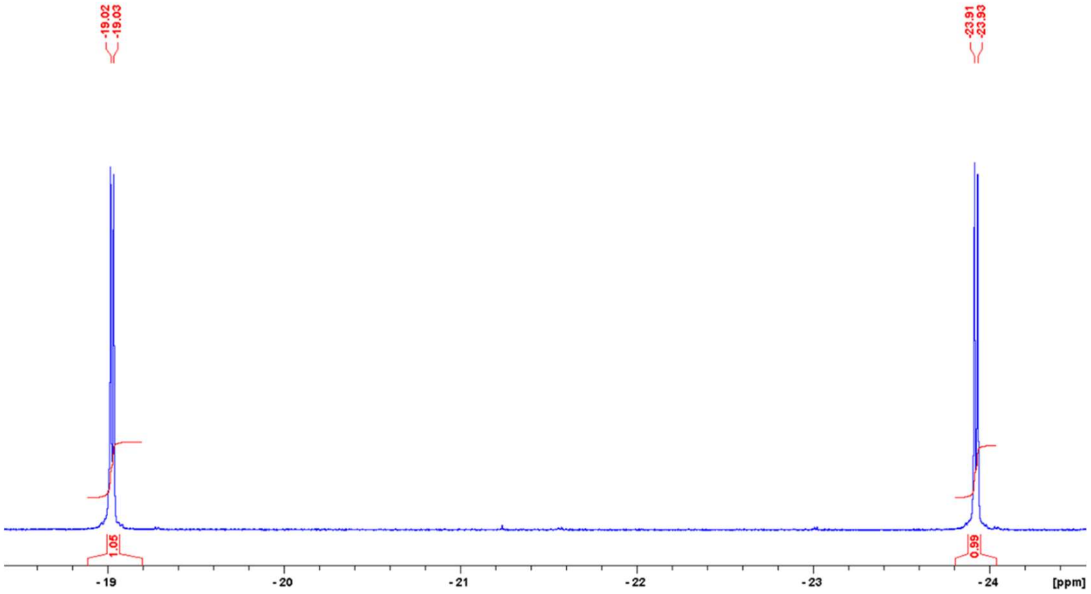

Figure S31. Hydride region of the ^{1}H NMR spectrum (400 MHz) of **4b** in CD_2Cl_2.

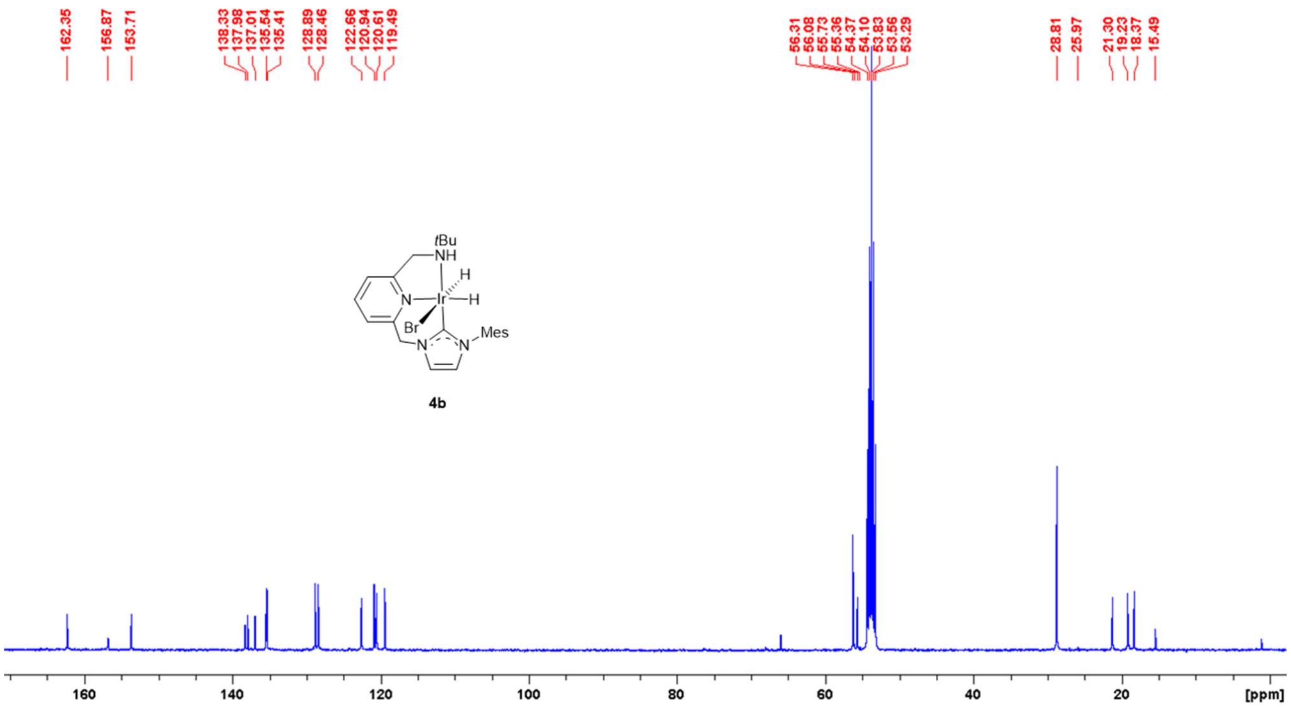

Figure S32. $^{13}C\{^{1}H\}$ NMR spectrum (101 MHz) of **4b** in CD_2Cl_2.

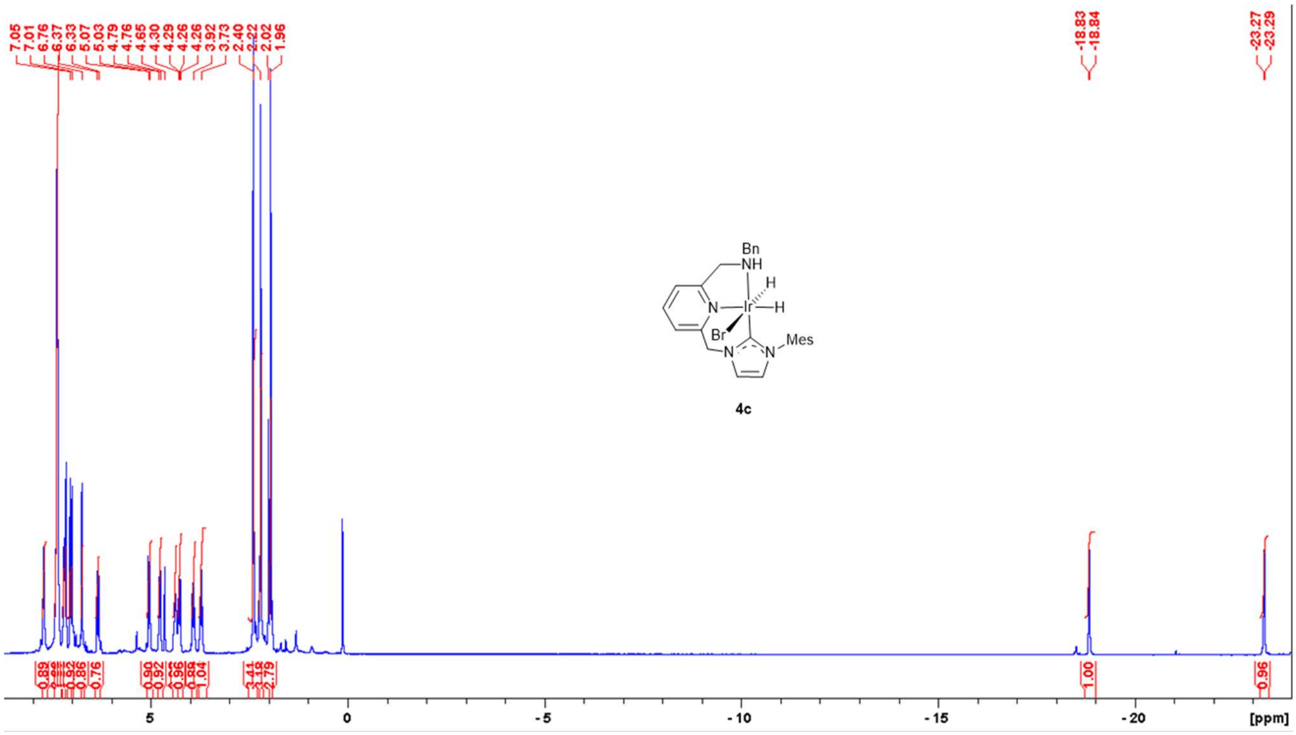

Figure S33. ^{1}H NMR spectrum (400 MHz) of **4c** in CD_2Cl_2.

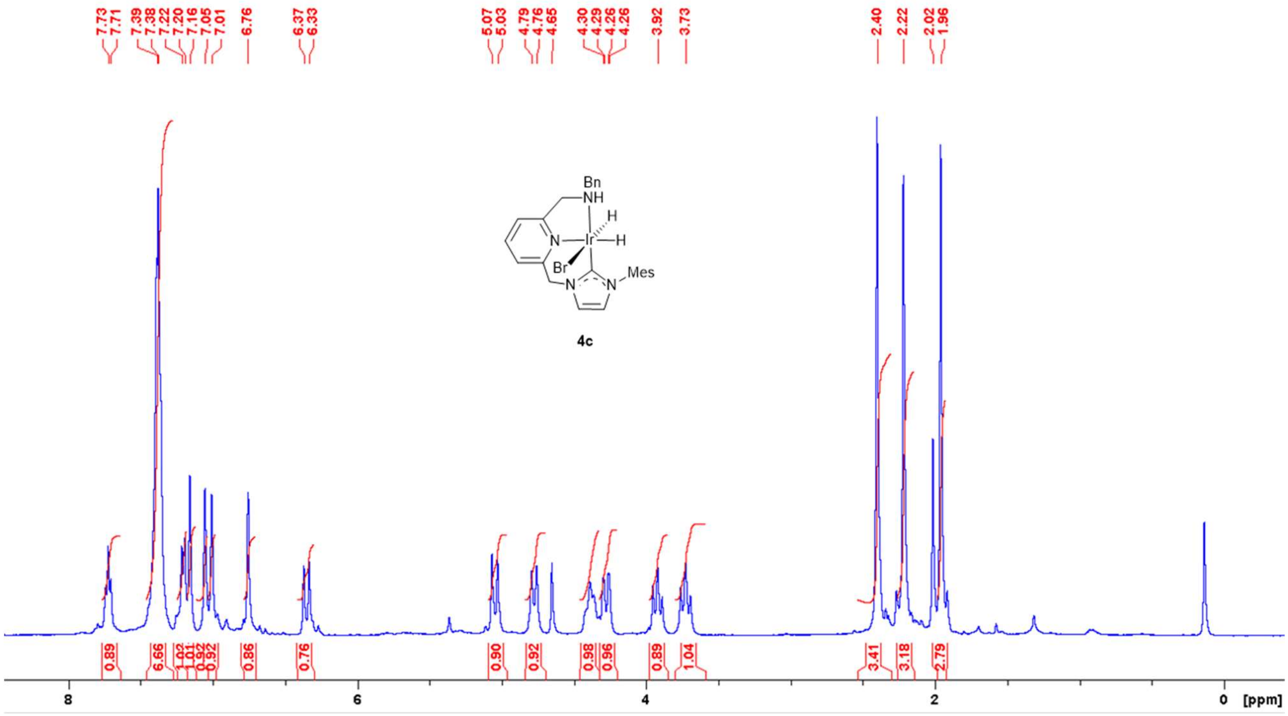

Figure S34. Region (0.0 to 8.0 ppm) of the ^{1}H NMR spectrum (400 MHz) of **4c** in CD_2Cl_2.

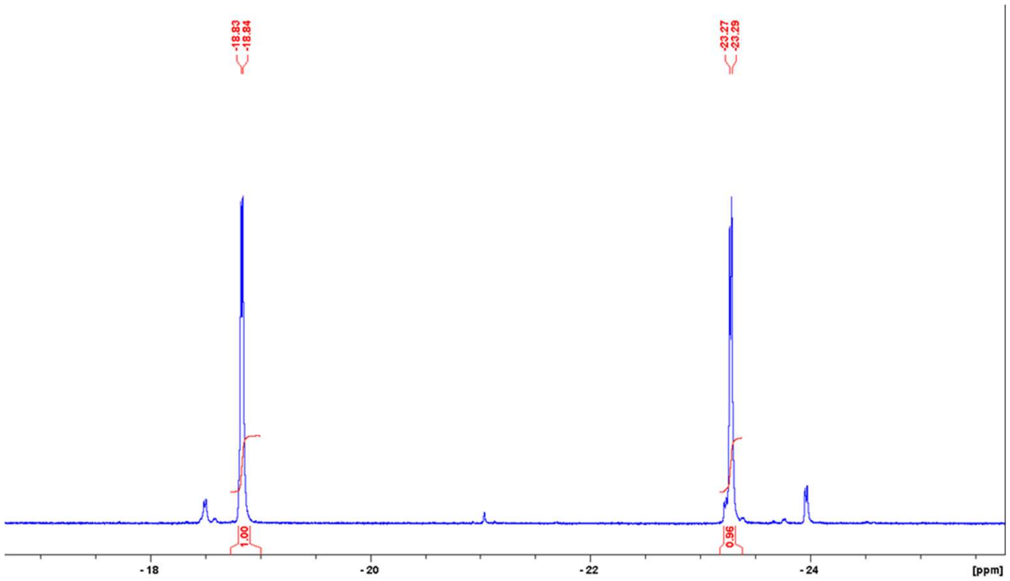

Figure S35. Hydride region of the ^{1}H NMR spectrum (400 MHz) of **4c** in CD_2Cl_2.

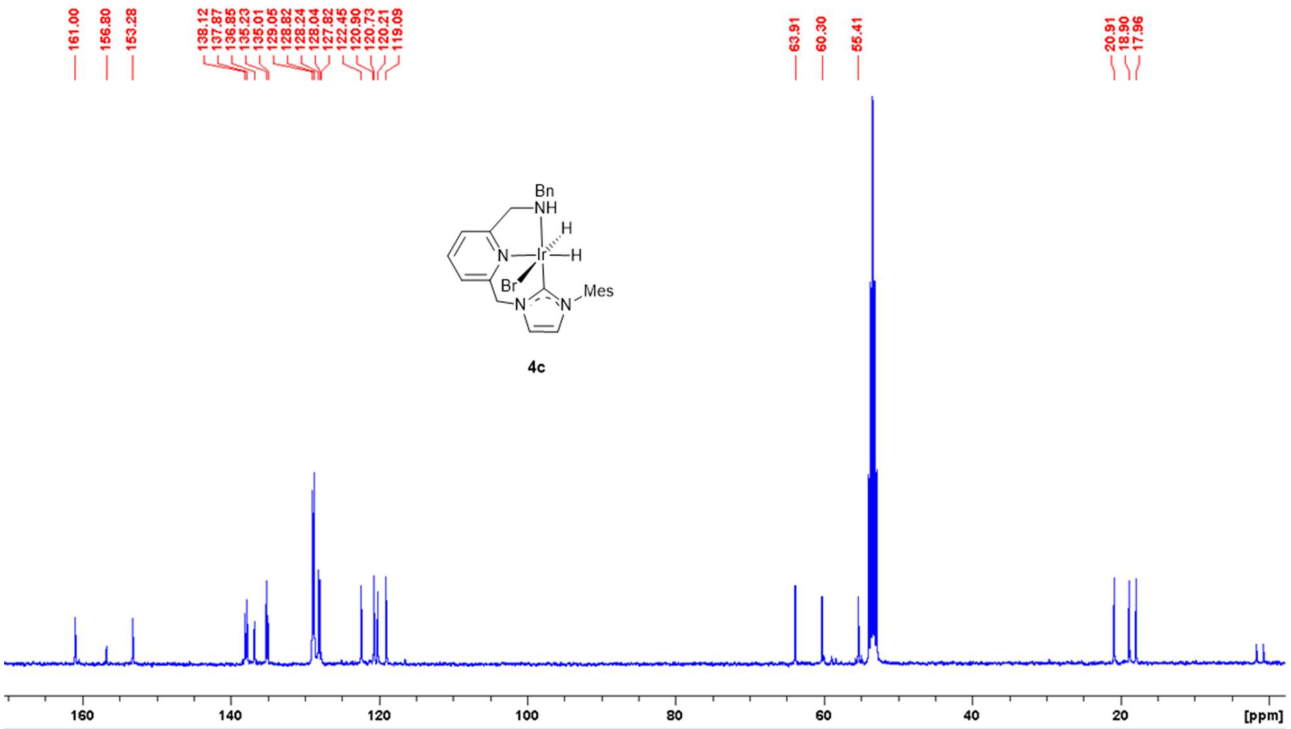

Figure S36. $^{13}C\{^{1}H\}$ NMR spectrum (101 MHz) of **4c** in CD_2Cl_2.

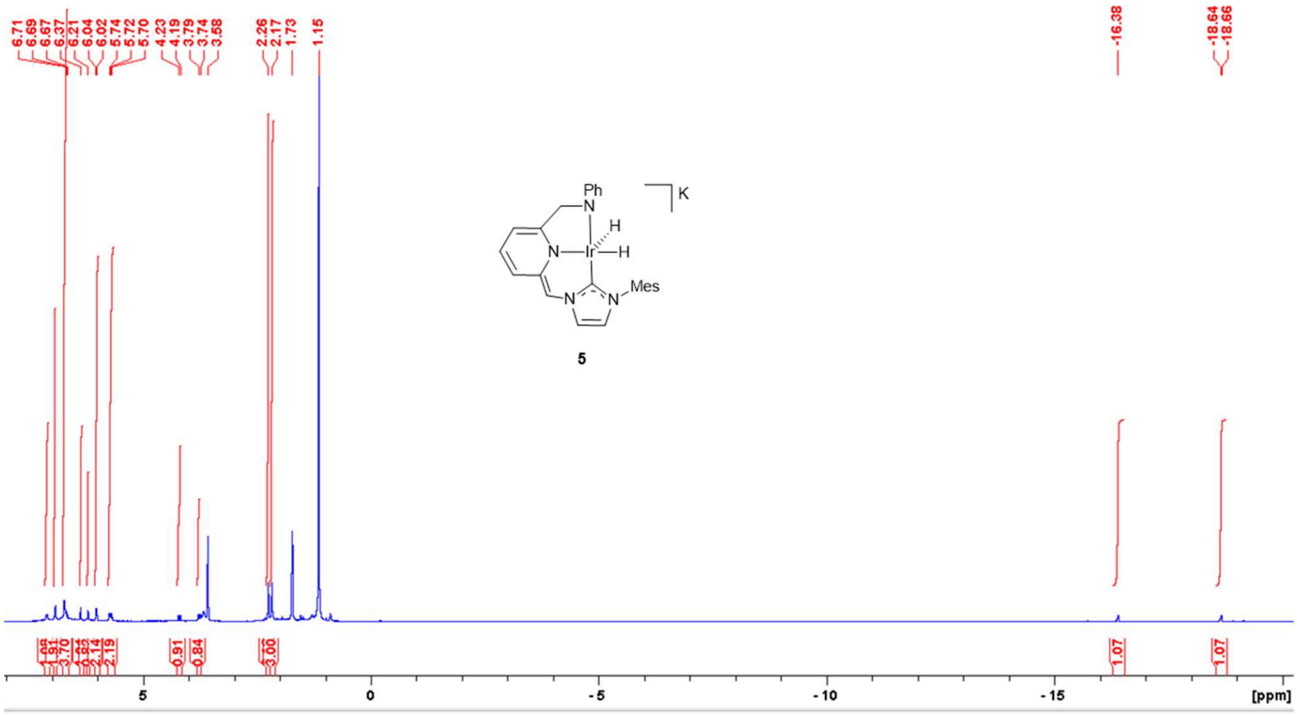

Figure S37. ^{1}H NMR spectrum (400 MHz, THF-d_8) of the reaction of **4a** with *t*BuOK (2.5 equiv) to yield **5**.

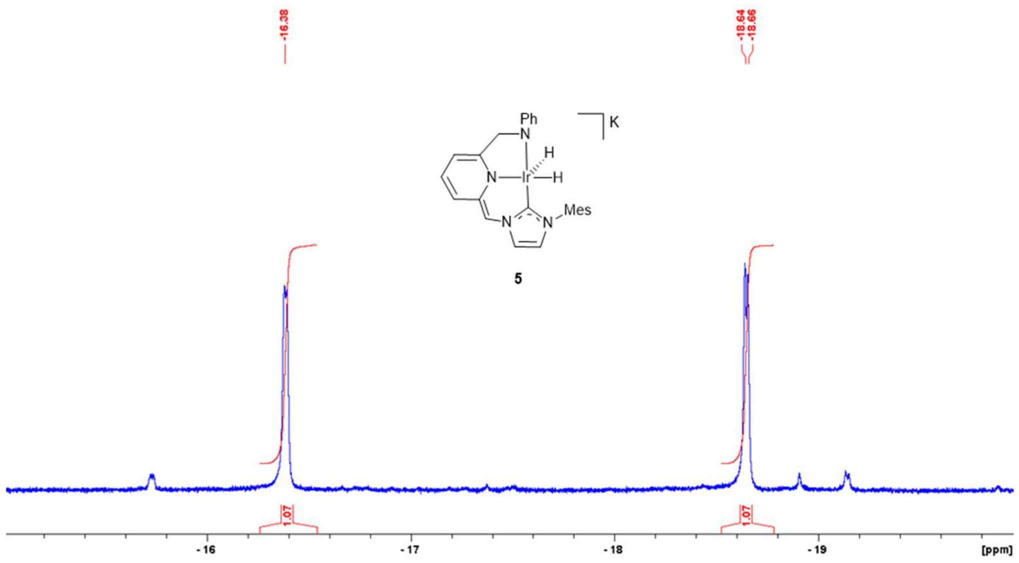

Figure S38. Hydride region of the ^{1}H NMR spectrum (400 MHz, THF-d_8) of the reaction of **4a** with *t*BuOK (2.5 equiv) to yield **5**.

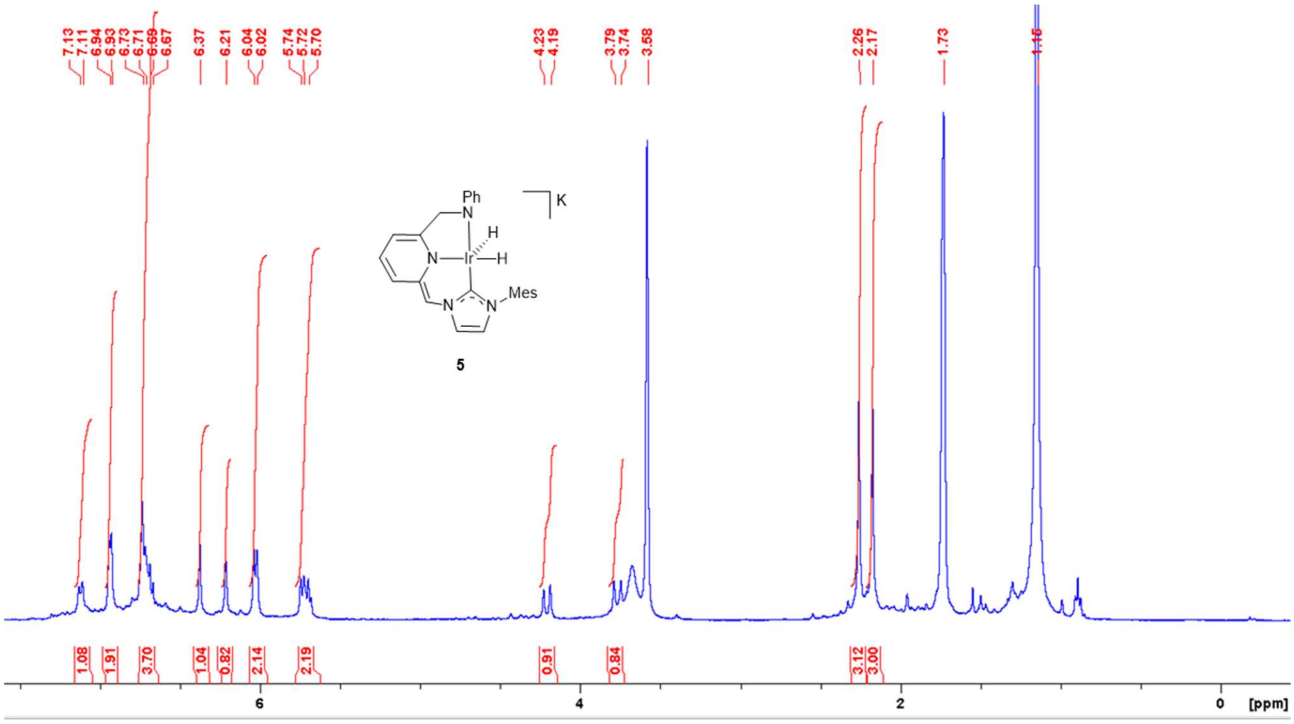

Figure S39. Region (0.0 to 8.5 ppm) of the ^{1}H NMR spectrum (400 MHz, THF-d_8) of the reaction of **4a** with *t*BuOK (2.5 equiv) to yield **5**.

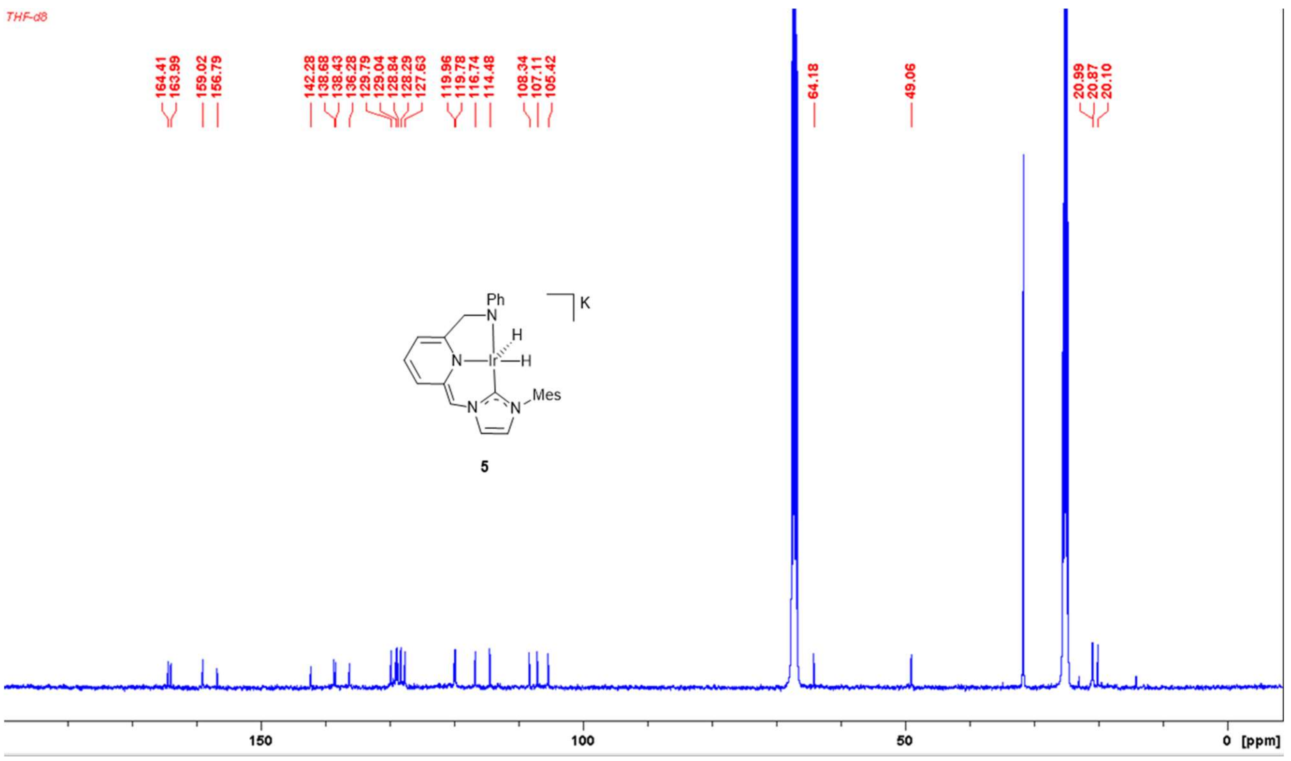

Figure S40. $^{13}C\{^1H\}$ NMR spectrum (101 MHz, THF-d_8) of the reaction of **4a** with *t*BuOK (2.5 equiv) to yield **5**.

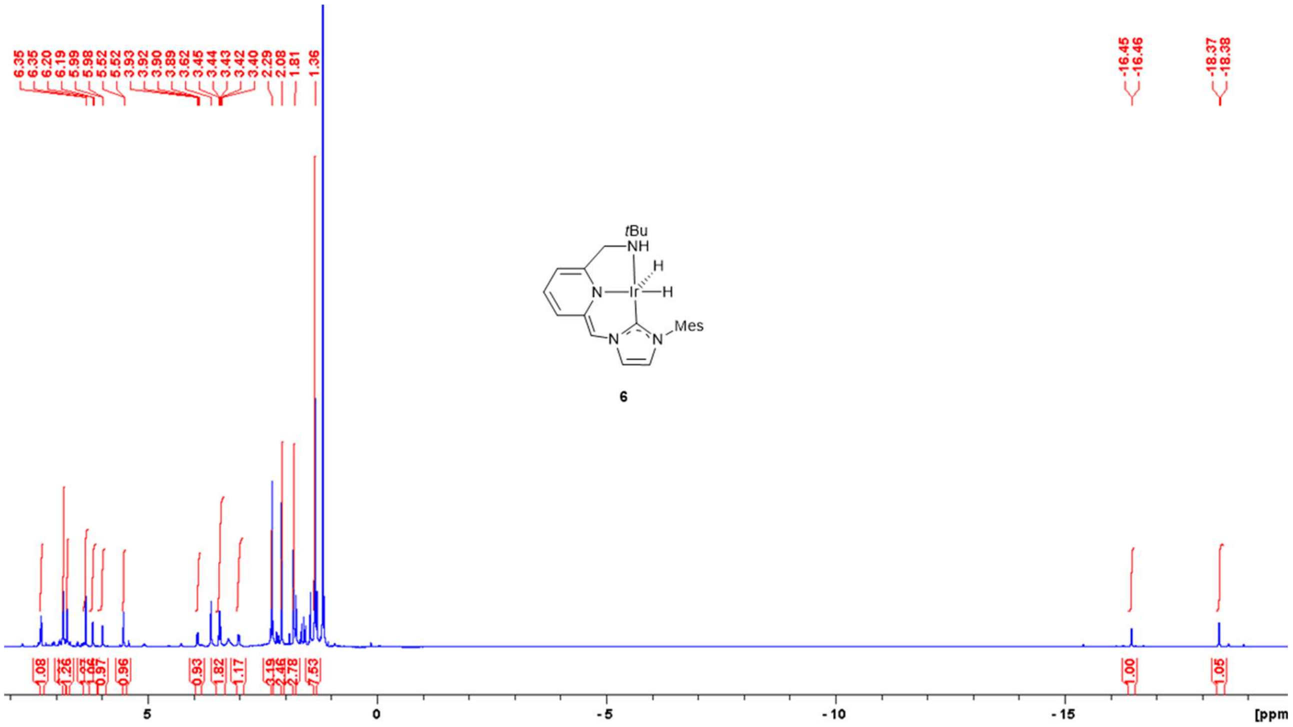

Figure S41. ^1H NMR spectrum (400 MHz, THF-d_8) of the reaction of **4b** with *t*BuOK (2.5 equiv) to yield **6**.

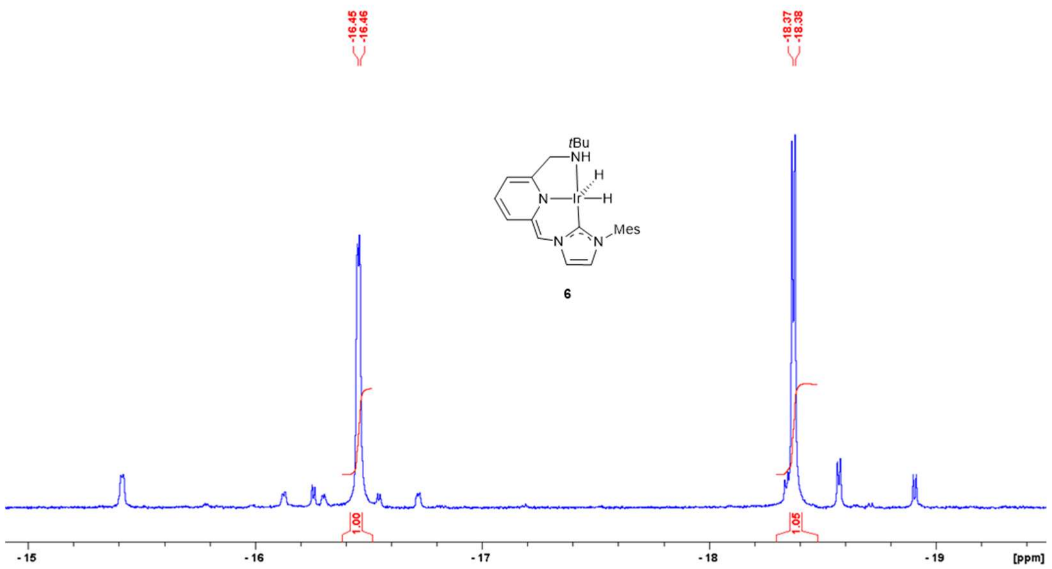

Figure S42. Hydride region of the ^{1}H NMR spectrum (400 MHz, THF-d_8) of the reaction of **4b** with *t*BuOK (2.5 equiv) to yield **6**.

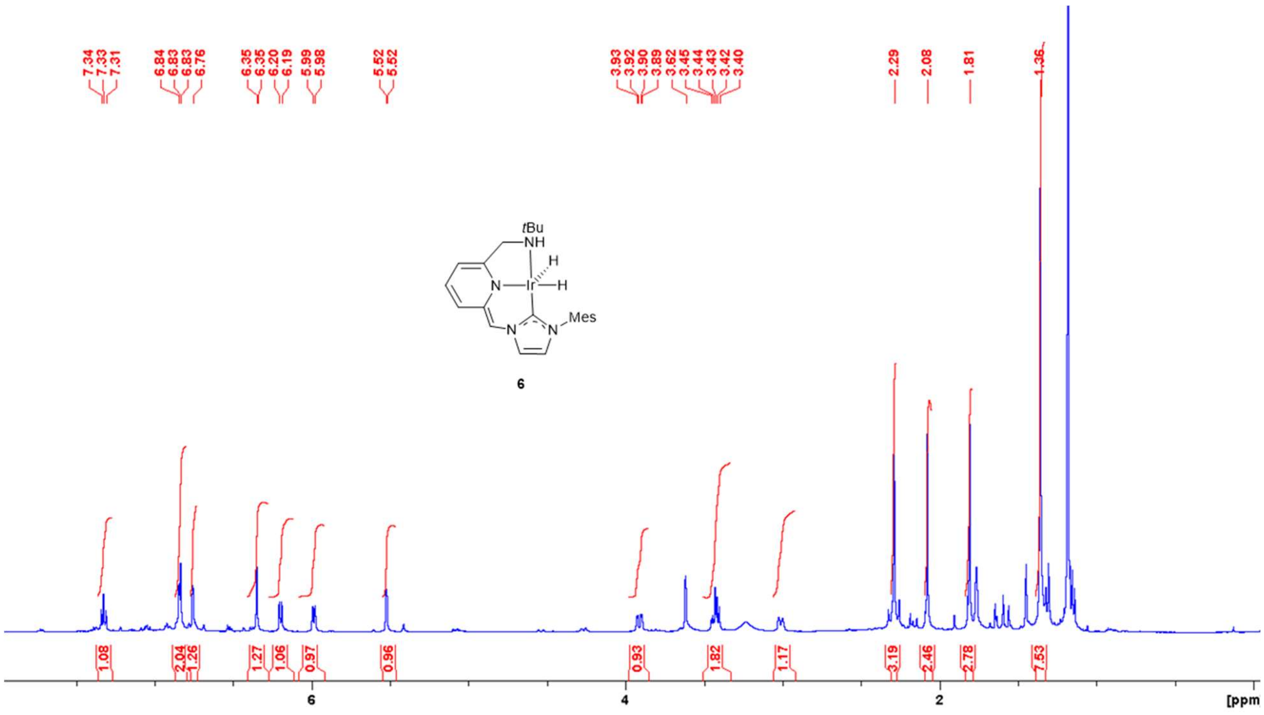

Figure S43. Region (0.0 to 8.0 ppm) of the ^{1}H NMR spectrum (400 MHz, THF-d_8) of the reaction of **4b** with *t*BuOK (2.5 equiv) to yield **6**.

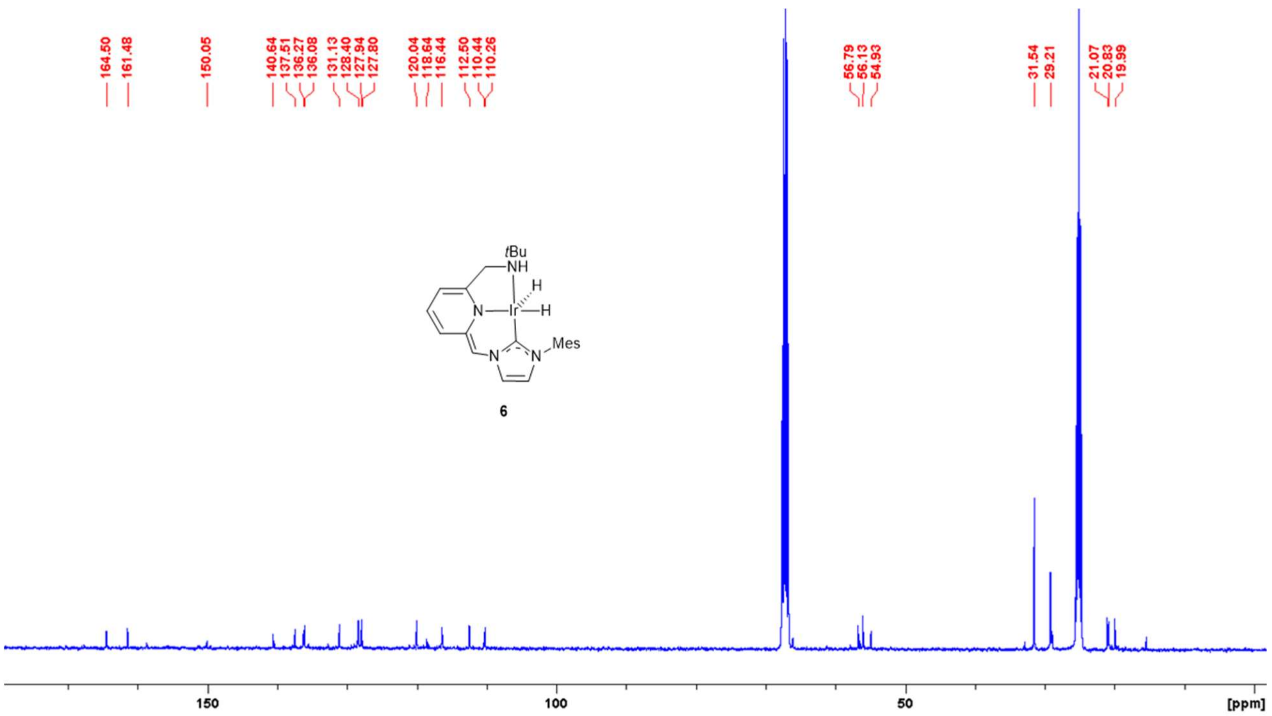

Figure 44. $^{13}C\{^{1}H\}$ NMR spectrum (101 MHz, THF-d_8) of the reaction of **4b** with *t*BuOK (2.5 equiv) to yield **6**.

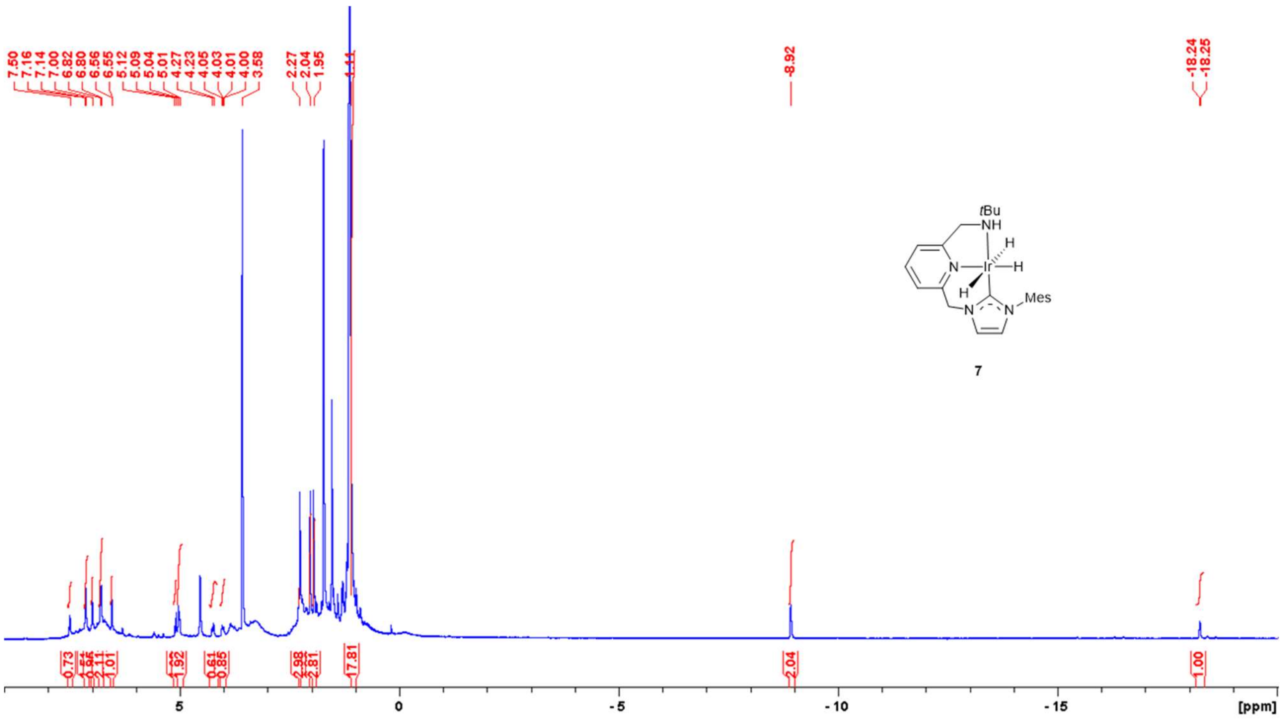

Figure S45. ^{1}H NMR spectrum (400 MHz, THF-d_8) of the reaction of **6** (formed *in situ* from **4b** and *t*BuOK) with H_2 (4 bar) to yield **7**.

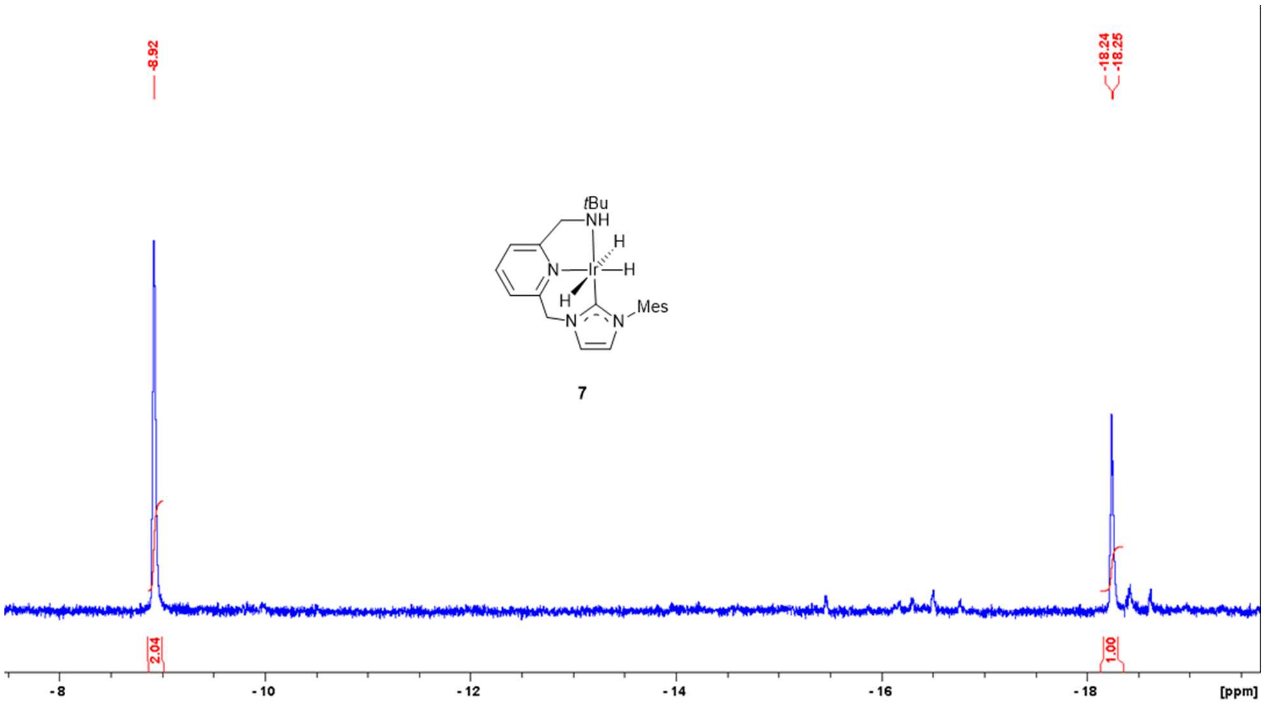

Figure S46. Hydride region of the ^{1}H NMR spectrum (400 MHz, THF-d_8) of the reaction of **6** (formed *in situ* from **4b** and *t*BuOK) with H_2 (4 bar) to yield **7**.

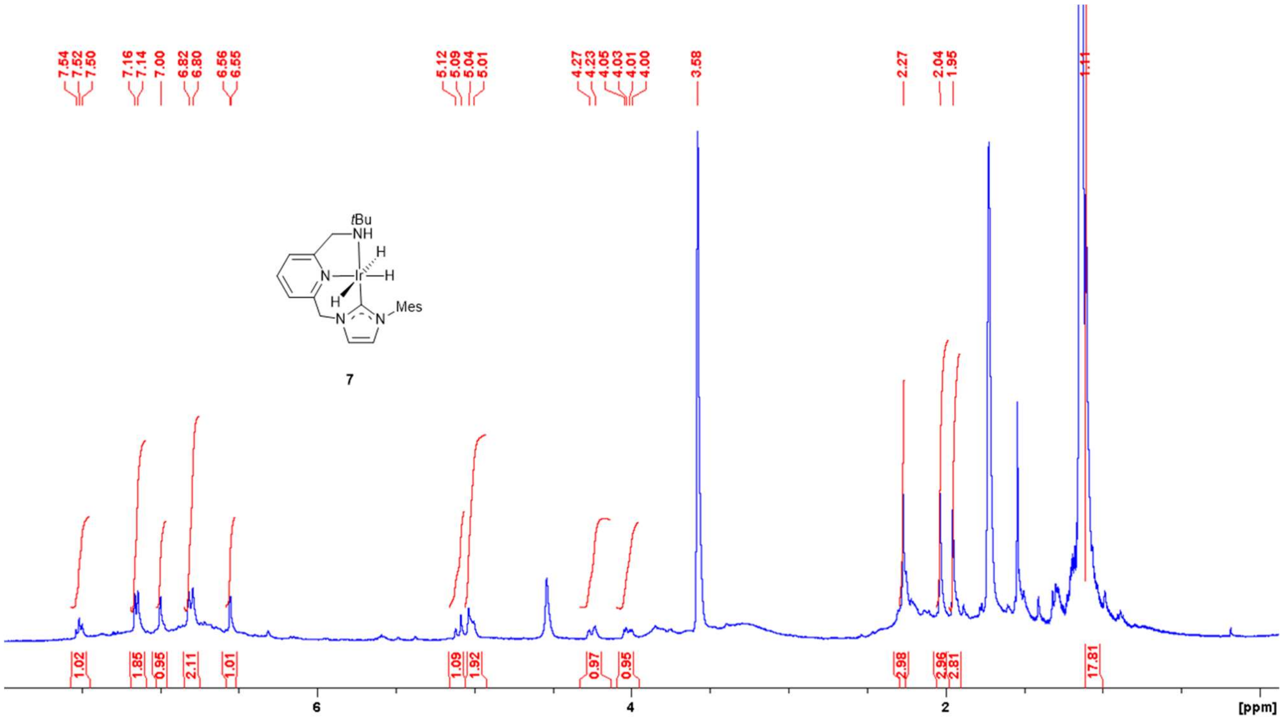

Figure S47. Region (0.0 to 8.0 ppm) of the ^{1}H NMR spectrum (400 MHz, THF-d_8) of the reaction of **6** (formed *in situ* from **4b** and *t*BuOK) with H_2 (4 bar) to yield **7**.

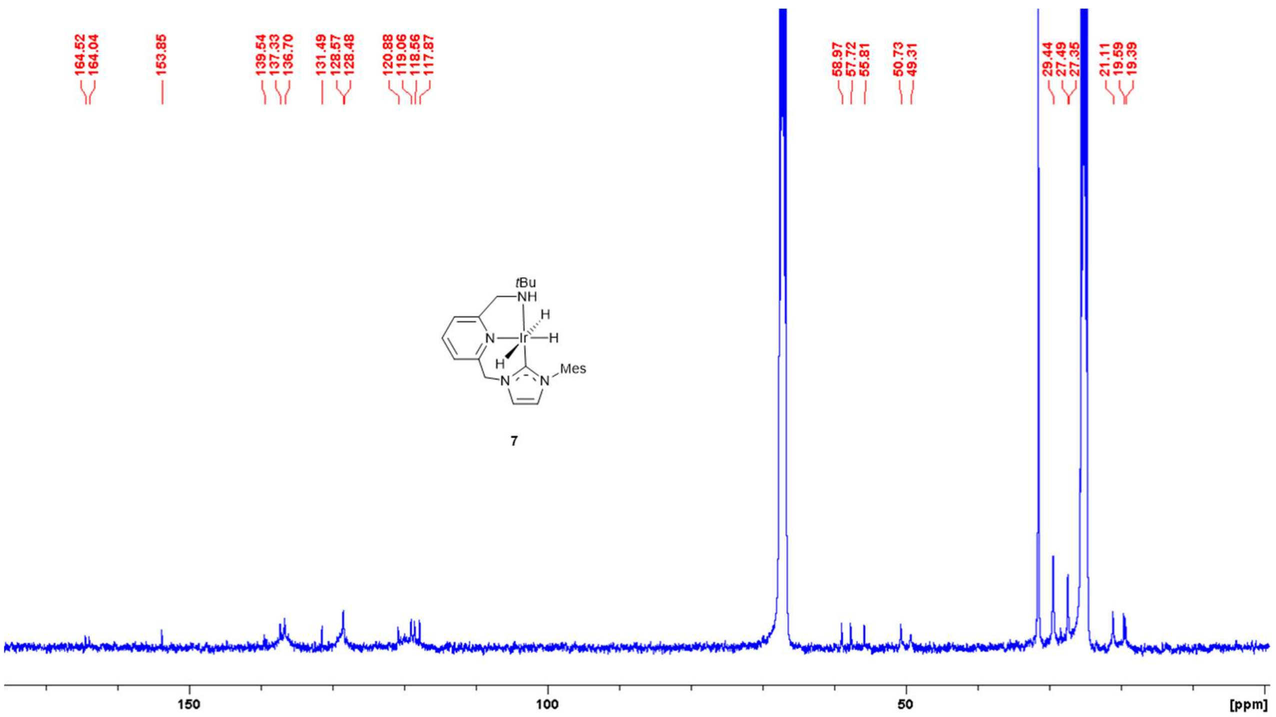

Figure S48. $^{13}C\{^{1}H\}$ NMR spectrum (101 MHz, THF-d_8) of the reaction of **6** (formed *in situ* from **4b** and *t*BuOK) with H_2 (4 bar) to yield **7**.

7. Comparison of the catalytic activity of 4a with previously reported catalysts

[Pd(allyl)][BF_4]

Michalak, Kang *et al.*[5]

3.0 mol% Pd

$MeNO_2$/tetraglyme; 25 °C

2 equiv. H_2; 20 sec

(closed system)

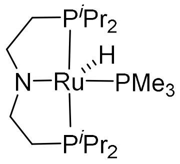

Schneider *et al.*[6]

0.1 mol% Ru

THF; r.t.

>1 equiv. H_2; <10 min

(eudiometric measurement)

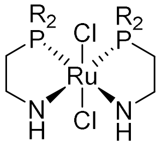

+ ^{t}BuOK

Fagnou *et al.*[7]

0.1 mol% Ru (3.0 mol% ^{t}BuOK)

THF; 22 °C

0.9-1.0 equiv. H_2; <5 min

(eudiometric measurement)

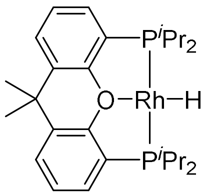

Esteruelas *et al.*[8]

1.0 mol% Rh

THF; 31 °C.

1.0 equiv. H_2; 3.5 min

(eudiometric measurement)

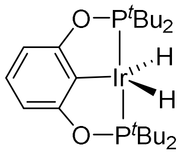

Heinekey, Goldberg *et al.*[9]

0.5 mol% Ir

THF; r.t.

1.0 equiv. H_2; 14 min

(eudiometric measurement)

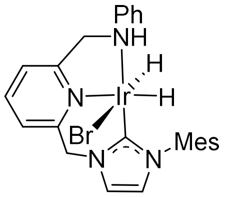

+ ^{t}BuOK

This work

0.4 mol% Ir (1.0 mol% ^{t}BuOK)

THF; r.t.

1.0 equiv. H_2; 8.5 min

(closed system)

---

[5] Kim, S.-K.; Han, W.-S.; Kim, T.-J.; Kim, T.-Y.; Nam, S. W.; Mitoraj, M.; Piekoś, Ł.; Michalak, A.; Hwang, S.-J.; Kang, S. O. *J. Am. Chem. Soc.* **2010**, *132*, 9954.

[6] Käβ, M.; Friedrich, A.; Drees, M.; Schneider, S. *Angew. Chem. Int. Ed.* **2009**, *48*, 905.

[7] Blaquiere, N.; Diallo-Garcia, S.; Gorelsky, S. I.; Black, D. A.; Fagnou, K. *J. Am. Chem. Soc.* **2008**, *130*, 14034.

[8] Esteruelas, M. A.; Nolis, P.; Oliván, M.; Oñate, E.; Vallribera, A.; Vélez, A. *Inorg. Chem.* **2016**, *55*, 7176.

[9] Denney, M. C.; Pons, V.; Hebden, T. J.; Heinekey, D. M.; Goldberg, K. I. *J. Am. Chem. Soc.* **2006**, *128*, 12048.

8. DFT calculations of the reaction catalyzed by 6

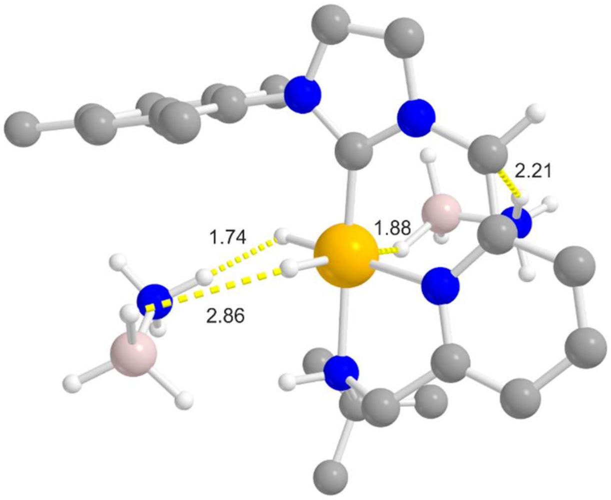

Figure S49. Optimized structure of the adduct between **6** and two AB molecules (**III**). Selected optimized bond lengths reported (Å). H atoms on the pincer ligand not relevant for the discussion are omitted for clarity. Atom color code: white, H; gray, C; blue, N; pink, B; orange, Ir. Mulliken charges on the hydride ligands in **6** are close to zero and weakly *positive*; most of the negative charge is located on the N atoms of the carbene and amino pincer substituents. Thus, the interaction with the second AB molecule through the metal hydrides is very weak.

The only path that provides meaningful Transition States is the same as that found for **4a**, *i.e.* AB bifunctional activation (on the iridium center and on the CH^- pincer side-arm to get the trihydride **7** and BH_2=NH_2) followed by intramolecular H_2 elimination from **7**. The corresponding Transition States **TS$_{3C}$** and **TS$_{4C}$** are reported in Figure S51a and S51b. If the "N-path" and the "C-path" are considered for **4a** and **4b** respectively, the $\Delta G^{\#}_{THF}$ values for the rate-determining step (H_2 elimination) are 8.5 (**TS$_{2N}$** in the main text) and 22.6 (**TS$_{4C}$**) kcal/mol. Accordingly, the reaction catalyzed by **4b** should take place at a lower rate, as observed experimentally. However, this mechanistic hypothesis is not in agreement with the experimentally determined second-order dependence of the reaction rate from AB.

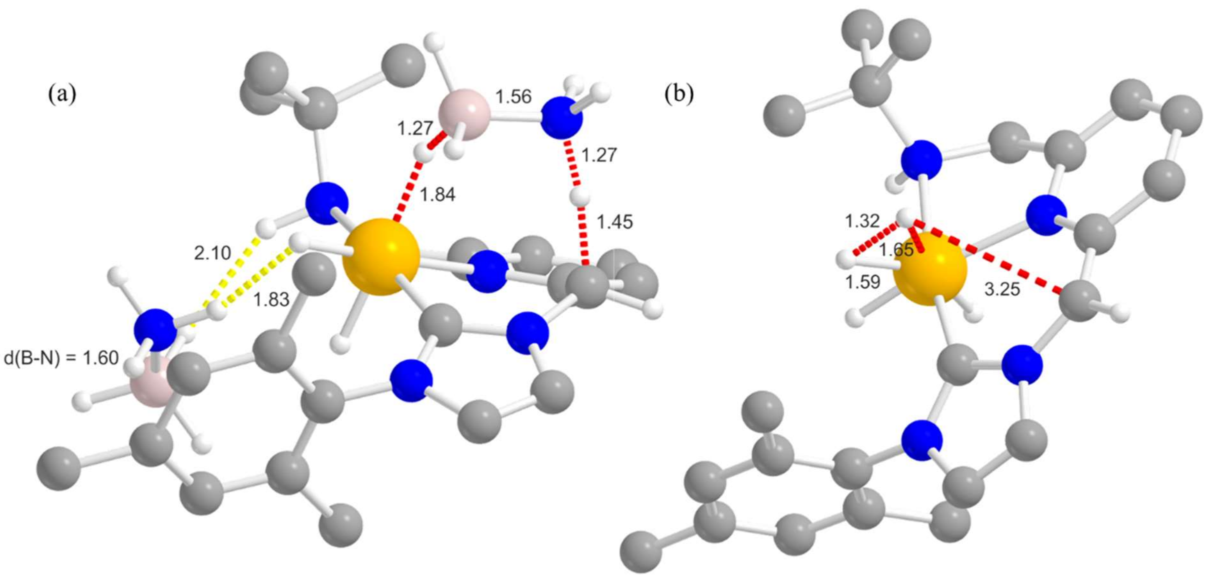

Figure S50. Optimized structures of (a) **TS$_{3C}$** and (b) **TS$_{4C}$**. Selected optimized bond lengths reported (Å). H atoms on the pincer ligand not relevant for the discussion omitted for clarity. Bonds involved in the TS transformation depicted in red dotted lines. Atom color code: see Figure S50.

9. Crystal X-ray structure analysis for 3, 4b and 5

Crystals of suitable size for X-ray diffraction analysis were coated with dry perfluoropolyether and mounted on glass fibers and fixed in a cold nitrogen stream (T = 193.15 K) to the goniometer head. Data collection was performed on a Bruker-Nonius X8Apex-II CCD (**3** and **4b**) and on a Bruker-AXS, D8 QUEST ECO, PHOTON II area detector (**5**) diffractometers, using monochromatic radiation λ(Mo K_{α}) = 0.71073 Å and , by means of ω and φ scans with a width of 0.50 degrees. The data were reduced (SAINT)[10] and corrected for absorption effects by the multi-scan method (SADABS).[11] The structures were solved by direct methods (SIR-2002)[12] and refined against all F^2 data by full-matrix least-squares techniques [*SHELXL*-2016/6 (**3**) and *SHELXL*-2018/3 (**4b** and **5**)][13] minimizing $w[F_o^2-F_c^2]^2$. All the non-hydrogen atoms were refined anisotropically, while C-H hydrogen atoms were placed in geometrically calculated positions using a riding model. The hydrogen atoms were included from calculated positions and refined riding on their respective carbon atoms with isotropic displacement parameters. The hydride ligands for these compounds were not located crystallographically, but predicted by potential energy calculations using the HYDEX[14] program. All the hydride ligand positions were included as fixed contributions and refined isotropically. For the dimeric potassium iridate complex **5**, some geometric restraints (SADI and DFIX commands), the ADP restraint SIMU and the rigid bond restraint DELU were used to make the geometric and ADP values of the disordered atoms more reasonable. A search for solvent accessible voids for this crystal structure **5** using *SQUEEZE*[15] showed two small volumes of potential solvents of 161 Å^3 for each (49 electron count for each), whose solvent content could not be identified or refined with the most severe restraints. The

---

[10] Bruker. *APEX2* and *APEX3*. Bruker AXS Inc., Madison, Wisconsin, USA. **2012.**

[11] Bruker Advanced X-ray solutions. *SAINT* and *SADABS* programs. Bruker AXS Inc., Madison, Wisconsin, USA**. 2001**.

[12] Burla, M. C.; Camalli, M.; Carrozzini, B.; Cascarano, G. L.; Giacovazzo, C.; Polidori, G.; Spagna, R. *J. Appl. Cryst.* **2003**, *36*, 1103.

[13] Sheldrick, G. M. *Acta Crystallogr., Sect. A* **2008**, *64*, 112.

[14] Orpen, A. G. J. *Chem. Soc., Dalton Trans.* **1980**, 2509.

[15] Sluis, P. v.d.; Spek, A. L. *Acta Crystallogr., Sect. A* **1990**, *46*, 194.

corresponding CIF data represent *SQUEEZE* treated structures with the solvent molecules handling as a diffuse contribution to the overall scattering, without specific atom position and excluded from the structural model. The *SQUEEZE* results were appended to the CIF. The corresponding crystallographic data were deposited with the Cambridge Crystallographic Data Centre as supplementary publications. CCDC 2113023 (**3**), 2113024 (**4b**) and 2113025 (**5**). The data can be obtained free of charge *via:* https://www.ccdc.cam.ac.uk/structures/

A summary of cell parameters, data collection, structures solution, and the refinement of crystal structures are provided below.

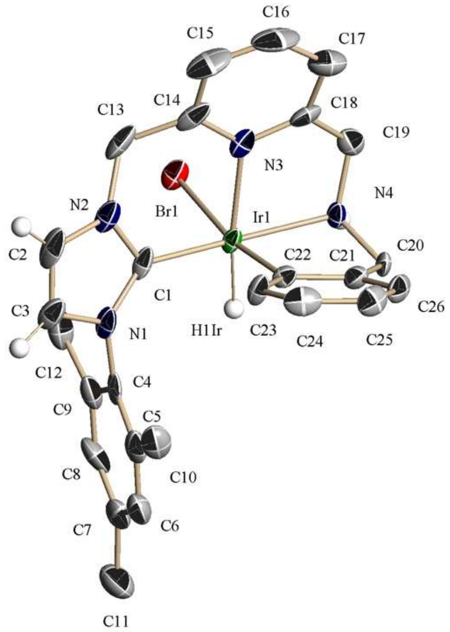

Figure S51. ORTEP view of molecular structure of iridium complex **3** with thermal ellipsoids drawn at the 30% level. Hydrogen atoms, except for the hydride ligand and the NHC hydrogens, have been omitted for clarity.

Table S2. Crystal data and structure refinement for **3**.

Empirical formula	$C_{26}H_{28}BrIrN_4$	
Formula weight	668.63	
Temperature	193(2) K	
Wavelength	0.71073 Å	
Crystal system	Triclinic	
Space group	P $\bar{1}$	
Unit cell dimensions	a = 12.9967(5) Å	α= 95.846(2)°.
b = 16.8903(5) Å	β= 99.147(2)°.	
c = 23.3533(8) Å	γ = 96.061(2)°.	
Volume	4996.1(3) $Å^3$	
Z	8	
Density (calculated)	1.778 Mg/m^3	
Absorption coefficient	6.965 mm^{-1}	
F(000)	2592	
Crystal size	0.100 x 0.050 x 0.050 mm^3	
Theta range for data collection	1.221 to 25.249°.	
Index ranges	-15<=h<=15, -20<=k<=20, -28<=l<=28	
Reflections collected	66649	
Independent reflections	18092 [R(int) = 0.0881]	
Completeness to theta = 25.242°	99.9 %	
Absorption correction	Semi-empirical from equivalents	
Max. and min. transmission	0.7221 and 0.5426	
Refinement method	Full-matrix-block least-squares on F^2	
Data / restraints / parameters	18092 / 0 / 1165	
Goodness-of-fit on F^2	0.965	
Final R indices [I>2sigma(I)]	R1 = 0.0508, wR2 = 0.1087	
R indices (all data)	R1 = 0.1028, wR2 = 0.1215	
Extinction coefficient	n/a	
Largest diff. peak and hole	2.326 and -1.356 e.$Å^{-3}$	

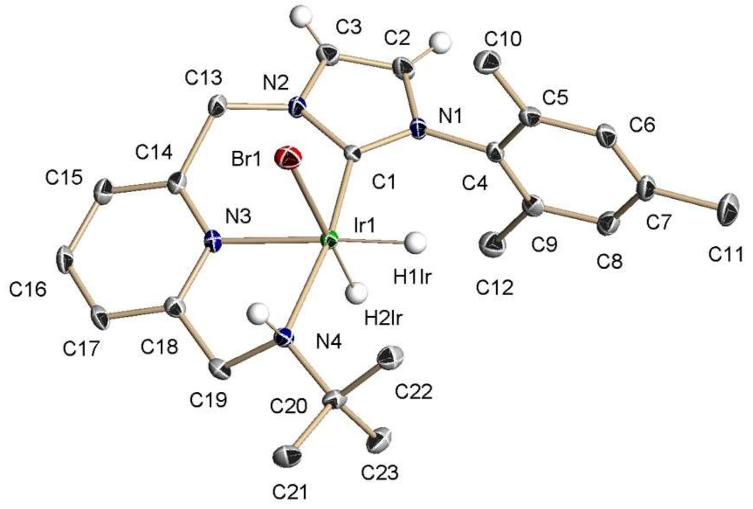

Figure S52. ORTEP view of molecular structure of iridium complex **4b** with thermal ellipsoids drawn at the 30% level. Hydrogen atoms, except for the hydride ligand and the NHC and NH hydrogens, have been omitted for clarity.

Table S3. Crystal data and structure refinement for **4b**.

Empirical formula	$C_{23}H_{32}BrIrN_4$	
Formula weight	1273.27	
Temperature	193(2) K	
Wavelength	0.71073 Å	
Crystal system	Monoclinic	
Space group	$P2_1/n$	
Unit cell dimensions	a = 9.9403(7) Å	α= 90°.
	b = 18.1229(12) Å	β= 103.418(4)°.
	c = 13.2667(9) Å	γ = 90°.
Volume	2324.7(3) $Å^3$	
Z	4	
Density (calculated)	1.819 Mg/m^3	
Absorption coefficient	7.478 mm^{-1}	
F(000)	1240	
Crystal size	0.100 x 0.050 x 0.030 mm^3	
Theta range for data collection	1.937 to 25.250°.	
Index ranges	-10<=h<=11, -21<=k<=20, -14<=l<=15	
Reflections collected	16236	
Independent reflections	4193 [R(int) = 0.0264]	
Completeness to theta = 25.242°	99.7 %	
Absorption correction	Semi-empirical from equivalents	
Max. and min. transmission	0.7461 and 0.6274	
Refinement method	Full-matrix least-squares on F^2	
Data / restraints / parameters	4193 / 2 / 274	
Goodness-of-fit on F^2	1.042	
Final R indices [I>2sigma(I)]	R1 = 0.0203, wR2 = 0.0465	
R indices (all data)	R1 = 0.0262, wR2 = 0.0483	
Extinction coefficient	n/a	
Largest diff. peak and hole	0.649 and -0.418 e.$Å^{-3}$	

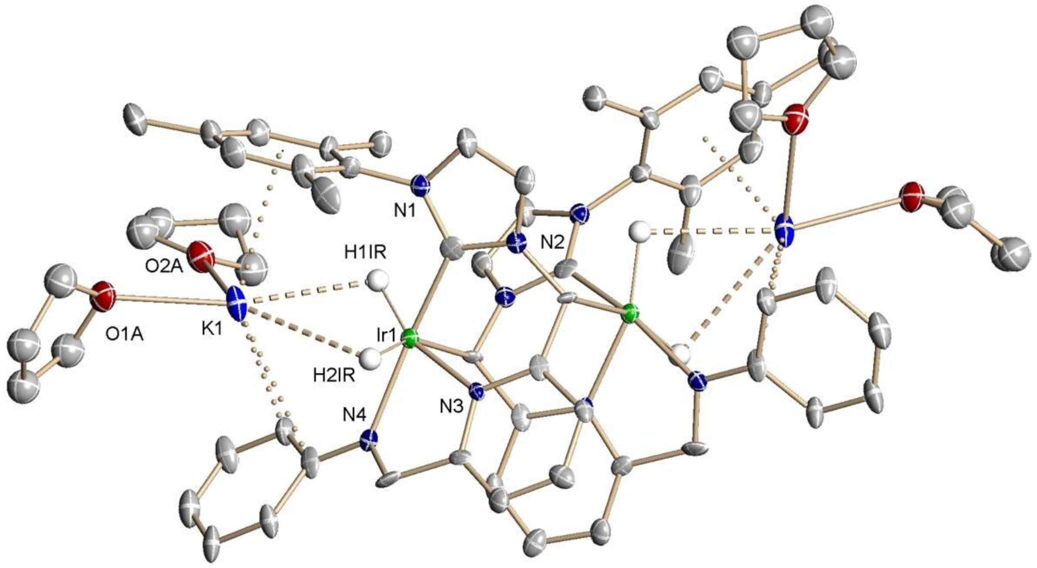

Figure S53. ORTEP view of molecular structure of iridium complex **5** with thermal ellipsoids drawn at the 30% level. Hydrogen atoms, except for the hydride ligands, have been omitted for clarity.

Table S4. Crystal data and structure refinement for **5**.

Empirical formula	$C_{66}H_{84}Ir_2K_2N_8O_4$	
Formula weight	1516.01	
Temperature	193(2) K	
Wavelength	0.71073 Å	
Crystal system	Monoclinic	
Space group	P2/n	
Unit cell dimensions	a = 15.275(3) Å	α= 90°.
	b = 10.925(3) Å	β= 93.619(9)°.
	c = 20.535(5) Å	γ = 90°.
Volume	3420.0(14) $Å^3$	
Z	2	
Density (calculated)	1.472 Mg/m^3	
Absorption coefficient	4.058 mm^{-1}	
F(000)	1520	
Crystal size	0.300 x 0.120 x 0.100 mm^3	
Theta range for data collection	2.112 to 25.249°.	
Index ranges	-18<=h<=18, -13<=k<=13, -24<=l<=24	
Reflections collected	65449	
Independent reflections	6179 [R(int) = 0.1502]	
Completeness to theta = 25.242°	99.7 %	
Absorption correction	Semi-empirical from equivalents	
Max. and min. transmission	0.7461 and 0.5923	
Refinement method	Full-matrix least-squares on F^2	
Data / restraints / parameters	6179 / 394 / 471	
Goodness-of-fit on F^2	1.116	
Final R indices [I>2sigma(I)]	R1 = 0.1078, wR2 = 0.2219	
R indices (all data)	R1 = 0.1543, wR2 = 0.2394	
Extinction coefficient	n/a	
Largest diff. peak and hole	9.881 and -3.502 e.$Å^{-3}$	
